# A Fenton Approach to Aromatic Radical Cations and Diarylmethane Synthesis

**DOI:** 10.1021/acs.joc.3c01505

**Published:** 2023-10-17

**Authors:** Robert Crowley III, Berkley Lujan, Alex Martinez, Roni Manasi, Justin D. DeBow, Kevin G. M. Kou

**Affiliations:** Department of Chemistry, University of California, Riverside, 501 Big Springs Road, Riverside, California 92521, United States

## Abstract

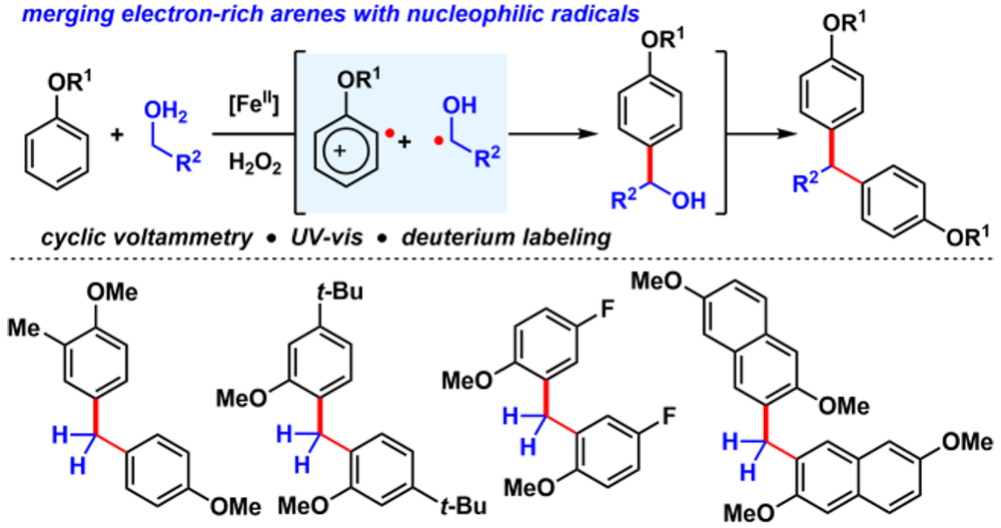

Manipulating carbon-centered radicals to add to electron-deficient systems is a well-precedented process. By coupling the Fe(II)-mediated Fenton reaction with the Fe(III)-mediated single-electron oxidation of anisolic compounds, we demonstrate how electron-rich carbon-centered radicals can react with electron-rich arenes through a radical-polar cascade pathway. This bioinspired approach produces diarylmethane derivatives from simple unfunctionalized precursors.

## Introduction

The Fenton reaction describes the iron(II)-mediated decomposition of hydrogen peroxide to hydroxyl radical,^1^ a reactive oxygen species implicated in various oxidative stress processes that are associated with neurodegenerative^2^ and cardiovascular disorders,^3^ as well as aging^4^ and cancer.^5^ The highly reactive nature of hydroxyl radicals toward organic matter even renders the Fenton method effective for wastewater treatment.^6^ This reactivity has been elegantly exploited for organic synthesis in the context of selective carbon–hydrogen (C–H) oxidation reactions to construct new carbon–oxygen (C–O),^7^ carbon–sulfur (C–S),^8^ and carbon–fluorine (C–F)^9^ bonds. Considering the effectiveness in Nature’s use of oxidative, single-electron pathways to assemble the carbon–carbon (C–C) frameworks of polyphenolic,^10^ lignin,^11^ and tripyrrole natural products,^12^ and building on our lab’s interest in directly functionalizing alcohols,^13^ we sought to explore the oxidative capacity of Fenton’s reagent for direct C(sp^2^)–C(sp^3^) bond formations.

**Scheme 1:**
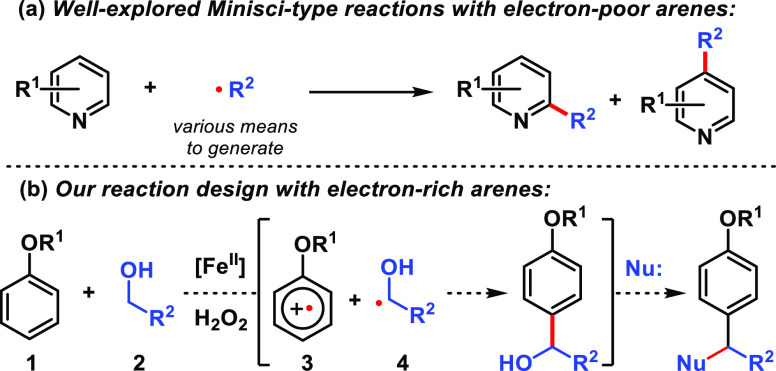
(a) The Minisci Reaction; (b) Our Strategy for Reacting Anisole Derivatives

Complementary to classical 2-electron transition metal catalysis and polar addition reactions, the distinct chemistry of carbon-centered radicals offers unconventional approaches to cross-coupling^14^ and C–H functionalization^15^ processes. A fruitful area of development involves transformations of carbon-centered radicals with largely electron-deficient heterocycles (i.e., the Minisci reaction)^15a−15c,16^ and electron-deficient alkenes (Scheme 1a).^17^ However, the analogous capture of radical intermediates with electron-rich fragments is unusual. Examples of adding arene radicals,^18^ as well as electrophilic alkyl radicals^19^ to electron-rich arenes have been reported. Herein we report the coupling of anisole derivatives with a nucleophilic radical derived from methanol under Fenton conditions.

The reaction design relies on the Fe(II) to Fe(III) Fenton pathway^1^ to convert alcohols **2** to hydroxyalkyl radicals **4** (Scheme 1b). Our approach to merge nucleophilic radicals with electron-rich arenes **1** is to render the latter electron-deficient through their interaction with the Fe(III) species produced in the Fenton process. Because radical cation species akin to **3** have been isolated and characterized by X-ray crystallography,^20a^ its persistence should allow for radical–radical recombination between intermediates **3** and **4**, ultimately enabling cross-coupling between an sp^2^-hybridized carbon of anisole with the sp^3^-hybridized carbon adjacent to alcohols. This hypothesis presented an opportunity to advance our knowledge of the chemical reactivity of radical cations, which, despite significant progress,^20^ is still largely confined to homo-/heterodimerization pathways to furnish biaryls^21^ and reactions with alkenes.^22^

## Results and Discussion

The target reactivity is realized when 2-methylanisole (**1a**) is subjected to methanol in the presence of iron(II) sulfate (1 equiv), hydrogen peroxide (3.5 equiv), and sulfuric acid (20 equiv) (Table 1). ***Caution!****Piranha solution, the mixture of sulfuric acid with H*_*2*_*O*_*2*_*, is a strong oxidizing agent, which can react violently with most organic materials and must be handled with extreme care*.^23^ Precooling the reaction mixtures in an ice bath prior to introducing H_2_O_2_ is important for preventing effervescence (see Supporting Information (SI)). Under these conditions, dianisylmethane **6a** is formed in 93% NMR yield (84% isolated yield, entry 1), presumably through the intermediacy of benzylic alcohol **5a**, which immediately undergoes acid-catalyzed benzylic arylation.^24^ Iron(II) was essential for the observed reactivity, as substitution for Fe(III) sources such as Fe(acac)_3_, FeTPPCl, and Fe_2_(SO_4_)_3_ all resulted in significantly diminished yields (8–13%, entries 2–4). Iron halides do not participate in the desired reactivity and instead promote electrophilic halogenations under the oxidative conditions (see SI). Reducing the amount of iron to 0.5 equiv resulted in a 48% yield of dianisylmethane **6a** (entry 5), suggestive of the need for stoichiometric iron, and its absence halted reactivity (entry 6). Similarly, administering less H_2_O_2_ oxidant leads to lower product yield (entry 7) and no product is obtained when the oxidant is completely omitted (entry 8). The strong acid plays important roles in solubilizing the iron reagent and in favoring hydroxyl radical over oxoiron(IV) species as the reactive oxygen species.^25^ In this system, lowering the amount of H_2_SO_4_ to 10 equiv severely decreases the conversion to product (entry 9) and no reaction occurs in the absence of the acid (entry 10). The reaction is optimal when the alcohol is employed as the solvent. Diluting methanol in DCE, 1,4-dioxane, HFIP, or H_2_O as solvents is poorly productive (entries 11–14).

**Table 1:**
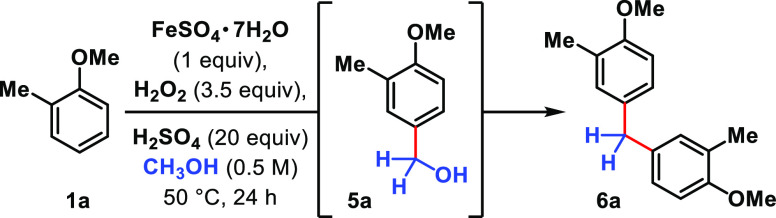

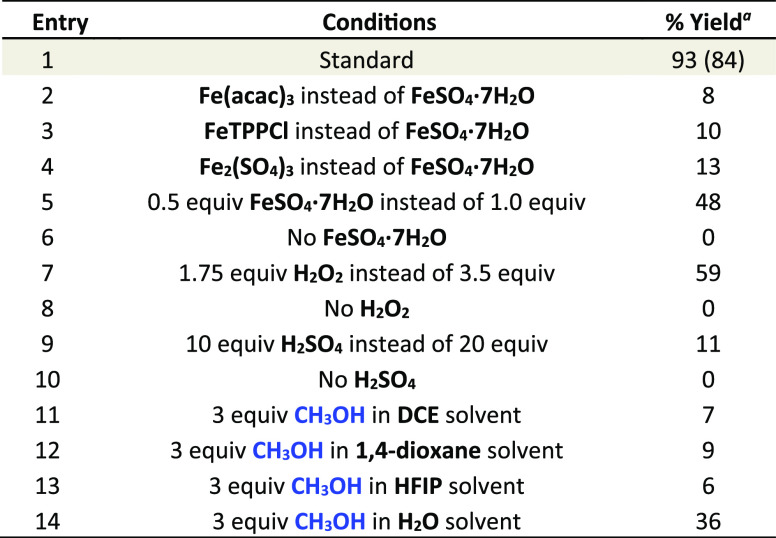
Effect of Reaction Parameters on Diarylmethane Synthesis

a Yields are determined by ^1^H NMR analysis of the crude reaction mixtures using mesitylene as the internal standard and are calculated based on 2 equiv of arene forming 1 equiv of product. Yield in parentheses represents isolated yield. TPP = tetraphenylporphyrin.

This method represents a direct synthesis of symmetrical dianisylmethane derivatives (Table 2). *ortho*-Substituted anisole derivatives **1** are transformed into dianisylmethane derivatives **6** with complete site-selectivity. 2-Methyl- and 2-ethylanisole reacts with methanol to yield dianisylmethanes **6a** and **6b** in 84% and 68% yields, respectively. The yield is consistent on a larger 2 mmol scale, where dianisylmethane **6a** was isolated in 86% yield. Product **6c** arising from 2-allylanisole is formed in a 36% yield. Substrates halogenated at the *ortho*-position can be transformed into dianisylmethane products **6d** and **6e**, with 2-bromo-anisole (45%) performing better than 2-fluoroanisole (29%). Dihydrobenzofuran reacted analogously to 2-methylanisole, producing diarylmethane **6f** in 73% yield. Veratrole is poorly reactive under the reaction conditions, and diarylmethane **6g** forms in 18% yield. In the lower yielding cases, including 2-allylanisole, a single isomer is formed. Benzylic alcohol intermediates were not observed in any of the crude reaction mixtures.

**Table 2:**
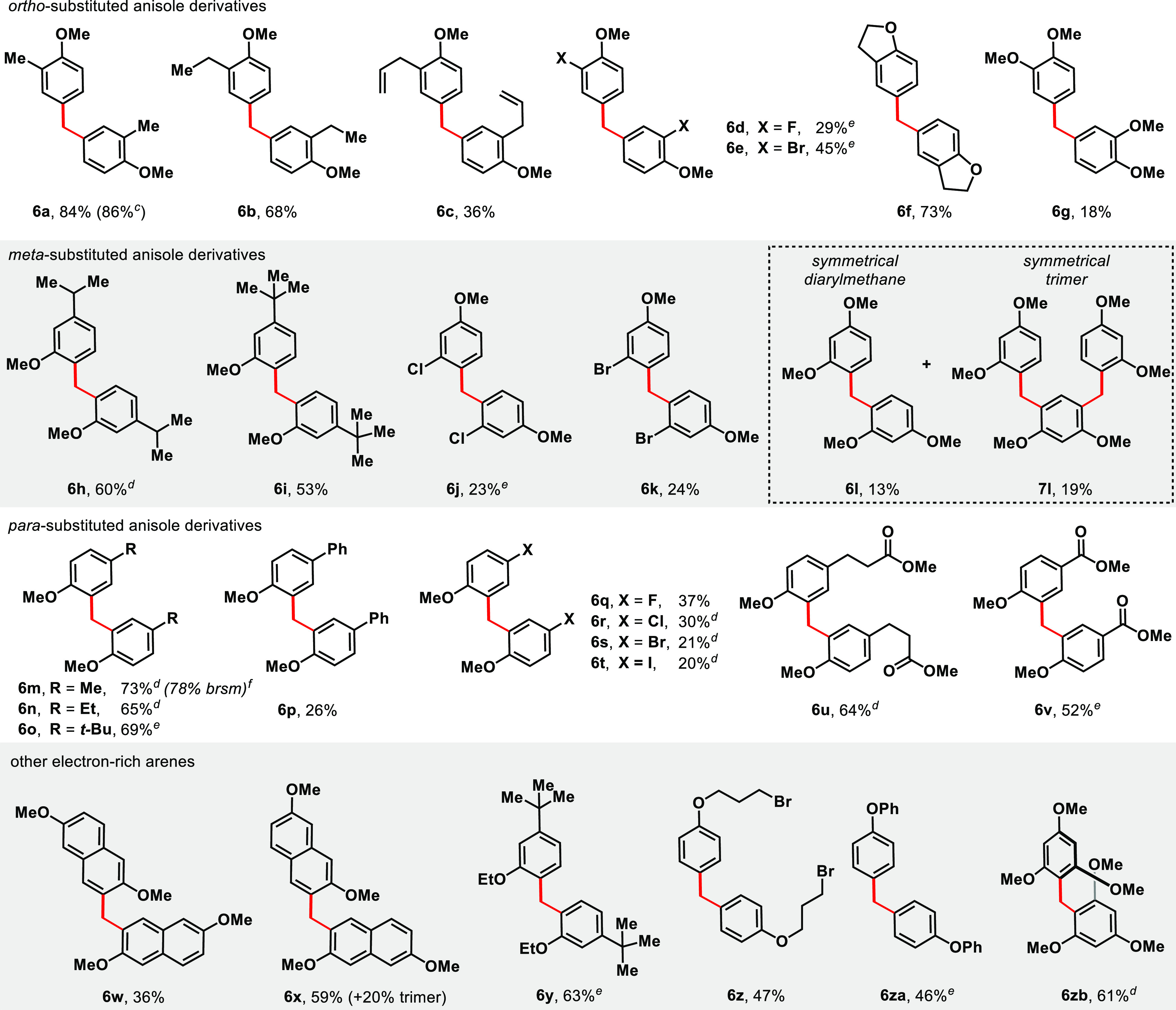
Scope of Diarylmethane Synthesis^a^^,^^b^

a Yields are determined based on 2 equiv of arene forming 1 equiv of product.

b Conditions: anisolic **1** (0.2 mmol), FeSO_4_·7H_2_O (0.2 mmol), H_2_O_2(aq)_ (0.7 mmol), H_2_SO_4_ (4 mmol), MeOH (0.4 mL, 0.5 M), 50 °C, 24 h.

c anisolic **1** (2 mmol), FeSO_4_·7H_2_O (2 mmol), H_2_O_2(aq)_ (7 mmol), H_2_SO_4_ (40 mmol), MeOH (4 mL, 0.5 M), 50 °C, 48 h.

d 50 °C, 48–72 h.

e 75 °C, 18–48 h.

f 75 °C, 72 h. *brsm* = based on recovered starting material.

Anisole derivatives with sterically encumbered substituents like isopropyl and *tert*-butyl are selectively alkylated at the less hindered position *ortho* to the methoxy group, producing diarylmethanes **6h** in 60% yield and **6i** in 53% yield. In contrast, 3-haloanisoles preferentially alkylate *para*- to the electron-releasing methoxy group to furnish symmetrical **6j** and **6k** (23–24% yields). Small amounts (<5%) of the isomers arising from *ortho*-/*ortho*-alkylation were also isolated in these cases (see SI). 1,3-Dimethoxybenzene reacts with methanol to forge 13% of diarylmethane **6l** as well as 19% of the trimer (**7l**). With respect to *para*-substituted anisolic derivatives, 4-methyl, 4-ethyl-, and 4-*tert*-butylanisole dimerizes to give **6m**–**6o** in good yields (65–73%). 4-Phenylanisole is reactive, albeit yielding only 26% of dianisylmethane **6p**. Reactions of *para*-haloanisoles afford dimers **6q**–**6t** in 20–37% yields. In these cases, full consumption of the reactants was observed, and the lower isolated yields are attributable to undesirable oxidation pathways involving the halogens. Substrates with an ester tethered or directly attached to the arene are accommodated and generate **6u** and **6v** in 64% and 52% yields, respectively.

**Scheme 2:**
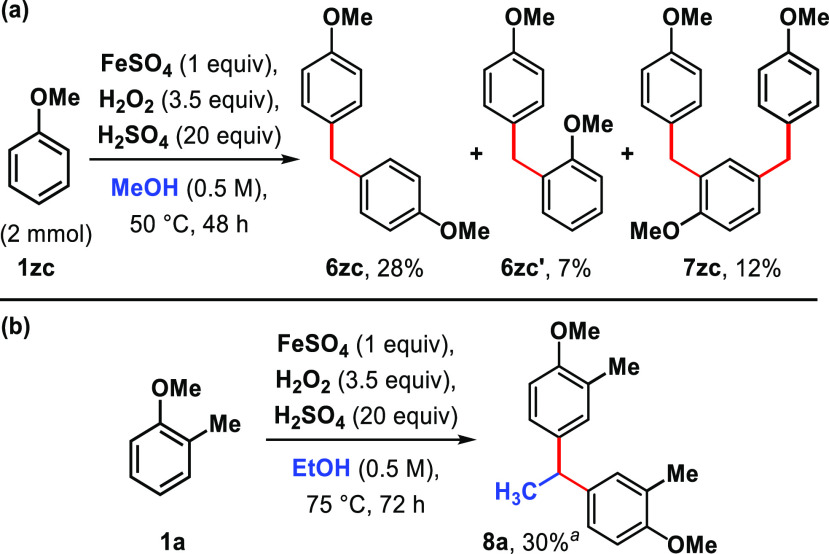
(a) Oxidative Alkylation of Unsubstituted Anisole; (b) Oxidative Alkylation with Ethanol Yields determined by NMR analysis of the crude reaction mixture with acetophenone as the internal standard. FeSO_4_·7H_2_O used.

We find that dimethoxynaphthalene derivatives are operated upon and transformed into dinaphthylmethanes **6w** and **6x** in 36% and 59% yields. In the latter case, the trimerized product was isolated in a 20% yield (see SI). Substituting the methyl group in anisole for other alkyl substituents such as ethyl or bromopropyl groups still permits the oxidative alkylations: 3-*tert*-Butylethoxybenzene is converted to diarylmethane **6y** in 63% yield, and bromopropoxybenzene, to **6z** in 47% yield. Diphenylether is similarly transformed into diarylmethane **6za** in 46% yield. 1,3,5-Dimethoxybenzene is converted to diarylmethane **6zb** in 61% yield. Free phenol is unreactive, potentially due to complexation with Fe(II) and unfavorably affecting the Fenton process (not shown). In general, substituted anisole derivatives are sterically biased to select a major product. Subjecting unsubstituted anisole (**1zc**) to the oxidative alkylation conditions yielded a mixture of both the symmetric *para*-/*para*-alkylated (**6zc**) in 28% yield, the unsymmetric *para*-/*ortho*-alkylated (**6zc′**) in 7% yield, and the trimer (**7zc**) in 12% yield (Scheme 2a). Under the reaction conditions, ethanol can serve as an alkylating agent, transforming 2-methylanisole (**1a**) into diarylethane **8a** in 30% yield (Scheme 2b).

**Scheme 3:**
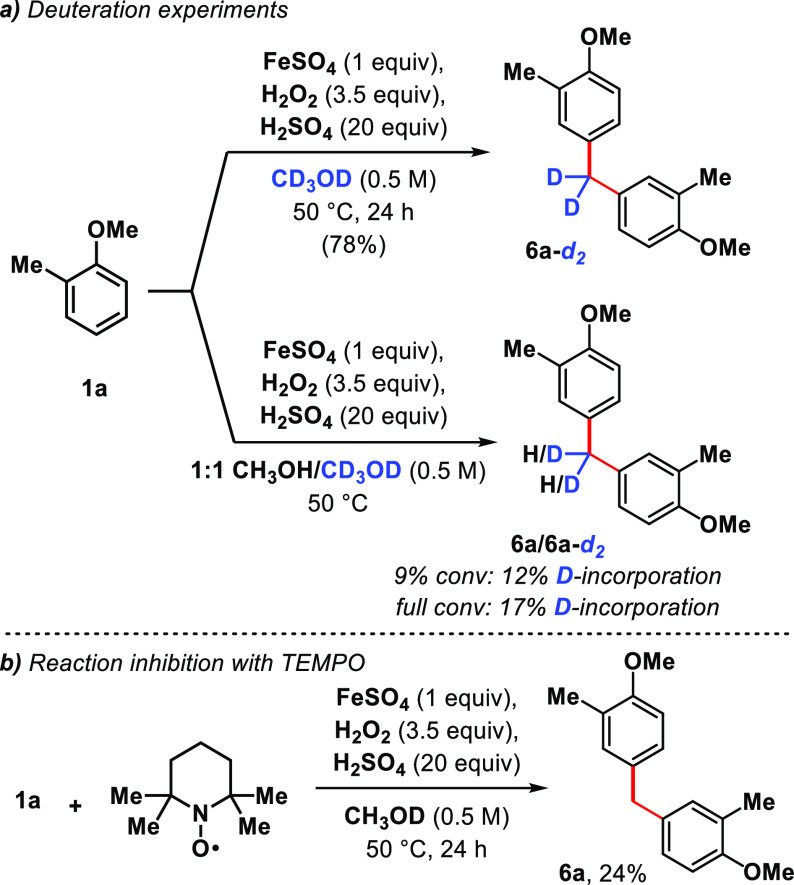
Mechanistic Experiments TEMPO = 2,2,6,6-tetramethyl-1-pipepridinyloxy. FeSO_4_·7H_2_O used.

The reaction proceeds in CD_3_OD to produce diarylmethane **6a**-***d*_*2*_** dideuterated at the benzylic position in good yield (78%). In competition studies using a 1:1 mixture of CH_3_OH/CD_3_OD, partial deuterations (12−17%) were observed at both low and full conversion of the starting material (Scheme 3a). The substantive isotopic effect (KIE ≈ 7) is indicative of a slow C–H bond breakage followed by a fast Friedel–Crafts alkylation, which is consistent with how the postulated benzylic alcohol intermediates (i.e., **5**) were never observed in any of the crude reaction mixtures. Addition of TEMPO (1 equiv) suppresses the reactivity, resulting in 24% conversion to the product (Scheme 3b, see SI).

**Figure 1:**
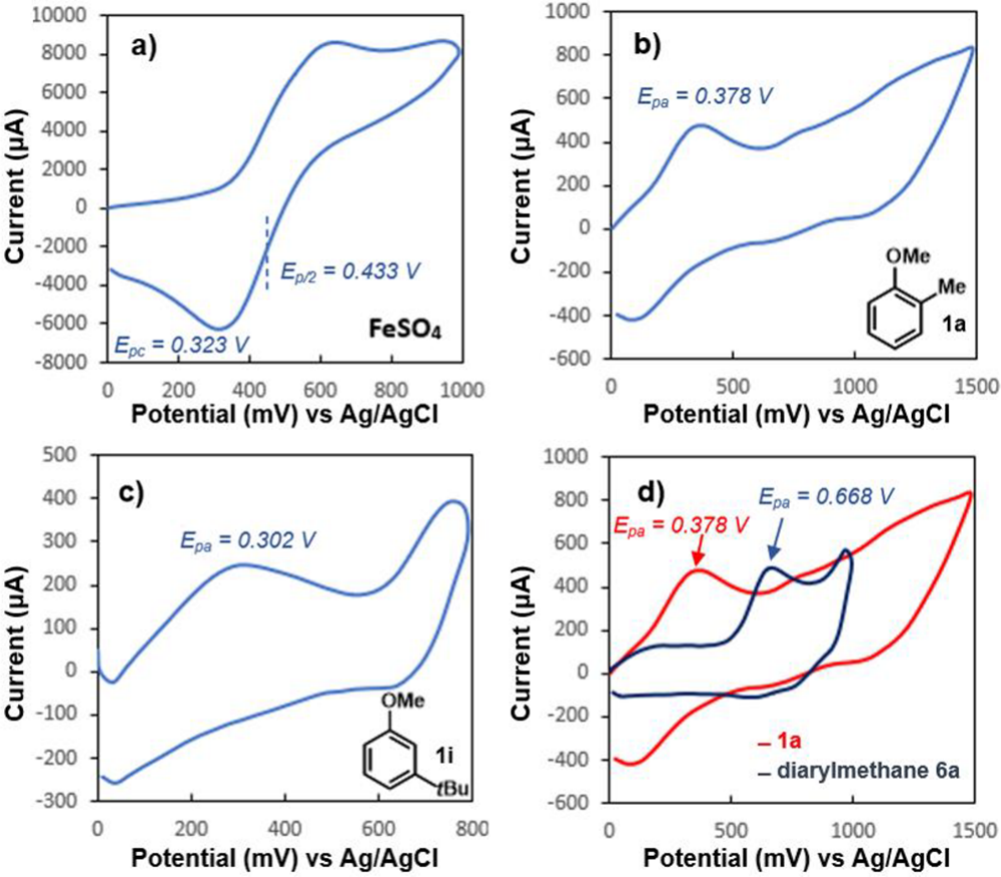
Electrochemical Measurements of (a) FeSO_4_ (in 1 M H_2_SO_4_). (b) **1a** in MeCN. (c) **1i** in MeCN. (d) **6a** in MeCN.

Cyclic voltammetry was employed to probe the likelihood of electron-transfer occurrences between iron and the anisole derivatives. The Fe(III) to Fe(II) reductive potential of iron sulfate (dissolved in acidic 1 M H_2_SO_4_ for solubility) was found to be 0.323 V with a half peak potential, *E*_p/2_ = 0.433 V (Figure 1a), and is similar to that obtained in neutral aqueous solution.^26^ The oxidative potentials of 2-methylanisole (**1a**) and 3-*tert*-butylanisole in MeCN were measured to be 0.378 and 0.302 V, respectively (Figure 1b and 1c).

The reductive potential of iron sulfate compared to the oxidative potentials of these arenes is supportive of Fe(III)-mediated oxidation of the anisole derivative to generate the putative radical cation (**3**). Cyclic voltammetry experiments with 4-fluoroanisole, 4-bromoanisole, and anisole recorded oxidative potentials of 1.07, 0.976, and 0.919 V, respectively (see SI).^27^ As expected, the oxidative potentials increased with decreasing electron densities in the arene. The increase in the oxidation potentials surpassing the reductive potential of Fe(III) is consistent with the drop in the reactivity and yields observed for these substrates. We also subjected the product diarylmethane (**6a**) to cyclic voltammetry measurements and found that its oxidative potential increased from 0.378 V (for precursor **1a**) to 0.668 V, which is consistent with the lack of subsequent oxidation events in most cases (Figure 1d). In general, CV sweeps of the arenes do not give rise to reversible waveforms, presumably due to oligomerization pathways on the surface of the anode.

**Figure 2:**
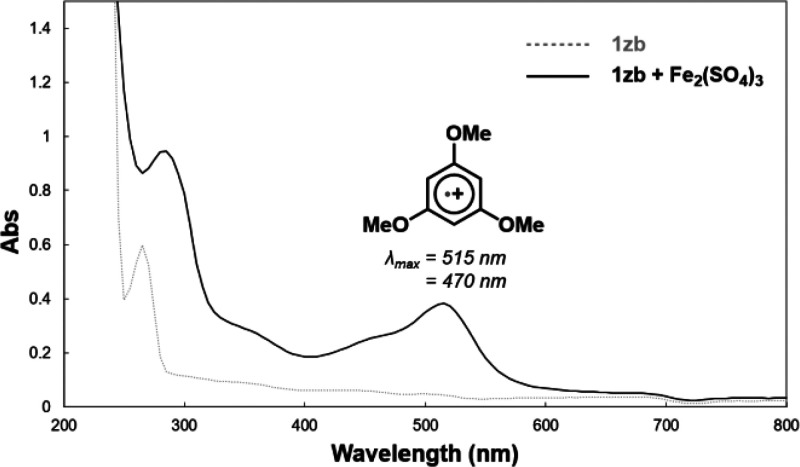
UV–vis absorbance spectra.

We turned to UV/vis spectroscopy to probe for radical cation formation under our reaction conditions with Fe_2_(SO_4_)_3_, and oxidant. Ishihara and co-workers reported an aromatic radical cation derived from FeCl_3_ and a sterically encumbered, fully substituted dimethoxyarene that persisted long enough for characterization.^20a^ Consistent with their observations, aliquots of our reaction mixtures involving relatively simple 2-methylanisole (**1a**) and 1,3-dimethoxybenzene (**1l**) did not yield UV/vis absorbance spectra that differed significantly from the reactants (see SI). Any radical cation intermediates formed were likely too short-lived to be measurable. The absence of distinct radical cation behavior would not necessarily preclude reactivity, as simple complexation of the arene with iron(III) could impart partial radical character that facilitates reactivity. However, in the reaction with 1,3,5-trimethoxybenzene (**1za**), distinct λ_max_’s at 515 and 470 nm were recorded in the UV/vis spectrum, consistent with its radical cation (Figure 2). This observation suggests that radical cations can arise from sufficiently electron-rich arenes and need not be fully substituted nor sterically encumbered, although the latter will enhance their persistence.

**Scheme 4:**
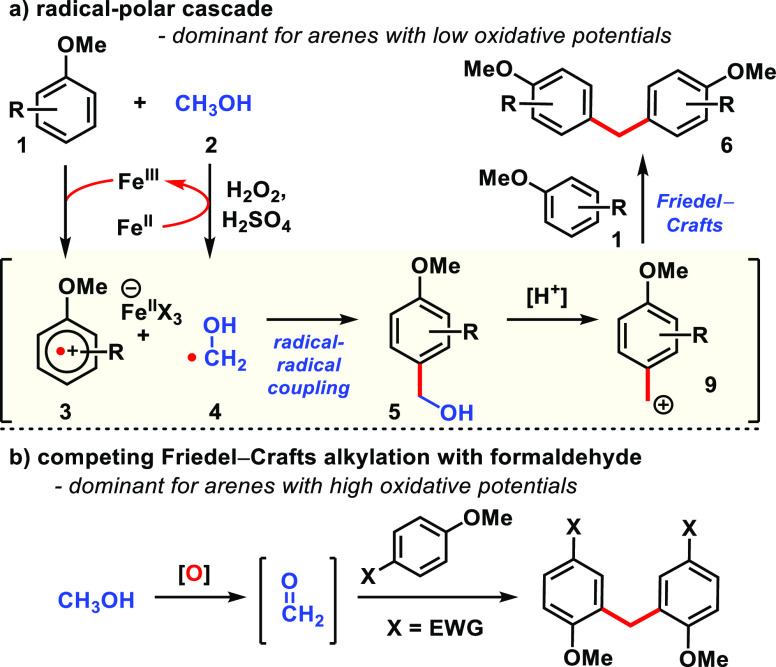
Proposed Radical-Polar Cascade Mechanism

Based on the mechanistic studies, we propose that both a radical-polar cascade involving hydroxymethyl radical as well as arene radical cation intermediates and the conventional Friedel–Crafts pathway involving formaldehyde are operative. The radical-polar cascade is initiated by methanol (**2**) reacting with the hydroxyl radical (HO·) produced from the Fe(II)-mediated Fenton decomposition of hydrogen peroxide to generate hydroxymethyl radical **4** (Scheme 4a). The Fe(III) resulting from this process complexes with anisole to form radical cation **3**, which could capture the nucleophilic radical (**4**) to produce benzylic alcohol **5**. A similar pathway has been proposed for the Fenton-mediated formylation of electron-poor quinoline and quinoxaline derivatives.^16b^ This pathway is favored for electron-rich arenes with low oxidative potentials. Under the strongly acidic condition in polar protic solvent, ionization to benzylic carbocation **9** is proposed and subsequent reaction with a second equivalent of anisole furnishes the observed diarylmethane **6**. A rationale for the reaction not being catalytic in iron at this stage is that much of it remains associated with the radical cation as the counteranion (i.e., intermediate **3**),^20a^ a phenomenon important for its persistence and enhancing its likelihood to participate in the proposed radical–radical coupling event. Oxidation of methanol to formaldehyde in situ, followed by conventional Friedel–Crafts alkylation, is possible and likely competitive, especially for arenes with high oxidative potentials (i.e., 4-haloanisoles, Scheme 4b). Methodologies that employ formaldehyde^28^ or trioxane^29^ to achieve diarylmethane synthesis require upward of 25-fold excess arene reagent with respect to formaldehyde and high reaction temperatures, but can be addressed by Brønsted acid-promoted Brønsted acid catalysis.^30^ Non-anisolic arenes such as *para*-xylene, mesitylene, toluene, and durene are unreactive under our reaction conditions despite their previously reported success in electrophilic aromatic substitution with formaldehyde,^28^ and the difference in scope is suggestive of a different mechanism at play. We ruled out a direct Friedel–Crafts methylation with protonated methanol because: (1) the resulting benzylic anisole derivatives were not observed as side products, and (2) several benzylanisole derivatives are transformed into diarylmethane products with the benzylic positions intact (e.g., Table 2, **6a**, **6b**, **6f**, **6h**, **6m**, and **6n**). The necessity of the electron-rich aryl ether motifs is consistent with the generation of radical cation intermediates that proceeds in reacting with hydroxymethyl radical **4**, thereby demonstrating proof-of-concept in mimicking nature to merge electron-rich radical intermediates with electron-rich arenes.

## Conclusion

Iron is a dual-role reagent in this methodology.^13a^ By implementing iron(II)-mediated decomposition of hydrogen peroxide and the Fe(III)-mediated oxidation of anisolic derivatives in tandem,^31^ an oxidative C(sp^2^)–C(sp^3^) bond formation was achieved wherein an electron-rich radical intermediate merges with electron-rich arenes. Further work on establishing catalytic and cross-coupling activities is underway in our laboratory.

## Data Availability

The data underlying this study are available in the published article and its Supporting Information.

## Supporting Information Available

General information, cyclic voltammetry studies, UV–vis spectroscopy, NMR spectra. (PDF)
FAIR data, including the primary NMR FID files, for compounds **6a**–**6zc**, **7zc**, **8a** (ZIP)

## References

[ref1] Wardman P.; Candeias L. P. Fenton chemistry: an introduction. Radiat. Res. 1996, 145, 523–531. 10.2307/3579270. 8619017

[ref2] aKepp K. P. Bioinorganic Chemistry of Alzheimer’s Disease. Chem. Rev. 2012, 112, 5193–5239. 10.1021/cr300009x. 22793492

[ref3] Sawicki K. T.; Chang H.-C.; Ardehali H. Role of Heme in Cardiovascular Physiology and Disease. J. Am. Heart Assoc. 2015, 4 No. (1), . 10.1161/JAHA.114.001138. PMC4330050 25559010

[ref4] Shigenaga M. K.; Hagen T. M.; Ames B. N. Oxidative damage and mitochondrial decay in aging. Proc. Natl. Acad. Sci. U.S.A. 1994, 91, 10771–10778. 10.1073/pnas.91.23.10771. 7971961 PMC45108

[ref5] Torti S. V.; Torti F. M. Iron and cancer: more ore to be mined. Nat. Rev. Cancer 2013, 13, 342–355. 10.1038/nrc3495. 23594855 PMC4036554

[ref6] aBabuponnusami A.; Muthukumar K. A review on Fenton and improvements to the Fenton process for wastewater treatment. J. Environ. Chem. Eng. 2014, 2, 557–572. 10.1016/j.jece.2013.10.011.

[ref7] aKim C.; Chen K.; Kim J.; Que L. Stereospecific Alkane Hydroxylation with H_2_O_2_ Catalyzed by an Iron(II)-Tris (2-pyridylmethyl) amine Complex. J. Am. Chem. Soc. 1997, 119, 5964–5965. 10.1021/ja9642572.

[ref8] Groendyke G. J.; Modak A.; Cook S. P. Fenton-Inspired C–H functionalization: Peroxide-Directed C–H Thioetherification. J. Org. Chem. 2019, 84, 13073–13091. 10.1021/acs.joc.9b01979. 31524395 PMC7423317

[ref9] Guan H.; Sun S.; Mao Y.; Chen L.; Lu R.; Huang J.; Liu L. Iron(II)-Catalyzed Site-Selective Functionalization of Unactivated C(sp^3^)–H Bonds Guided by Alkoxyl Radicals. Angew. Chem., Int. Ed. 2018, 57, 11413–11417. 10.1002/anie.201806434. 30016576

[ref10] Rivière C.; Pawlus A. D.; Mérillon J.-M. Natural stilbenoids: distribution in the plant kingdom and chemotaxonomic interest in Vitaceae. Nat. Prod. Rep. 2012, 29, 1317–1333. 10.1039/c2np20049j. 23014926

[ref11] Boerjan W.; Ralph J.; Baucher M. Lignin biosynthesis. Annu. Rev. Plant. Biol. 2003, 54, 519–546. 10.1146/annurev.arplant.54.031902.134938. 14503002

[ref12] Sydor P. K.; Barry S. M.; Odulate O. M.; Barona-Gomez F.; Haynes S. W.; Corre C.; Song L.; Challis G. L. Regio- and stereodivergent antibiotic oxidative carbocyclizations catalysed by Rieske oxygenase-like enzymes. Nat. Chem. 2011, 3, 388–392. 10.1038/nchem.1024. 21505498 PMC3085852

[ref13] aPan A.; Chojnacka M.; Crowley R. III; Göttemann L.; Haines B. E.; Kou K. G. M. Synergistic Brønsted/Lewis acid catalyzed aromatic alkylation with unactivated tertiary alcohols or di-*tert*-butylperoxide to synthesize quaternary carbon centers. Chem. Sci. 2022, 13, 3539–3548. 10.1039/D1SC06422C. 35432882 PMC8943850

[ref14] Smith J. M.; Harwood S. J.; Baran P. S. Radical Retrosynthesis. Acc. Chem. Res. 2018, 51, 1807–1817. 10.1021/acs.accounts.8b00209. 30070821 PMC6349421

[ref15] aDuncton M. A. J. Minisci reactions: Versatile CH-functionalizations for medicinal chemists. Med. Chem. Commun. 2011, 2, 1135–1161. 10.1039/c1md00134e.

[ref16] aColgan A. C.; Proctor R. S. J.; Gibson D. C.; Chuentragool P.; Lahdenperä A. S. K.; Ermanis K.; Phipps R. J. Hydrogen Atom Transfer Driven Enantioselective Minisci Reaction of Alcohols. Angew. Chem., Int. Ed. 2022, 61, e202200266 10.1002/anie.202200266. PMC9321721 35420220

[ref17] aPitre S. P.; Muuronen M.; Fishman D. A.; Overman L. E. Tertiary Alcohols as Radical Precursors for the Introduction of Tertiary Substituents into Heteroarenes. ACS Catal. 2019, 9, 3413–3418. 10.1021/acscatal.9b00405.

[ref18] aHofmann J.; Gans E.; Clark T.; Heinrich M. R. Radical Arylation of Anilines and Pyrroles via Aryldiazotates. Chem.—Eur. J. 2017, 23, 9647–9656. 10.1002/chem.201701429. 28440884

[ref19] aKarmakar U.; Hwang H. S.; Lee Y.; Cho E. J. Photocatalytic *para*-Selective C–H Functionalization of Anilines with Diazomalonates. Org. Lett. 2022, 24, 6137–6141. 10.1021/acs.orglett.2c02228. 35973228

[ref20] aHoribe T.; Ohmura S.; Ishihara K. Structure and Reactivity of Aromatic Radical Cations Generated by FeCl_3_. J. Am. Chem. Soc. 2019, 141, 1877–1881. 10.1021/jacs.8b12827. 30674190

[ref21] aShalit H.; Libman A.; Pappo D. *meso*-Tetraphenylporphyrin Iron Chloride Catalyzed Selective Oxidative Cross-Coupling of Phenols. J. Am. Chem. Soc. 2017, 139, 13404–13413. 10.1021/jacs.7b05898. 28862442

[ref22] aHuang Z.; Jin L.; Feng Y.; Peng P.; Yi H.; Lei A. Iron-Catalyzed Oxidative Radical Cross-Coupling/Cyclization between Phenols and Olefins. Angew. Chem., Int. Ed. 2013, 52, 7151–7155. 10.1002/anie.201210023. 23733624

[ref23] Schmidt H. G. Safe Piranhas: A Review of Methods and Protocols. ACS. Chem. Health Saf. 2022, 29, 54–61. 10.1021/acs.chas.1c00094.

[ref24] aPallikonda G.; Chakravarty M. Benzylic Phosphates in Friedel–Crafts Reactions with Activated and Unactivated Arenes: Access to Polyarylated Alkanes. J. Org. Chem. 2016, 81, 2135–2142. 10.1021/acs.joc.5b02441. 26835977

[ref25] Chen H.-Y. Why the Reactive Oxygen Species of the Fenton Reaction Switches from Oxoiron(IV) Species to Hydroxyl Radical in Phosphate Buffer Solutions? A Computational Rationale. ACS Omega 2019, 4, 14105–14113. 10.1021/acsomega.9b02023. 31497730 PMC6714542

[ref26] Nagasaka M.; Yuzawa H.; Horigome T.; Hitchcock A. P.; Kosugi N. Electrochemical Reaction of Aqueous Iron Sulfate Solutions Studied by Fe L-Edge Soft X-ray Absorption Spectroscopy. J. Phys. Chem. C 2013, 117, 16343–16348. 10.1021/jp405112r.

[ref27] Jaworski J. S.; Cembor M.; Orlik M. Anisole as a solvent for organic electrochemistry. J. Electroanal. Chem. 2005, 582, 165–170. 10.1016/j.jelechem.2005.01.003.

[ref28] Li Z. X.; Duan Z.; Wu Y. J. FeCl_3_ catalyzed diarylmethanes formation. Chin. Chem. Lett. 2009, 20, 511–513. 10.1016/j.cclet.2009.01.033.

[ref29] Sun H.-B.; Hua R.; Yin Y. An efficient synthesis of diarylmethanes via InCl_3_•4H_2_O-catalyzed dehydration of electron-rich arenes with trioxane. Tetrahedron Lett. 2006, 47, 2291–2294. 10.1016/j.tetlet.2006.02.020.

[ref30] aZhang S.; Vayer M.; Noel F.; Vukovic V. D.; Golushko A.; Rezajooei N.; Rowley C. N.; Lebœuf D.; Moran J. Unlocking the Friedel-Crafts arylation of primary aliphatic alcohols and epoxides driven by hexafluoroisopropanol. Chem. 2021, 7, 3425–3441. 10.1016/j.chempr.2021.10.023.

[ref31] aBian Q.; Wu C.; Yuan J.; Shi Z.; Ding T.; Huang Y.; Xu H.; Xu Y. Iron Nitrate-Mediated Selective Synthesis of 3-Acyl-1,2,4-oxadiazoles from Alkynes and Nitriles: The Dual Roles of Iron Nitrate. J. Org. Chem. 2020, 85, 4058–4066. 10.1021/acs.joc.9b03070. 31994881

