# Supplementary material for: A Fenton Approach to Aromatic Radical Cations and Diarylmethane Synthesis
Source: J Org Chem. 2023 Oct 17;88(21):15060–6. doi: 10.1021/acs.joc.3c01505 (PMC10629232; doi:10.1021/acs.joc.3c01505)
Supplement: Supplementary file 1 — jo3c01505_si_001.pdf [file jo3c01505_si_001.pdf]

Supporting Information

A Fenton Approach to Aromatic Radical Cations and Diarylmethane Synthesis

Robert Crowley III, Berkley Lujan,† Alex Martinez,† Roni Manasi, Justin D. DeBow, and Kevin G. M. Kou*

Department of Chemistry, University of California, Riverside, California 92507, United States

* kevin.kou@ucr.edu

† Denotes equal contributions

Table of Contents

1. General Information 2

i) Solvents and reagents 2

ii) Reaction setup, progress monitoring, and product purification 3

2. Iron-Mediated Oxidative Arene Alkylation 4

i) General Procedure: Diarylmethane formation from methanol 4

ii) Product Characterization 4

3. Deuteration Study 18

4. TEMPO Trapping Experiment 19

5. Cyclic Voltammetry Studies 20

6. UV-Vis Spectroscopy 22

7. References 23

8. NMR Spectra 24

1. General Information

i) Solvents and reagents

Commercial reagents were purchased from MilliporeSigma, Acros Organics, Chem-Impex, TCI, Oakwood, and Alfa Aesar, and used without additional purification. Solvents were purchased from Fisher Scientific, Acros Organics, Alfa Aesar, and MilliporeSigma. Tetrahydrofuran (THF), diethyl ether (Et_2O), acetonitrile (MeCN), dichloromethane (CH_2Cl_2), benzene, 1,4-dioxane, and triethylamine (Et_3N) were sparged with argon and dried by passing through alumina columns using argon in a Glass Contour (Pure Process Technology) solvent purification system. Dimethylformamide (DMF), dimethyl sulfoxide (DMSO), and dichloroethane (DCE) were purchased in Sure/Seal or AcroSeal bottling and dispensed under N_2. Deuterated solvents were obtained from Cambridge Isotope Laboratories, Inc. or MilliporeSigma. The following anisolic reactants are commercially available and were used directly without purification.

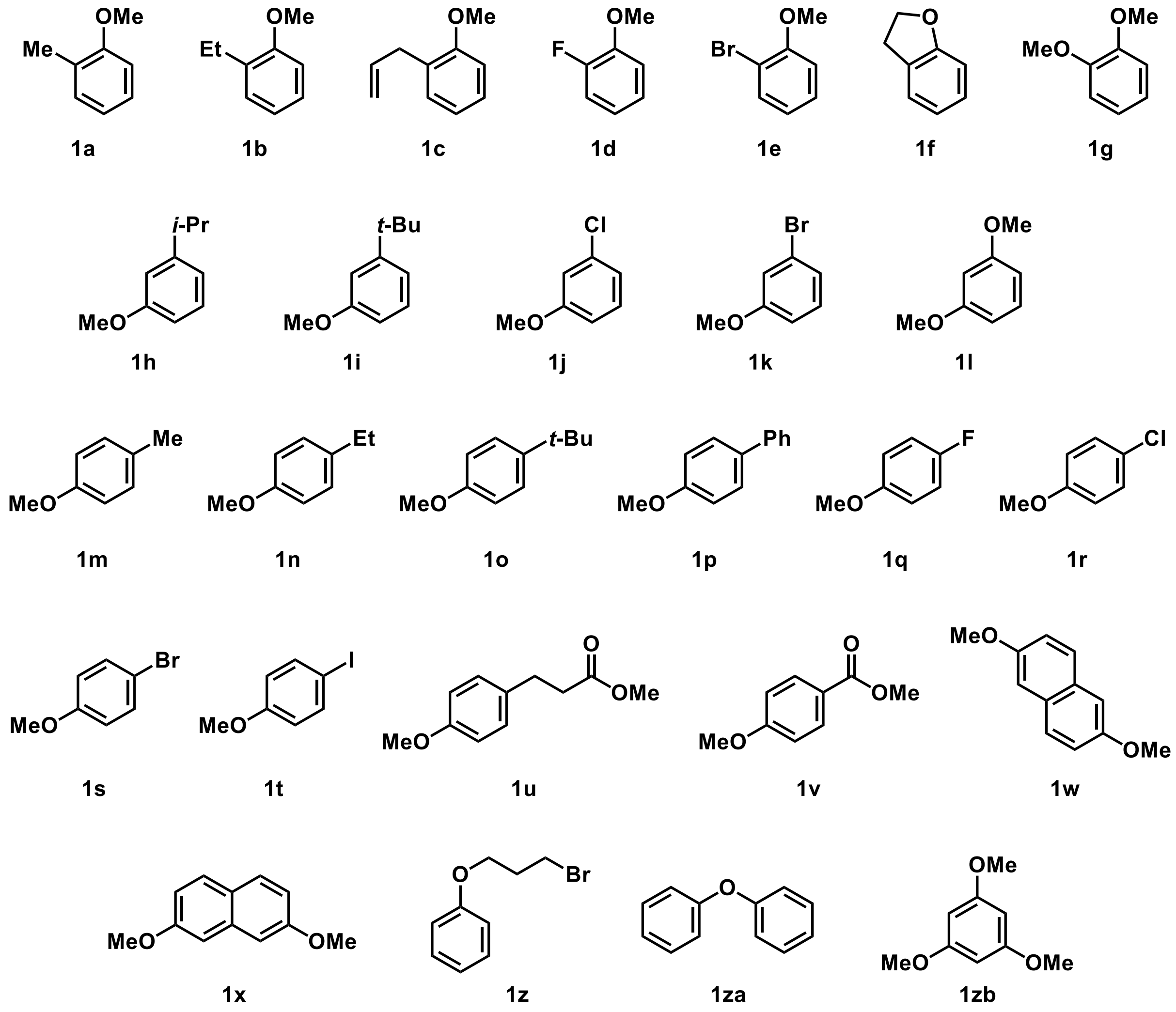

Table S1. Commercially available anisolic reactants used in this study.

Aryl ethers **1x** and **1y** were prepared using our previously published procedure.[1]

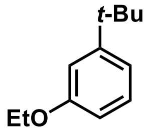

1x

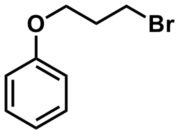

1y

ii) Reaction setup, progress monitoring, and product purification

All reagents, including the solvent, were added under ambient conditions open to air. The reactions were heated on a heating block that rests directly over top of the heating plate (0.2 mmol scale) or by submerging in an oil bath (>0.5 mmol scale). Reaction progresses were monitored using thin-layer chromatography (TLC) on EMD Silica Gel 60 F254 or Macherey–Nagel SIL HD (60 Å mean pore size, 0.75 mL/g specific pore volume, 5–17 μm particle size, with fluorescent indicator) silica gel plates. Visualization of the developed plates was performed under UV light (254 nm). Purification and isolation of products were performed via silica gel chromatography (both column and preparative thin-layer chromatography). Organic solutions were concentrated under reduced pressure on IKA® temperature-controlled rotary evaporator equipped with an ethylene glycol/water condenser.

iii) *Analytical instrumentation*

Melting points were taken using the BÜCHI B-545 melting point apparatus.

Proton nuclear magnetic resonance (^{1}H NMR) spectra, carbon nuclear magnetic resonance (^{13}C{^{1}H} NMR) spectra and fluorine nuclear magnetic resonance (^{19}F NMR) spectra were recorded on Bruker Avance NEO 400 (not ^{1}H decoupled) or Bruker Avance 600 MHz spectrometers (^{1}H decoupled). Chemical shifts (δ) are reported in ppm relative to the residual solvent signal (δ 7.26 for ^{1}H NMR, δ 77.16 for ^{13}C{^{1}H} NMR in $CDCl_3$).[1] Data for ^{1}H NMR spectroscopy are reported as follows: chemical shift (δ ppm), multiplicity (s = singlet, d = doublet, t = triplet, q = quartet, m = multiplet, br = broad, dd = doublet of doublets, dt = doublet of triplets), coupling constant (Hz), integration. Data for ^{13}C and ^{19}F NMR spectroscopy are reported in terms of chemical shift (δ ppm).

IR spectroscopic data were recorded on a NICOLET 6700 FT-IR spectrophotometer using a diamond attenuated total reflectance (ATR) accessory. Samples are loaded onto the diamond surface either neat or as a solution in organic solvent and the data acquired after the solvent had evaporated.

High resolution accurate mass (ESI) spectral data were obtained from the Analytical Chemistry Instrumentation Facility at the University of California, Riverside, on an Agilent 6545 Q-TOF LC/MS instrument (supported by NSF grant CHE-1828782). High resolution accurate mass (EI) spectral data were

obtained from the Mass Spectrometry Facility at the University of California, Irvine, on a ThermoFinnegan TraceMS+ GC EI/CI instrument.

UV/vis spectra were recorded on an Agilent Cary 60 UV/Vis spectrophotometer (190–1100 nm wavelength range with 1.5 nm resolution).

Cyclic voltammograms were recorded on an IKA Electrasyn 2.0 at room temperature.

2. Iron-Mediated Oxidative Arene Alkylation

i) General Procedure: Diarylmethane formation from methanol

In a 1-dram vial, iron sulfate heptahydrate (55.6 mg, 0.2 mmol, 1 equiv) was suspended in methanol (0.4 mL, 0.5 M). The solution was chilled to 0 ºC in an ice-water bath before addition of H_2SO_4 (217 µL, 4.0 mmol, 20 equiv) and 30% H_2O_2 (75 µL, 0.7 mmol, 3.5 equiv). The resultant solution was warmed to rt and stirred for 15 min before arene addition (0.2 mmol, 1 equiv). The reaction mixture was heated at 50–75 °C for 18–72 h on a heating block, at which time the solution was diluted with 15% $NaOH_{(aq)}$ solution (0.5 mL) and water (3 mL), then extracted with CH_2Cl_2 (3 x 5 mL). The combined organic extract was washed with brine (5 mL) and dried over Na_2SO_4. The solution was concentrated *in vacuo* and purified via silica gel chromatography to obtain the alkylation product.

Safety Note. Sulfuric acid and hydrogen peroxide form highly oxidizing mixtures. An exothermic reaction occurs when the two reagents are mixed neat. It is important to first cool the methanolic solution in an ice-water bath and ensure that the mixture is cold and rapidly stirring before dropwise adding the hydrogen peroxide solution. Any exotherm/effervescence is hard to notice on small scale. The aqueous waste obtained after liquid-liquid extraction should be neutralized using a saturated solution of sodium bisulfite.

ii) Product Characterization

3, 3'-Dimethyl-4, 4'-dimethoxydiphenylmethane (6a)

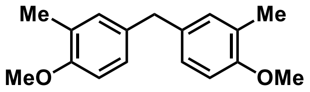

Prepared via the general procedure with 2-methylanisole (25 µL, 0.20 mmol, 1 equiv), reacting at 50 °C for 18 h. Purification by flash chromatography (eluting with 19:1 hexanes/EtOAc) afforded **6a** (0.0215 g, 84%) as a clear oil. R_f: 0.38 (19:1 hexanes/EtOAc). ^1H NMR ($CDCl_3$, 600 MHz) δ 6.96 (m, 4H), 6.74 (d, *J* = 8.0 Hz, 2H), 3.80 (s, 8H), 2.18 (s, 6H); $^{13}C\{^1H\}$ NMR ($CDCl_3$, 126 MHz) δ 156.2, 133.6, 131.3, 126.9, 126.6, 110.0, 55.5, 40.3, 16.4. IR (ATR): 3030, 2905, 2833, 1610, 1500, 1463, 1440, 1377, 1294, 1219, 1182, 1060, 917, 888, 750, 581, 564 cm^{-1}. HRMS (ESI+): *m/z* $[M+H]^+$ calculated for $[C_{17}H_{21}O_2]^+$: 257.1536; found: 257.1535. The spectral data is consistent with those previously reported in the literature.[2]

Scale-up. A 25 mL round bottom flask was charged with iron sulfate heptahydrate (0.556 g, 2.00 mmol, 1 equiv) and methanol (4 mL, 0.5 M). The solution was chilled to 0 ºC before addition of H_2SO_4 (2.17 mL, 7 mmol, 20 equiv) and 30% $H_2O_{2(aq)}$ (0.75 mL, 7 mmol, 3.5 equiv). The resultant solution was warmed to rt and stirred for 30 min before addition of 2-methylanisole (0.25 mL, 2.0 mmol, 1 equiv). The reaction was heated at 50 ºC in an oil bath for 48 h, at which time it was diluted with 15% $NaOH_{(aq)}$ (5 mL) and water (25 mL), then extracted with CH_2Cl_2 (3 x 30 mL). The combined organic extract was washed with brine (30 mL) and dried over anhydrous Na_2SO_4. The solvent was removed *in vacuo* and purified via flash chromatography (eluting with 19:1 hexanes/EtOAc) to afford **6a** (0.2373 g, 86%) as a clear oil.

Safety Note. Sulfuric acid and hydrogen peroxide form highly oxidizing mixtures. An exothermic reaction occurs when the two reagents are mixed neat. It is important to first cool the methanolic solution in an ice-water bath and ensure that the mixture is cold (by leaving the vessel submerged in the ice bath for ~5 minutes) before dropwise adding *cold* hydrogen peroxide solution. If the hydrogen peroxide solution is added too quickly, or if the mixtures are not sufficiently cold, a potentially violent exotherm with heavy bubbling can occur. With slow addition to the stirring cold reaction mixture, effervescence is unnoticeable. The aqueous waste obtained after liquid-liquid extraction should be neutralized using a saturated solution of sodium bisulfite.

3, 3'-Diethyl-4, 4'-dimethoxydiphenylmethane (6b)

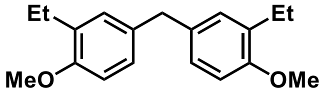

Prepared via the general procedure with 2-ethylanisole (30 µL, 0.20 mmol, 1 equiv), reacting at 50 °C on a heating block for 18 h. Purification by flash chromatography (eluting with 9:1 hexanes/EtOAc) afforded **6b** (0.0194 g, 68%) as a yellow oil. R_f: 0.48 (9:1 hexanes/EtOAc). ^{1}H NMR ($CDCl_3$, 600 MHz) δ 7.01 (s, 2H), 6.99 (d, *J* = 7.8 Hz, 2H), 6.80 (d, *J* = 8.4 Hz, 2H), 3.87 (s, 2H), 3.83 (s, 6H), 2.63 (q, *J* = 7.2 Hz, 4H), 1.20 (t, *J* = 7.8 Hz, 6H); ^{13}C{^{1}H} NMR ($CDCl_3$, 126 MHz) δ 156.0, 133.9, 132.8, 129.9, 127.1, 110.5, 55.7, 40.7, 23.7, 14.6. IR (ATR): 3072, 2961, 2931, 2833, 1609, 1498, 1462, 1288, 1242, 1181, 1134, 1033, 928, 884, 804, 746, 635, 576, 539, 528 cm^{-1}. HRMS (ESI+): *m/z* [M+H]$^+$ calculated for $[C_{19}H_{25}O_2]^+$: 285.1849; found: 285.1842.

bis(3-Allyl-4-methoxyphenyl)methane (6c)

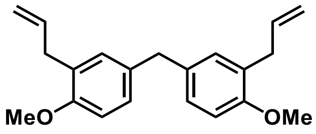

Prepared via the general procedure with 2-allylanisole (31 µL, 0.20 mmol, 1 equiv), reacting at 50 °C on a heating block for 18 h. Purification by flash chromatography (eluting with 19:1 hexanes/EtOAc) afforded **6c** (0.0112 g,

36%) as a clear oil. R_f: 0.4 (19:1 hexanes/EtOAc). ^{1}H NMR (600 MHz, $CDCl_3$) δ 6.98 (s, 4H), 6.78 (d, *J* = 8.8 Hz, 2H), 5.99 (td, *J* = 16.6, 6.6 Hz, 2H), 5.04 (d, *J* = 17.1 Hz, 4H), 3.84 (s, 2H), 3.80 (s, 6H), 3.36 (d, *J* = 6.2 Hz, 4H); ^{13}C{^{1}H} NMR ($CDCl_3$, 126 MHz): δ 155.7, 137.2, 133.7, 130.5, 128.6, 127.5, 115.4, 110.5, 55.6, 40.4, 34.4. IR (ATR): 3096, 3001, 2905, 2833, 2360, 2159, 2025, 1638, 1609, 1498, 1463, 1439, 1330, 1246, 1223, 1182, 908, 805, 779, 732, 657, 620, 559, 539, 528 cm^{-1}. HRMS (ESI+): *m/z* $[M+H]^+$ calculated for $[C_{21}H_{25}O_2]^+$: 309.1849; found: 309.1852.

bis(3-Fluoro-4-methoxyphenyl)methane (6d)

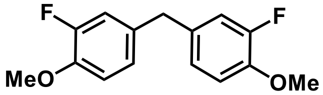

Prepared via the general procedure with 2-fluoroanisole (22 µL, 0.20 mmol, 1 equiv), reacting at 75 °C on a heating block for 48 h. Purification by flash chromatography (eluting with 19:1 hexanes/EtOAc) afforded **6d** (0.0077 g, 29%) as a colorless oil. R_f: 0.48 (19:1 hexanes/EtOAc). ^{1}H NMR ($CDCl_3$, 600 MHz): δ 6.87 (m, 6H), 3.86 (s, 6H), 3.82 (s, 2H); ^{13}C{^{1}H} NMR ($CDCl_3$, 126 MHz) δ 152.3 (d, *J* = 201.6 Hz), 146.0 (d, *J* = 8.8 Hz), 133.9 (d, *J* = 4.7 Hz), 124.3, 116.5 (d, *J* = 15.2 Hz), 113.5, 56.4, 40.0; ^{19}F NMR ($CDCl_3$, 564 MHz): δ 135.2 (dd, *J* = 10.8, 7.8 Hz). IR (ATR): 3025, 3005, 2917, 2839, 1509, 1464, 1141, 1269, 1219, 1124, 1028, 966, 806, 761, 750, 669, 575, 536 cm^{-1}. HRMS (ESI+): *m/z* $[M+H]^+$ calculated for $[C_{15}H_{15}F_2O_2]^+$: 265.1035; found: 265.1019.

bis(3-Bromo-4-methoxyphenyl)methane (6e)

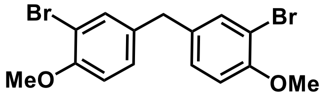

Prepared via the general procedure with 2-bromoanisole (25 µL, 0.20 mmol, 1 equiv), reacting at 75 °C on a heating block for 48 h. Purification by flash chromatography (eluting with 19:1 hexanes/EtOAc) afforded **6e** (0.0173 g, 45%) as a white solid. R_f: 0.33 (19:1 hexanes/EtOAc). M.p. 100–101 °C. ^{1}H NMR ($CDCl_3$, 600 MHz): δ 7.37 (d, *J* = 1.8 Hz, 2H), 7.09 (dd, *J* = 8.4, 2.4 Hz, 2H), 6.85 (d, *J* = 8.4 Hz, 2H), 3.90 (s, 6H), 3.84 (s, 2H); ^{13}C{^{1}H} NMR ($CDCl_3$, 126 MHz): δ 154.5, 134.6, 133.7, 128.8, 112.1, 111.8, 56.4, 39.5. IR (ATR): 3011, 2914, 2838, 2359, 1726, 1600, 1569, 1489, 1464, 1454, 1437, 1400, 1287, 1248, 1205, 1180, 1151, 1050, 1016, 898, 877, 812, 799, 754, 704, 674, 666, 621, 554 cm^{-1}. HRMS (ESI+): *m/z* $[M+2+Na]^+$ calculated for $[C_{15}H_{14}Br_2O_2Na]^+$: 408.9232; found: 408.9236. The spectral data is consistent with those previously reported in the literature.[3]

bis(2,3-Dihydrobenzofuran-5-yl)methane (6f)

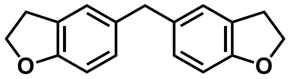

Prepared via the general procedure with 2,3-dihydrobenzofuran (23 µL, 0.20 mmol, 1 equiv), reacting at 50 °C on a heating block for 18 h. Purification by

flash chromatography (eluting with 19:1 hexanes/EtOAc) afforded **6f** (0.0185 g, 73%) as a yellow oil. R_f: 0.42 (19:1 hexanes/EtOAc). ^{1}H NMR ($CDCl_3$, 600 MHz): δ 6.99 (s, 2H), 6.92 (d, *J* = 8.4 Hz, 2H), 6.70 (d, *J* = 7.8 Hz, 2H), 4.53 (t, *J* = 8.4 Hz, 4H), 3.82 (s, 2H), 3.16 (t, *J* = 8.4 Hz, 4H); ^{13}C{^{1}H} NMR ($CDCl_3$, 126 MHz): δ 158.5, 134.0, 128.3, 127.3, 125.4, 109.1, 71.3, 40.9, 29.9. IR (ATR): 3013, 2914, 2896, 2854, 2360, 1607, 1486, 1439, 1367, 1290, 1247, 1227, 1197, 1101, 984, 952, 878, 819, 764, 710, 640, 584, 555, 538 cm^{-1}. HRMS (ESI+): *m/z* $[M+H]^+$ calculated for $[C_{17}H_{17}O_2]^+$: 253.1223; found: 253.1215.

bis(3,4-Dimethoxyphenyl)methane (6g)

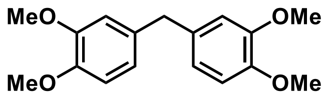

Prepared via the general procedure with 1,2-dimethoxybenzene (26 µL, 0.20 mmol, 1 equiv), reacting at 75 °C on a heating block for 72 h. Purification by flash chromatography (eluting with 17:3 hexanes/EtOAc) afforded **6g** (0.0052 g, 18%) as a clear oil. R_f: 0.10 (17:3 hexanes/EtOAc). ^{1}H NMR (500 MHz, $CDCl_3$) δ 6.80 (d, *J* = 8.1 Hz, 2H), 6.74–6.69 (m, 4H), 3.88 (s, 2H), 3.84 (s, 12H); ^{13}C{^{1}H} NMR ($CDCl_3$, 126 MHz): δ 148.9, 147.4, 133.9, 120.8, 112.1, 111.2, 55.9, 41.0. IR (ATR): 3043, 2934, 2834, 1589, 1463, 1417, 1338, 1255, 1230, 1192, 1137, 1026, 909, 859, 801, 726, 646, 600, 555, 544, 537 cm^{-1}. HRMS (ESI+): *m/z* $[M+NH_4]^+$ calculated for $[C_{17}H_{24}NO_4]^+$: 306.1700; found: 306.1684. The spectral data is consistent with those previously reported in the literature.[4]

bis(4-(Isopropyl)-2-methoxyphenyl)methane (6h)

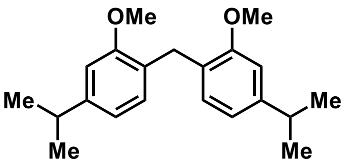

Prepared via the general procedure with 1-(isopropyl)-3-methoxybenzene (32 µL, 0.20 mmol, 1 equiv), reacting at 75 °C on a heating block for 18 h. Purification by flash chromatography (eluting with 5:1 hexanes/CH_2Cl_2) afforded **6h** (0.0188 g, 60%) as a colorless oil. R_f: 0.70 (5:1 hexanes/DCM). ^{1}H NMR ($CDCl_3$, 600 MHz) δ 6.87 (d, *J* = 2.4 Hz, 2H), 6.77 (d, *J* = 8.4 Hz, 2H), 6.63 (dd, *J* =8.4, 2.4 Hz, 2H), 3.93 (s, 2H), 3.80 (s, 6H), 3.09 (m, 2H), 1.20 (d, *J* = 6.6 Hz, 12H); ^{13}C{^{1}H} NMR ($CDCl_3$, 126 MHz) δ 158.5, 148.5, 130.7, 130.1, 111.7, 110.3, 55.3, 33.9, 29.2, 23.7. IR (ATR): 3012, 2959, 2868, 2833, 1609, 1576, 1490, 1463, 1383, 1363, 1285, 1235, 1205, 1069, 1036, 930, 871, 802, 668 cm^{-1}. HRMS (ESI+): *m/z* $[M+H]^+$ calculated for $[C_{21}H_{29}O_2]^+$: 313.2162; found: 313.2153.

bis(4-(*tert*-Butyl)-2-methoxyphenyl)methane (6i)

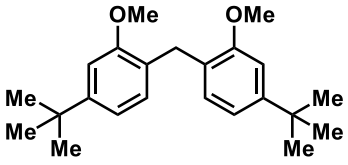

Prepared via the general procedure with 3-*tert*-buytlanisole (35 µL, 0.20 mmol, 1 equiv), reacting at 50 °C on a heating block for 72 h. Purification by flash chromatography (eluting with 17:3 hexanes/CH_2Cl_2) afforded **6i** (0.0180 g, 53%) as a clear oil. R_f: 0.42 (17:3 hexanes/CH_2Cl_2). ^{1}H NMR ($CDCl_3$, 500 MHz) δ 6.97 (d, J = 7.8 Hz, 2H), 6.89 (dd, J = 10.6, 2.8 Hz, 4H), 3.90 (s, 2H), 3.85 (s, 6H), 1.32 (s, 18H); ^{13}C{^{1}H} NMR ($CDCl_3$, 126 MHz) δ ^{13}C{^{1}H} NMR (151 MHz, $CDCl_3$) δ 157.4, 150.4, 130.0, 126.5, 117.3, 107.9, 55.5, 34.8, 31.6, 29.0. IR (ATR): 3015, 2956, 2359, 2114, 1601, 1559, 1541, 1413, 1465, 1362, 1234, 1193, 1027, 910, 811, 729, 673, 646, 601, 556, 536 cm^{-1}. HRMS (ESI+): *m/z* $[M+Na]^+$ calculated for $[C_{23}H_{32}O_2Na]^+$: 363.2295; found: 363.2287.

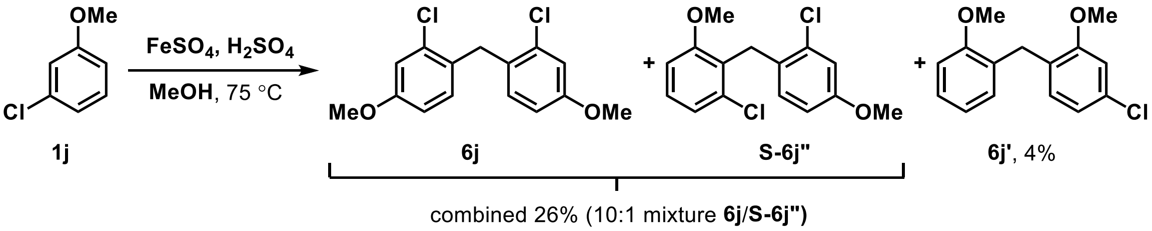

Prepared via the general procedure with 3-chloroanisole (24 µL, 0.20 mmol, 1 equiv), reacting at 75 °C on a heating block for 48 h. Purification by flash chromatography (eluting with hexanes, 5 elutions) afforded a 10:1 mixture of **6j/S-6j"** (0.0077 g, 26%) as a colorless oil with R_f: 0.11 (95:5 hexanes/EtOAc) and **6j'** (0.0012 g, 4%) as a clear oil with R_f: 0.38 (95:5 hexanes/EtOAc).

bis(2-(Chloro)-4-methoxyphenyl)methane (6j)

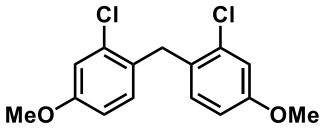

^{1}H NMR ($CDCl_3$, 600 MHz): δ 6.96 (d, J = 2.4 Hz, 2H), 6.93 (d, J = 8.4 Hz, 2H), 6.73 (dd, J = 8.4, 2.4 Hz, 2H), 4.05 (s, 2H), 3.78 (s, 6H); ^{13}C{^{1}H} NMR ($CDCl_3$, 126 MHz): δ 158.8, 134.8, 131.2, 129.6, 114.9, 113.1, 55.6, 35.2. IR (ATR): 2939, 2835, 1575, 1491, 1462, 1436, 1283, 1236, 1201, 1181, 1034, 912, 839, 801, 762, 691, 605, 551, 528 cm^{-1}. HRMS (ESI+): *m/z* $[M+Na]^+$ calculated for $[C_{15}H_{14}Cl_2O_2Na]^+$: 319.0263; found: 319.0255.

bis(4-(Chloro)-2-methoxyphenyl)methane (6j')

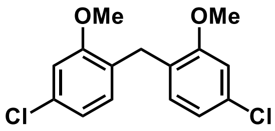

^{1}H NMR ($CDCl_3$, 600 MHz): δ 6.96 (d, J = 8.4 Hz, 2H), 6.83 (m, 4H), 3.83 (s, 2H), 3.80 (s, 6H); ^{13}C{^{1}H} NMR ($CDCl_3$, 126 MHz): δ 158.2, 132.6, 131.1, 127.3, 120.5, 111.1, 55.7, 29.3. IR (ATR): 3084, 2925, 2851, 1594, 1578, 1490, 1463, 1401, 1246, 1123, 1095, 1034, 914, 882, 836, 802, 781, 598, 539 cm^{-1}. HRMS (ESI+): *m/z* $[M+Na]^+$ calculated for $[C_{15}H_{14}Cl_2O_2Na]^+$: 319.0263; found: 319.0258.

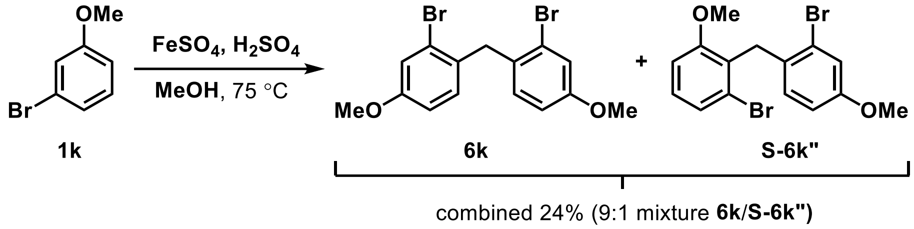

bis(2-(Bromo)-4-methoxyphenyl)methane (6k)

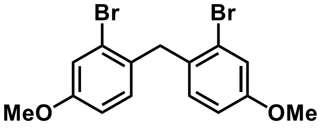

Prepared via the general procedure with 3-bromoanisole (25 µL, 0.20 mmol, 1 equiv), reacting at 75 °C on a heating block for 48 h. Purification by flash chromatography (eluting with hexanes, 5 elutions) afforded a 9:1 mixture of **6k/S-6k"** (0.0092 g, 24%) as a clear oil. R_f: 0.42 (95:5 hexanes/EtOAc). Major symmetric **6k**: ^{1}H NMR ($CDCl_3$, 600 MHz): δ 7.15 (d, *J* = 2.4 Hz, 2H), 6.89 (d, *J* = 8.4 Hz, 2H), 6.78 (dd, *J* = 8.4, 2.4 Hz, 2H), 4.06 (s, 2H), 3.79 (s, 6H); ^{13}C{^{1}H} NMR ($CDCl_3$, 126 MHz): δ 158.8, 131.4, 131.1, 125.1, 118.1, 113.7, 55.7, 40.4. IR (ATR): 3011, 2914, 2838, 2359, 1569, 1489, 1464, 1454, 1437, 1400, 1287, 1248, 1205, 1180, 1151, 1050, 1016, 898, 877, 812, 799, 754, 704, 674, 666, 621, 554 cm^{-1}. The spectroscopic data is consistent with those previously reported in the literature.[5]

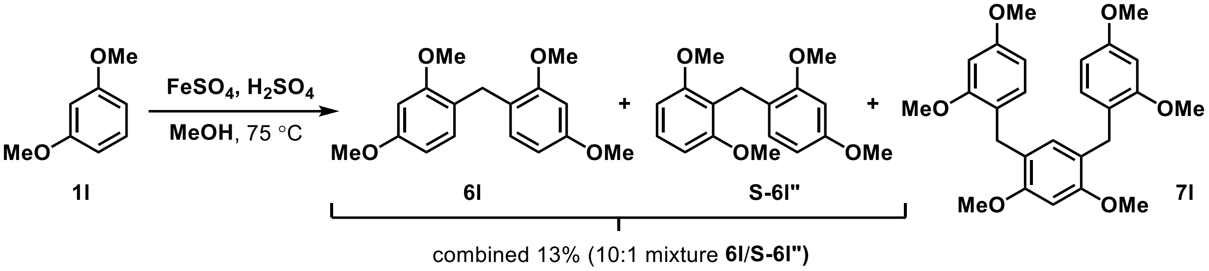

bis(2,4-Dimethoxyphenyl)methane (6l)

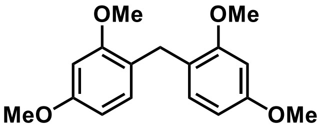

Prepared via the general procedure with 1,3-dimethoxybenzene (26 µL, 0.20 mmol, 1 equiv), reacting at 50 °C on a heating block for 18 h. Purification by flash chromatography (eluting with 4:1 hexanes/CH_2Cl_2) afforded a 10:1 mixture of **6l/S-6l"** (0.0037 g, 13%) as a colorless oil. R_f: 0.67 (4:1 hexanes/CH_2Cl_2). Major symmetric **6l**: ^{1}H NMR ($CDCl_3$, 600 MHz) δ 6.91 (d, *J* = 8.3 Hz, 2H), 6.49 (d, *J* = 2.4 Hz, 2H), 6.39 (dd, *J* = 8.3, 2.4 Hz, 2H), 3.80 (s, 6H), 3.79 (s, 6H) 3.75 (s, 2H); ^{13}C{^{1}H} NMR ($CDCl_3$,126 MHz) δ 159.1, 158.4, 130.4, 122.0, 103.8, 98.4, 55.5, 55.4, 28.5. IR (ATR): 3048, 2934, 2834, 2360, 1611, 1586, 1463, 1437, 1418, 1290, 1257, 1206, 1154 cm^{-1}. HRMS (ESI+): *m/z* $[M+H]^+$ calculated for $[C_{17}H_{21}O_4]^+$: 289.1434; found 289.1435.

4,4'-((4,6-dimethoxy-1,3-phenylene)bis(methylene))bis(1,3-dimethoxybenzene) (7l)

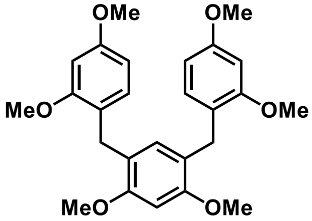

Prepared via the general procedure with 1,3-dimethoxybenzene (26 µL, 0.20 mmol, 1 equiv), reacting at 50 °C on a heating block for 18 h. Purification by flash chromatography (eluting with 9:1 CH_2Cl_2/hexanes) afforded a 5:1 mixture of symmetric/unsymmetric constitutional isomers (0.00554 g, 19%) as a colorless oil. R_f: 0.85 (9:1 CH_2Cl_2/hexanes). Major symmetric **7l**: ^{1}H NMR ($CDCl_3$, 400 MHz) δ 6.88 (d, *J* = 8.2 Hz, 2H), 6.70 (s, 1H), 6.46 (s, 1H), 6.40 (s, 2H), 6.35 (d, *J* = 8.1 Hz, 2H), 3.81 (s, 6H), 3.77 (s, 7H), 3.74 (s, 4H), 3.70 (s, 6H), 3.67 (s, 1H), 3.65 (s, 1H); ^{13}C{^{1}H} NMR ($CDCl_3$, 176 MHz) δ 159.0, 158.3, 156.5, 132.1, 130.3, 122.2, 120.7, 103.8, 98.3, 95.5, 55.8, 55.3, 55.3, 28.5; IR (ATR): 2996, 2880, 2440, 2287, 2050, 1855, 1463, 1416, 1207, 1178, 1109 cm^{-1}. HRMS (ESI+): *m/z* [M+**NH_4**]$^+$ calculated for $[C_{26}H_{34}NO_6]^+$: 456.2381; found 456.2367.

bis(2-methoxy-5-methylphenyl)methane (6m)

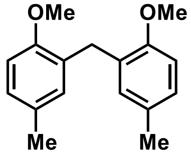

Prepared via the general procedure with 4-methylanisole (25 µL, 0.20 mmol, 1 equiv), reacting at 50 °C for 48 h. Purification by flash chromatography (eluting with 19:1 hexanes/EtOAc) afforded **6m** (0.0188 g, 73%, 78% yield brsm) as a colorless oil. R_f: 0.46 (19:1 hexanes/EtOAc). ^{1}H NMR ($CDCl_3$, 600 MHz) δ 6.98 (d, *J* = 9.6 Hz, 2H), 6.83 (s, 2H), 6.77 (d, *J* = 8.4 Hz, 2H), 3.91 (s, 2H), 3.80 (s, 6 H), 2.23 (s, 6H); ^{13}C{1H} NMR ($CDCl_3$, 126 MHz) δ 155.7, 131.2, 129.6, 129.2, 127.4, 110.4, 55.8, 29.5, 20.7. IR (ATR): 3079, 2997, 2922, 2832, 1498, 1463, 1288, 1240, 1182, 1128, 1110, 1034, 910, 803, 766, 733, 564, 536, 460 cm^{-1}. HRMS (ESI+): *m/z* [M+H]$^+$ calculated for $[C_{17}H_{21}O_2]^+$: 257.1536; found: 257.1533

bis(5-Ethyl-2-methoxyphenyl)methane (6n)

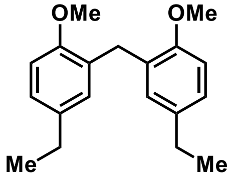

Prepared via the general procedure with 4-ethylanisole (28 µL, 0.20 mmol, 1 equiv), reacting at 50 °C on a heating block for 48 h. Purification by flash chromatography (eluting with 19:1 hexanes/EtOAc) afforded **6n** (0.0184 g, 65%) as a white solid. R_f: 0.67 (19:1 hexanes/EtOAc). M.p. 41.6–42.0 °C. ^{1}H NMR ($CDCl_3$, 600 MHz): δ 7.00 (dd, *J* = 8.4, 1.8 Hz, 2H), 6.88 (s, 2H), 6.79 (d, *J* = 7.8 Hz, 2H), 3.92 (s, 2H), 3.80 (s, 6H), 2.52 (q, *J* = 7.8 Hz, 4H), 1.15 (t, *J* = 7.2 Hz, 6H); ^{13}C{^{1}H} NMR ($CDCl_3$, 126 MHz): δ 155.8, 136.1, 130.2, 129.1, 126.1, 110.4, 55.7, 29.9, 28.1, 16.1. IR (ATR): 3012, 2992, 2960, 2928, 2868, 2834, 1607, 1499, 1455, 1371,

1302, 1255, 1183, 1131, 1030, 924, 883, 814, 761, 739, 697, 656, 592, 526 cm^{-1}. HRMS (ESI+): *m/z* $[M+H]^+$ calculated for $[C_{19}H_{25}O_2]^+$: 285.1849; found: 285.1840.

bis(5-(*tert*-Butyl)-2-methoxyphenyl)methane (6o)

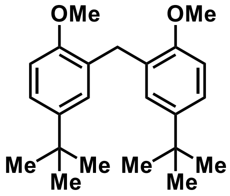

Prepared using General Procedure with 4-*tert*-butylanisole (35 µL, 0.20 mmol, 1 equiv), reacting at 75 °C on a heating block for 18 h. Purification by flash chromatography (eluting with 19:1 hexanes/EtOAc) afforded **6o** (0.0235 g, 69%) as a white solid. R_f: 0.63 (19:1 hexanes/EtOAc). M.p. 86.0–86.8 °C. ^1H NMR ($CDCl_3$, 600 MHz): δ 7.16 (m, 4H), 6.77 (d, *J* = 8.4 Hz, 2H), 3.94 (s, 2H), 3.80 (s, 6H), 1.24 (s, 18H); $^{13}C\{^1H\}$ NMR ($CDCl_3$, 126 MHz): δ 155.5, 142.7, 128.4, 128.1, 123.3, 109.6, 55.3, 34.0, 31.5, 30.6. IR (ATR): 3025, 2949, 2901, 2899, 1606, 1497, 1462, 1438, 1389, 1360, 1303, 1268, 1255, 1175, 1142, 1111, 1082, 941, 819, 764, 633, 532 cm^{-1}. HRMS (ESI+): *m/z* $[M+H]^+$ calculated for $[C_{23}H_{33}O_2]^+$: 341.2475; found: 341.2480.

bis(5-(Phenyl)-2-methoxyphenyl)methane (6p)

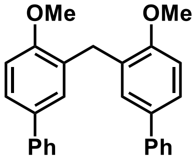

Prepared via the general procedure with 4-phenylanisole (36 mg, 0.20 mmol, 1 equiv), reacting at 50 °C on a heating block for 18 h. Purification by flash chromatography (eluting with 19:1 hexanes/EtOAc) afforded **6p** (0.0099 g, 26%) as a white solid. R_f: 0.43 (19:1 hexanes/EtOAc). M.p. 86.0–86.8 °C. ^1H NMR ($CDCl_3$, 500 MHz) δ 7.49 (d, *J* = 7.7 Hz, 4H), 7.42 (dd, *J* = 8.2, 2.4 Hz, 2H), 7.39 – 7.34 (m, 6H), 7.28 (s, 1H), 7.25 (s, 1H), 6.95 (d, *J* = 8.5 Hz, 2H), 4.07 (s, 2H), 3.88 (s, 6H); $^{13}C\{^1H\}$ NMR ($CDCl_3$, 126 MHz): δ 157.4, 141.3, 133.5, 129.4, 129.4, 128.8, 126.9, 126.6, 125.9, 110.7, 55.7, 31.1. IR (ATR): 3028, 2957, 2922, 2850, 2830, 2361, 1604, 1508, 1485, 1461, 1435, 1406, 1292, 1246, 904, 819, 758, 730, 696, 570, 561, 550, 542, 534 cm^{-1}. HRMS (ESI+): *m/z* $[M+\mathbf{NH_4}]^+$ calculated for $[C_{27}H_{28}NO_2]^+$: 398.2115; found: 398.2106.

bis(5-Fluoro-2-methoxyphenyl)methane (6q)

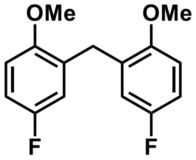

Prepared via the general procedure with 4-fluoroanisole (23 µL, 0.20 mmol, 1 equiv), reacting at 50 °C on a heating block for 48 h. Purification by flash chromatography (eluting with 17:3 hexanes/CH_2Cl_2) afforded **6q** (0.0097 g, 37%) as a yellow oil. R_f: 0.50 (17:3 hexanes/CH_2Cl_2). ^1H NMR ($CDCl_3$, 600 MHz): δ 6.87 (dt, *J* = 8.5, 3.0 Hz, 2H), 6.77 (td, *J* = 8.6, 3.8 Hz, 4H), 3.89 (s, 2H), 3.80 (s, 6H); $^{13}C\{^1H\}$ NMR ($CDCl_3$, 126 MHz): δ 157.9 (d, *J* = 198.3 Hz),

153.7, 130.4 (d, J = 5.9 Hz), 117.3 (d, J = 19.5 Hz), 113.2 (d, J = 19.0 Hz), 111.1, 56.1, 30.0; ^{19}F NMR ($CDCl_3$, 564 MHz): δ 124.4 (s). IR (ATR): 3050, 2999, 2924, 2836, 1733, 1599, 1491, 1456, 1422, 1258, 1209, 1182, 1147, 1112, 1031, 968, 888, 810, 733, 712, 587, 571, 557, 544 cm^{-1}. HRMS (ESI+): m/z $[M+H]^+$ calculated for $[C_{15}H_{15}F_2O_2]^+$: 265.1035; found: 265.1034.

bis(5-Chloro-2-methoxyphenyl)methane (6r)

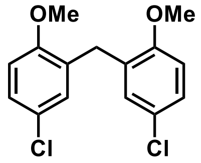

Prepared via the general procedure with 4-chloroanisole (25 µL, 0.20 mmol, 1 equiv), reacting at 75 °C on a heating block for 48 h. Purification by flash chromatography (eluting with 19:1 hexanes/EtOAc) afforded **6r** (0.0089 g, 30%) as a white solid. R_f: 0.58 (19:1 hexanes/EtOAc). M.p. 92.8–93.5 °C. ^{1}H NMR ($CDCl_3$, 600 MHz): δ 7.18 (dd, J = 9.0, 2.4 Hz, 2H), 7.02 (d, J = 1.8 Hz, 2H), 6.81 (d, J = 9.0 Hz, 2H), 3.90 (s, 2H), 3.84 (s, 6H); ^{13}C{^{1}H} NMR ($CDCl_3$, 126 MHz): δ 156.1, 130.2, 130.1, 127.0, 125.2, 111.5, 55.7, 29.6. IR (ATR): 3006, 2962, 2912, 2834, 2359, 1592, 1485, 1454, 1404, 1297, 1253, 1179, 1130, 1025, 920, 860, 807, 762, 643, 563, 530 cm^{-1}. GCMS (EI+): m/z $[M]^+$ calculated for $C_{15}H_{14}Cl_2O_2$: 296.0; found: 296.0. HRMS (ESI+): m/z $[M+H]^+$ calculated for $[C_{15}H_{15}Cl_2O_2]^+$: 297.0444; found: 297.0449.

bis(5-Bromo-2-methoxyphenyl)methane (6s)

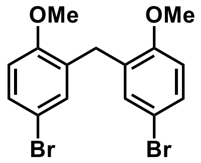

Prepared via the general procedure with 4-bromoanisole (25 µL, 0.20 mmol, 1 equiv), reacting at 75 °C on a heating block for 48 h. Purification by flash chromatography (eluting with 19:1 hexanes/EtOAc) afforded **6s** (0.0081 g, 21%) as a white solid. R_f: 0.50 (19:1 hexanes/EtOAc). M.p. 106.1–107.2 °C. ^{1}H NMR ($CDCl_3$, 600 MHz): δ 7.29 (dd, J = 8.4, 2.4 Hz, 2H), 7.13 (d, J = 2.4 Hz, 2H), 6.73 (d, J = 8.4 Hz, 2H), 3.85 (s, 2H), 3.80 (s, 6H); ^{13}C{^{1}H} NMR ($CDCl_3$, 126 MHz): δ 156.7, 133.1, 130.8, 130.2, 112.8, 112.1, 55.8, 29.7. IR (ATR): 3073, 2996, 2955, 2925, 2831, 2360, 2342, 1587, 1483, 1454, 1436, 1400, 1295, 1252, 1176, 1191, 1132, 1118, 1030, 896, 873, 850, 805, 797, 625, 563, 554, 538 cm^{-1}. HRMS (ESI+): m/z $[M+H]^+$ calculated for $[C_{15}H_{15}Br_2O_2]^+$: 384.9433; found: 384.9449. Spectral data consistent with literature.[6]

bis(5-Iodo-2-methoxyphenyl)methane (6t)

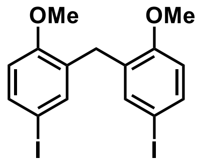

Prepared via the general procedure with 4-iodoanisole (47 mg, 0.20 mmol, 1 equiv), reacting at 75 °C on a heating block for 48 h. Purification by flash chromatography (eluting with 5:1 hexanes/CH_2Cl_2) afforded **6t** (0.0096 g, 20%) as a white solid. R_f: 0.84

(5:1 hexanes/DCM). M.p. 134.6–135.2 °C. ^{1}H NMR ($CDCl_3$, 600 MHz): δ 7.50 (dd, *J* = 8.4, 1.2 Hz, 2H), 7.34 (s, 2H), 6.66 (d, *J* = 9.0 Hz, 2H), 3.84 (s, 2H), 3.83 (s, 6H); ^{13}C{^{1}H} NMR ($CDCl_3$, 126 MHz): δ 157.6, 138.9, 136.3, 131.3, 112.8, 83.0, 55.6, 29.5. IR (ATR): 3064, 2931, 2835, 1584, 1511, 1484, 1439, 1394, 1271, 1243, 1221, 1174, 1126, 1027, 866, 802, 761, 635, 616, 560, 529 cm^{-1}. HRMS (ESI+): *m/z* [M+Na]$^{+}$ calculated for $[C_{15}H_{14}I_2O_2Na]^{+}$: 502.8975; found: 502.8971.

Dimethyl 3,3'-(methylenebis(4-methoxy-3,1-phenylene))dipropionate (6u)

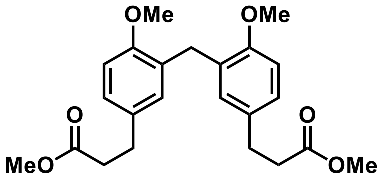

Prepared via the general procedure with methyl 3-(4-methoxyphenyl)propanoate (35 µL, 0.20 mmol, 1 equiv), reacting at 50 °C on a heating block for 48 h. Purification by flash chromatography (eluting with 9:1 hexanes/EtOAc) afforded **6u** (0.0250 g, 64%) as a clear oil. R_f: 0.10 (9:1 hexanes/EtOAc). ^{1}H NMR ($CDCl_3$, 600 MHz): δ 7.00 (d, *J* = 8.4, 2H), 6.84 (s, 2H), 6.78 (d, *J* = 8.4 Hz, 2H), 3.89 (s, 2H), 3.80 (s, 6H), 3.63 (s, 6H), 2.82 (t, *J* = 7.8Hz, 4H), 2.54 (t, *J* = 7.8 Hz, 4H); ^{13}C{^{1}H} NMR ($CDCl_3$, 126 MHz): δ 173.7, 156.2, 132.3, 130.4, 129.2, 126.8, 110.4, 55.6, 51.7, 36.2, 30.4, 29.9. IR (ATR): 3008, 2959, 2928, 2834, 2359, 2341, 1735, 1608, 1499, 1456, 1437, 1251, 1177, 1130, 1030, 882, 813, 761, 738, 697, 587, 541, 533, 526 cm^{-1}. HRMS (ESI+): *m/z* [M+H]$^{+}$ calculated for $[C_{23}H_{29}O_6]^{+}$: 401.1959; found: 401.1973.

Dimethyl 3,3'-methylenebis(4-methoxybenzoate) (6v)

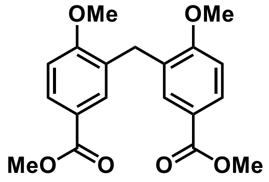

Prepared via the general procedure with 4-methoxy-methylbenzoate (33 mg, 0.20 mmol, 1 equiv), reacting at 75 °C on a heating block for 18 h. Purification by flash chromatography (eluting with 19:1 hexanes/EtOAc) afforded **6v** (0.0170 g, 52%), as a white solid. R_f: 0.31 (19:1 hexanes/EtOAc). M.p. 143.7–144.9 °C. ^{1}H NMR ($CDCl_3$, 600 MHz): δ 7.91 (d, *J* = 10.2 Hz, 2H), 7.75 (s, 2H), 6.87 (d, *J* = 8.4 Hz, 2H), 3.96 (s, 2H), 3.88 (s, 6H), 3.84 (s, 6H); ^{13}C{^{1}H} NMR ($CDCl_3$, 126 MHz): δ 167.3, 161.5, 132.0, 130.0, 128.6, 122.3, 109.9, 55.7, 51.9, 30.1. IR (ATR): 3005, 2953, 2842, 2359, 1708, 1604, 1500, 1438, 1289, 1262, 1241, 1190, 1138, 1102, 1022, 995, 900, 825, 763, 637, 560 cm^{-1}. HRMS (ESI+): *m/z* [M+H]$^{+}$ calculated for $[C_{19}H_{21}O_6]^{+}$: 345.1333; found: 345.1339. The same product (0.0141 g, 41%) is obtained with 4-methoxybenzoic acid (30 mg. 0.20 mmol, 1 equiv) via the same procedure.

bis(3,7-Dimethoxynaphthalen-2-yl)methane (6w)

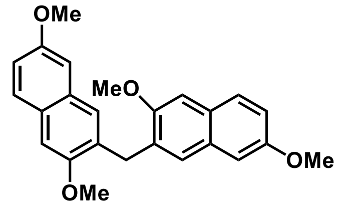

Prepared via the general procedure with 2,6-dimethoxynapthalene (38 mg, 0.20 mmol, 1 equiv), reacting at 50 °C on a heating block for 18 h. Purification by flash chromatography (eluting with 19:1 hexanes/EtOAc), followed by trituration with ethanol afforded **6w** (0.0140 g, 36%) as a tan solid. R_f: 0.10 (19:1 hexanes/EtOAc). M.p. 209.5–210.4 °C. ^{1}H NMR ($CDCl_3$, 600 MHz): δ 8.12 (d, J = 9.6 Hz, 2H), 7.62 (d, J = 9.0 Hz, 2H), 7.27 (m, 2H), 7.04 (s, 2H), 7.01 (dd, J = 9.6, 2.4 Hz, 2H), 4.87 (s, 2H), 3.88 (s, 6H), 3.87 (s, 6H); ^{13}C{^{1}H} NMR ($CDCl_3$, 126 MHz): δ 156.7, 153.3, 130.7, 129.3, 126.7, 126.2, 124.5, 118.5, 114.9, 106.3, 57.2, 55.3, 21.9. IR (ATR): 3062, 2931, 2837, 2360, 2342, 1626, 1595, 1506, 1453, 1374, 1341, 1253, 1091, 1022, 943, 854, 730, 663 cm^{-1}. HRMS (ESI+): m/z [M+Na]$^+$ calculated for [$C_{25}H_{24}O_4Na$]$^+$: 411.1567; found: 411.1561.

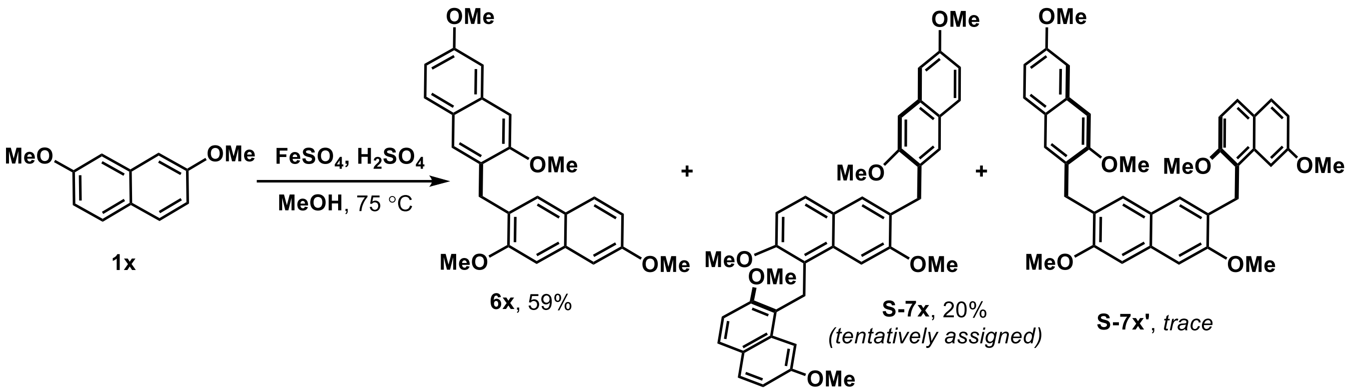

Prepared via the general procedure with 2,7-dimethoxynapthalene (376 mg, 2.0 mmol, 1 equiv), reacting at 75 °C on a heating block for 72 h. Purification by flash chromatography (eluting with 5:1 hexanes/EtOAc) afforded **6x** (226 mg, 59%) as a colorless liquid and **S-7x** (77 mg, 20%) as a colorless liquid.

bis(3,6-Dimethoxynaphthalen-2-yl)methane (6x)

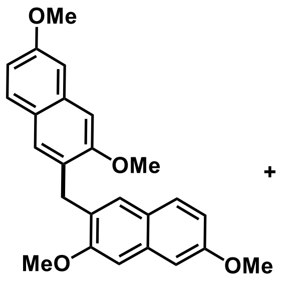

R_f: 0.28 (5:1 hexanes/EtOAc). ^{1}H NMR ($CDCl_3$, 600 MHz) δ 7.64 (d, J = 9.0 Hz, 2H), 7.55 (d, J = 9.0 Hz, 2H), 7.41 (d, J = 2.5 Hz, 2H), 7.17 (d, J = 9.0 Hz, 2H), 6.85 (dd, J = 9.0, 2.5 Hz, 2H), 4.91 (s, 2H), 4.02 (s, 6H), 3.64 (s, 6H); ^{13}C{^{1}H} NMR ($CDCl_3$, 126 MHz) δ 157.8, 154.9, 135.1, 129.7, 127.9, 125.2, 122.7, 116.4, 110.8, 103.1, 57.0, 55.0, 21.5. IR (ATR): 3057, 2934, 2836, 2361, 1627, 1587, 1513, 1487, 1462, 1386, 1331, 1244, 1227, 1184 cm^{-1}. HRMS (ESI+): m/z [M+Na]$^+$ calculated for [$C_{25}H_{24}O_4Na$]$^+$: 411.1567; found: 411.1555.

Unsymmetrical trimer S-7x

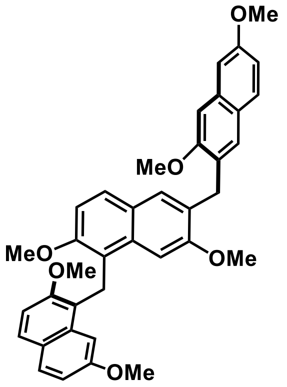

R_f: 0.24 (5:1 hexanes/EtOAc). ^{1}H NMR (400 MHz, $CDCl_3$) δ 7.73 (d, *J* = 8.9 Hz, 1H), 7.67 (t, *J* = 9.2 Hz, 2H), 7.56 (d, *J* = 8.9 Hz, 1H), 7.41 (s, 2H), 7.27 (d, *J* = 9.1 Hz, 1H), 7.19 (dd, *J* = 8.9, 3.1 Hz, 2H), 7.00 (d, *J* = 8.9 Hz, 1H), 6.98 (d, *J* = 2.5 Hz, 1H), 6.94 (dd, *J* = 8.8, 2.4 Hz, 1H), 6.84 (dd, *J* = 8.9, 2.5 Hz, 1H), 6.81 (s, 1H), 4.90 (s, 2H), 4.33 (s, 2H), 4.04 (s, 3H), 3.96 (s, 3H), 3.85 (s, 3H), 3.77 (s, 3H), 3.62 (s, 3H), 3.60 (s, 3H); ^{13}C{^{1}H} NMR ($CDCl_3$, 101 MHz) δ 158.2, 157.8, 156.6, 155.9, 154.9, 154.1, 135.2, 133.6, 129.9, 129.6, 127.9, 127.8_4, 127.7_9, 127.5, 125.2, 124.8, 122.9, 122.2, 120.4, 116.5, 116.1, 111.1, 110.8, 110.5, 103.1, 102.7, 102.1, 57.1, 57.0, 56.7, 55.2_2, 55.2_1, 55.0, 24.5, 21.5. IR (ATR): 2995, 2934, 2833, 1625, 1600, 1512, 1459, 1419, 1385, 1334, 1304, 1243, 1219, 1180, 1136, 1093, 1044, 1029, 978, 949, 920, 901, 824, 793, 766, 730, 611, 541, 512, 488, 460, 442 cm^{-1}. HRMS (ESI+): *m/z* [M+Na]$^+$ calculated for $[C_{38}H_{36}O_6Na]^+$: 611.2404; found 611.2404. The structure of this compound is tentatively assigned based on the presence of 2 singlets in the aromatic region of the ^{1}H NMR spectrum. Unsymmetrical **S-7x'** is expected to have only 1 singlet aromatic signal.

bis(4-(*tert*-Butyl)-2-ethoxyphenyl)methane (6y)

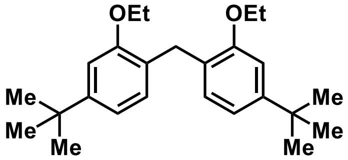

Prepared via the general procedure with 1-(*tert*-butyl)-3-ethoxybenzene (35 mg, 0.20 mmol, 1 equiv), reacting at 75 °C on a heating block for 18 h. Purification by flash chromatography (eluting with 19:1 hexanes/EtOAc) afforded **6y** (0.0232 g, 63%) as a colorless oil. R_f: 0.85 (19:1 hexanes/EtOAc). ^{1}H NMR ($CDCl_3$, 600 MHz): δ 7.08 (d, *J* = 8.4 Hz, 2H), 6.87–6.86 (m, 4H), 4.06 (q, *J* = 7.2 Hz, 4H), 3.91 (s, 2H), 1.42 (t, *J* = 7.2 Hz, 6H), 1.30 (s, 18H); ^{13}C{^{1}H} NMR ($CDCl_3$, 126 MHz): δ 156.7, 150.1, 130.3, 127.1, 117.1, 109.1, 63.8, 34.8, 31.6, 29.2, 15.2. IR (ATR): 3086, 2962, 2870, 1609, 1574, 1500, 1408, 1391, 1266, 1230, 1136, 1105, 1047, 962, 909, 855, 813, 732, 674, 648 cm^{-1}. HRMS (ESI+): *m/z* [M+H]$^+$ calculated for $[C_{25}H_{37}O_2]^+$: 369.2788; found: 369.2778.

bis(4-(3-Bromopropoxy)phenyl)methane (6z)

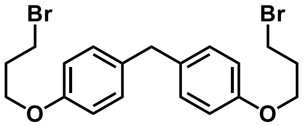

Prepared via the general procedure with 3-bromophenoxypropane (32 µL, 0.20 mmol, 1 equiv), reacting at 50 °C on a heating block for 18 h. Purification by flash chromatography (eluting with 9:1 hexanes/Et_2O) afforded **6z** (0.0096 g,

47%) as a yellow oil. R_f: 0.36 (9:1 hexanes/Et_2O). ^1H NMR ($CDCl_3$, 600 MHz): δ 7.08 (d, J = 8.4 Hz, 4H), 6.82 (d, J = 8.4 Hz, 4H), 4.07 (t, J = 5.4 Hz, 4H), 3.86 (s, 2H), 3.60 (t, J = 6.6 Hz, 4H), 2.30 (qt, J = 6.0 Hz, 4H); $^{13}C\{^1H\}$ NMR ($CDCl_3$, 126 MHz): δ 157.2, 134.1, 129.9, 114.6, 65.4, 40.3, 32.6, 31.1. IR (ATR): 3068, 2923, 1609, 1583, 1506, 1468, 1435, 1387, 1299, 1235, 1172, 1109, 1029, 929, 851, 818, 778, 737, 668, 655, 598, 552, 529 cm^{-1}. HRMS (ESI+): m/z [M+**NH_4**]$^+$ calculated for $[C_{19}H_{26}Br_2NO_2]^+$: 458.0325; found: 458.0319.

bis(4-Phenoxyphenyl)methane (6za)

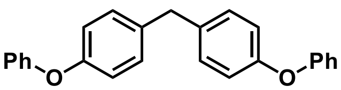

Prepared via the general procedure with diphenyl ether (34 mg, 0.20 mmol, 1 equiv), reacting at 75 °C on a heating block for 18 h. Purification by flash chromatography (eluting with 19:1 hexanes/Et_2O) afforded **6za** (0.0163 g, 46%) as a colorless oil. R_f: 0.57 (19:1 hexanes/Et_2O). ^1H NMR (500 MHz, $CDCl_3$) δ 7.35 (t, J = 7.8 Hz, 4H), 7.18 (d, J = 8.2 Hz, 4H), 7.11 (t, J = 7.4 Hz, 2H), 7.02 (d, J = 8.1 Hz, 4H), 6.97 (d, J = 8.3 Hz, 4H), 3.94 (s, 2H); $^{13}C\{^1H\}$ NMR ($CDCl_3$, 126 MHz) δ 157.6, 155.6, 136.2, 130.2, 129.8, 123.2, 119.2, 118.8, 40.6. IR (ATR): 3038, 2918, 1734, 1586, 1504, 1455, 1432, 1332, 1284, 1233, 1101, 1015, 911 cm^{-1}. HRMS (ESI+): m/z [M+H]$^+$ calculated for $[C_{25}H_{21}O_2]^+$: 353.1536; found: 353.1537.

bis(2,4,6-Trimethoxyphenyl)methane (6zb)

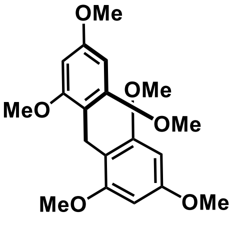

Prepared via the general procedure with 1,3,5-trimethoxybenzene (33.6 mg, 0.20 mmol, 1 equiv), reacting at 50 °C on a heating block for 48 h. Purification by flash chromatography (eluting with 4:1 hexanes/CH_2Cl_2) afforded **6zb** (0.0214 g, 61%) as a white solid. R_f: 0.67 (4:1 hexanes/CH_2Cl_2). ^1H NMR ($CDCl_3$, 600 MHz) δ 6.09 (s, 4H), 3.83 (s, 2H), 3.77(s, 6H), 3.70 (s, 12H); $^{13}C\{^1H\}$ NMR ($CDCl_3$,126 MHz) δ 159.4, 158.8, 112.1, 91.3, 56.3, 56.3, 16.8. IR (ATR): 3002, 2956, 2925, 2849, 1593, 1496, 1454, 1411, 1327, 1224, 1197, 1146, 1106, 1057, 1033, 946, 877, 812, 793, 720, 631, 530, 499 cm^{-1}. HRMS (ESI+): m/z [M+H]$^+$ calculated for $[C_{19}H_{25}O_6]^+$: 349.1646; found 349.1642. λ_{max} (UV/Vis) = 210, 230, 270 nm.

bis(4-Methoxy-3-methylphenyl)methane-*d2* (6a-d2)

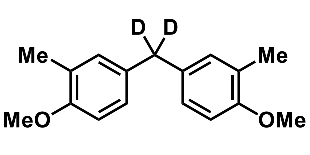

Prepared via the general procedure with 2-methylanisole (25 µL, 0.20 mmol, 1 equiv) using CD_3OD as solvent, reacting at 50 °C on a heating block for 18 h. Purification by flash chromatography (eluting with 19:1 hexanes/EtOAc)

afforded **6a-d2** (0.0201 g, 78%) as a colorless oil. R_f: 0.38 (19:1 hexanes/EtOAc). ^{1}H NMR ($CDCl_3$, 600 MHz): δ 6.96-6.97 (m, 4H), 6.74 (d, J = 7.8 Hz, 2H), 3.80 (s, 6H), 2.18 (s, 6H); ^{2}H NMR ($CDCl_3$, 93 MHz): δ 3.78; ^{13}C{^{1}H} NMR ($CDCl_3$, 126 MHz): δ 156.2, 133.5, 131.2, 126.9, 126.6, 110.0, 55.5, 16.4. IR (ATR): 3004, 2947, 2832, 1609, 1501, 1482, 1463, 1440, 1377, 1300, 1235, 1183, 1132, 1032, 884, 811, 779, 748, 622, 576, 540 cm^{-1}. HRMS (ESI+): m/z $[M+H]^+$ calculated for $[C_{17}H_{19}D_2O_2]^+$: 259.1662; found: 259.1668.

Reaction with anisole (1zc)

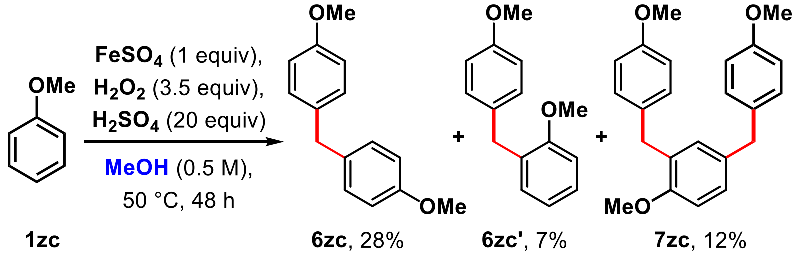

The above reaction was carried out via the general procedure with anisole (0.22 mL, 2.0 mmol, 1 equiv), reacting at 50 °C on a heating block for 48 h. Purification by flash chromatography (eluting with 19:1 hexanes/Et_2O) afforded **6zc** (0.0637 g, 28%) as a colorless oil, **6zc'** (0.0168 g, 7%) as a colorless film, and **7zc** (0.0597 g, 12%) as a colorless oil.

bis(4-Methoxyphenyl)methane (6zc)

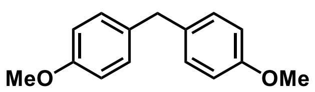

^{1}H NMR (400 MHz, $CDCl_3$) δ 7.09 (d, J = 8.8 Hz, 4H), 6.83 (d, J = 8.7 Hz, 4H), 3.87 (s, 2H), 3.78 (s, 6H); ^{13}C{^{1}H} NMR (176 MHz, $CDCl_3$) δ 158.1, 133.9, 129.9, 114.0, 55.4, 40.3. IR (ATR): 3001, 2957, 2905, 2836, 2253, 1610, 1509, 1463, 1300, 1244, 1174, 1107, 809 cm^{-1}. HRMS (ESI+): m/z $[M+NH_4]^+$ calculated for $[C_{15}H_{20}NO_2]^+$: 246.1489; found: 246.1488.

1-Methoxy-2-(4-methoxybenzyl)benzene (6zc')

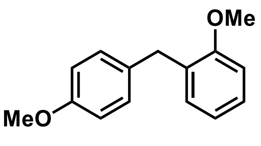

^{1}H NMR (600 MHz, $CDCl_3$) δ 7.21 (td, J = 8, 2 Hz, 1H), 7.15 (d, J = 8.8 Hz, 2H), 7.07 (dd, J = 7.6, 1.6 Hz, 1H), 6.90 (m, 2H), 6.83 (dt, J = 8.4, 2 Hz, 2H), 3.94 (s, 2H), 3.84 (s, 3H), 3.80 (s, 3H); ^{13}C{^{1}H} NMR (176 MHz, $CDCl_3$) δ 157.8, 157.3, 133.1, 130.2, 130.1, 127.3, 120.5, 113.7, 110.4, 55.4, 55.3, 34.9. IR (ATR): 2998, 2951, 2909, 2834, 1600, 1585, 1510, 1492, 1462, 1299, 1243, 1176, 807, 753 cm^{-1}. HRMS (ESI+): m/z $[M+H]^+$ calculated for $[C_{15}H_{17}O_2]^+$: 229.1223; found: 229.1219.

4,4'-((4-methoxy-1,3-phenylene)bis(methylene))bis(methoxybenzene) (7zc)

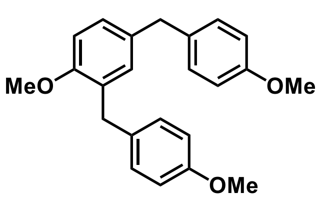

^{1}H NMR (400 MHz, $CDCl_3$) δ 7.12 (d, J = 8.8 Hz, 2H), 7.08 (d, J = 8.8 Hz, 2H), 6.97 (dd, J = 8, 2.4 Hz, 1H), 6.92 (d, J = 2.4 Hz 1H), 6.84–6.81 (m, 4H), 6.78 (d, J = 8 Hz, 1H), 3.88 (s, 2H), 3.83 (s, 2H), 3.80 (m, 9H); $^{13}C\{^{1}H\}$ NMR (176 MHz, $CDCl_3$) δ 157.8, 157.7, 155.7, 133.8, 133.4, 133.2, 131.2, 130.8, 129.8, 129.7, 127.4, 113.8, 113.6, 110.5, 55.5, 55.3, 55.3, 40.2, 35.0. IR (ATR): 2999, 2951, 2929, 2909, 2833, 1609, 1585, 1509, 1462, 1438, 1299, 1244, 810 cm^{-1}. HRMS (ESI+): m/z $[M+NH_4]^+$ calculated for $[C_{23}H_{28}NO_3]^+$: 366.2064; found: 366.2049.

4,4'-(Ethane-1,1-diyl)bis(1-methoxy-2-methylbenzene) (8a)

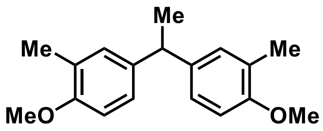

Prepared via the general procedure with 2-methylanisole (25 µL, 0.20 mmol, 1 equiv) using EtOH as a solvent, reacting at 75 °C on a heating block for 72 h. Purification by flash chromatography (eluting with 19:1 hexanes/EtOAc) afforded **8a** (0.0089 g, 33%) as a clear oil. R_f: 0.38 (19:1 hexanes/EtOAc). ^{1}H NMR ($CDCl_3$, 600 MHz): δ 7.01-6.95 (m, 4H), 6.74 (d, J = 8.4 Hz, 2H), 3.99 (q, J = 7.2 Hz, 1H), 3.79 (s, 6H), 2.18 (s, 6H), 1.57 (d, J = 7.2 Hz, 3H); $^{13}C\{^{1}H\}$ NMR ($CDCl_3$, 126 MHz): δ 156.1, 138.8, 130.1, 126.4, 125.5, 110.0, 55.5, 43.3, 22.5, 16.4. IR (ATR): 3006, 2959, 2926, 2833, 1609, 1503, 1464, 1414, 1373, 1293, 1245, 1219, 1134, 1035, 994, 926, 886, 810, 754, 606, 494, 423, 409 cm^{-1}. HRMS (ESI+): m/z $[M+H]^+$ calculated for $[C_{18}H_{22}O_2]^+$: 271.1693; found: 271.1689.

3. Deuteration Study

a. Procedure

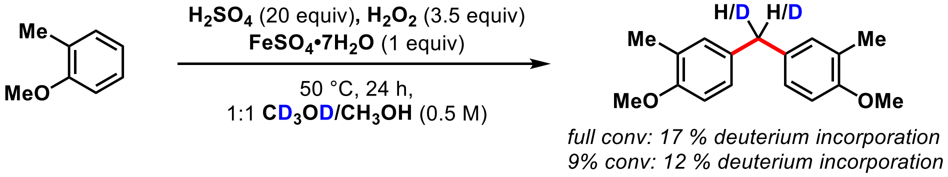

This experiment was carried out using the general procedure detailed for 2-methylanisole (25 µL, 0.20 mmol, 1 equiv) in a 1:1 mixture of CH_3OH/CD_3OD as solvent (0.4 mL total), reacting at 50 °C on a heating block for 24 h. NMR analysis of the crude reaction mixture and HRMS analysis revealed 17% deuterium incorporation at full conversion. At 9% conversion the deuterium incorporation was 12% (KIE ~7).

b. *NMR of Isolated Product*

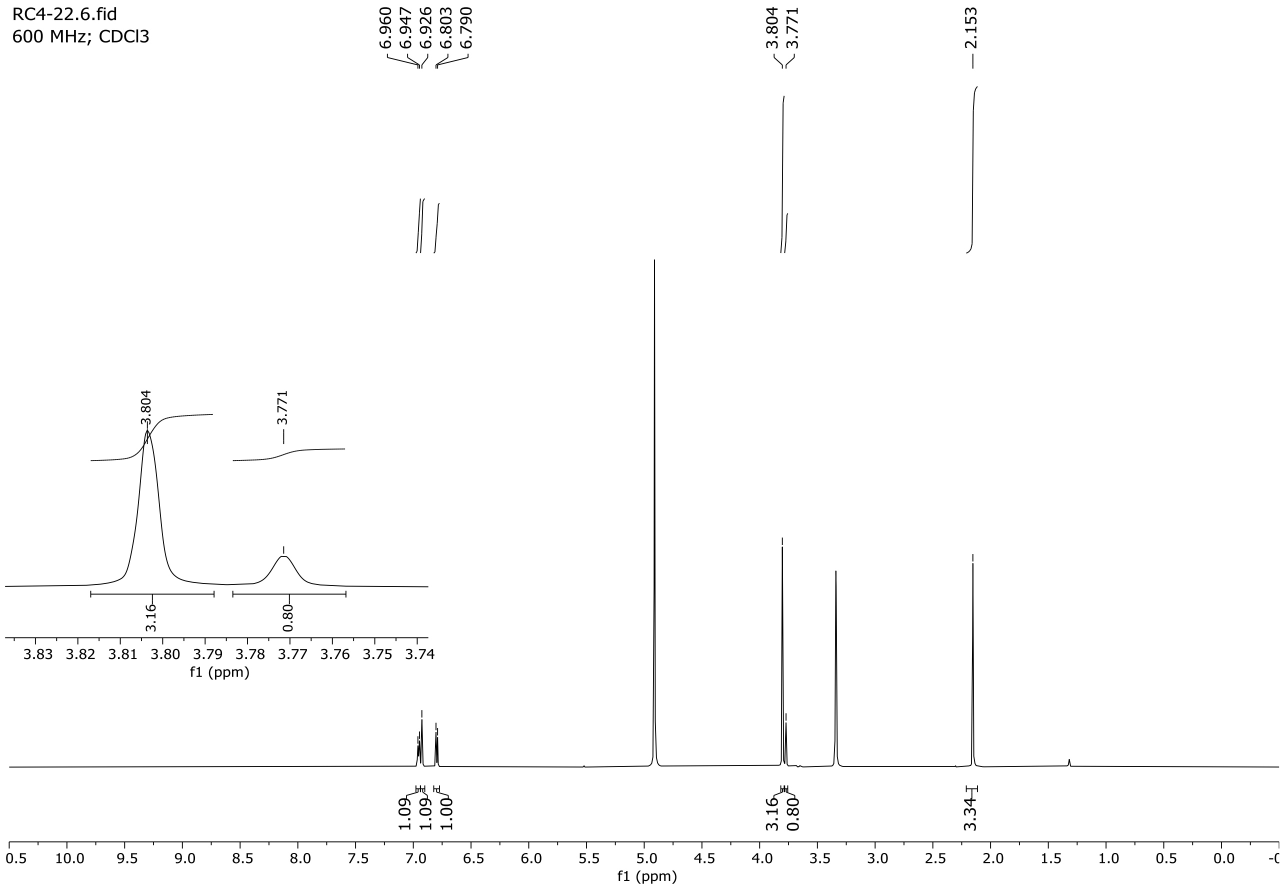

4. TEMPO Trapping Experiment

a. Procedure

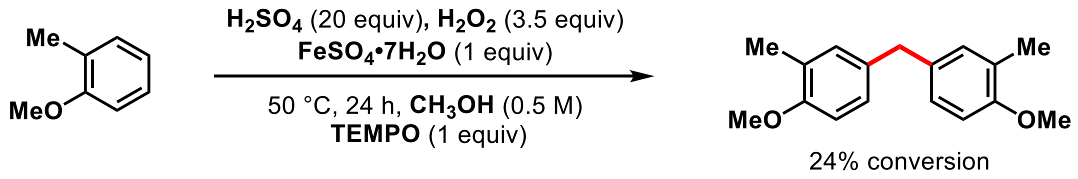

In a 1-dram vial, iron sulfate heptahydrate (55.6 mg, 0.2 mmol, 1 equiv) was suspended in methanol (0.4 mL, 0.5 M). The solution was chilled to 0 ºC in an ice-water bath before addition of H_2SO_4 (217 µL, 4.0 mmol, 20 equiv) and 30% H_2O_2 (75 µL, 0.7 mmol, 3.5 equiv). The resultant solution was warmed to rt and stirred for 15 min before arene (0.2 mmol, 1 equiv) and TEMPO (0.2 mmol, 1 equiv) addition. The reaction mixture was heated at 50 °C on a heating block for 18 h, at which time the solution was diluted with 15% $NaOH_{(aq)}$ solution (0.5 mL) and water (3 mL), then extracted with CH_2Cl_2 (3 x 5 mL). The

combined organic extract was washed with brine (5 mL) and dried over Na_2SO_4. The crude mixture was analyzed via ^1H NMR to determine the extent of conversion to product.

5. Cyclic Voltammetry Studies

Cyclic voltammogram of iron sulfate was recorded on an IKA Electrasyn 2.0 at room temperature in 1 M HCl. A Pt foil electrode was used as the working electrode and the counter electrode was a Pt plated copper electrode. A silver electrode in 3 M KCl was used as the reference electrode and all potentials are expressed versus this reference system.

All other cyclic voltammograms were recorded on an IKA Electrasyn 2.0 at room temperature in MeCN. *n*-Bu_4BF_4 was used as the supporting electrolyte. A Pt foil electrode was used as the working electrode and the counter electrode was a Pt plated copper electrode. A silver electrode in 3 M KCl was used as the reference electrode and all potentials are expressed versus this reference system.

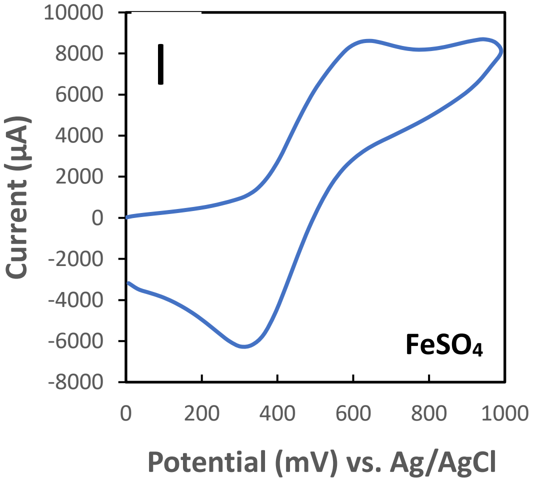

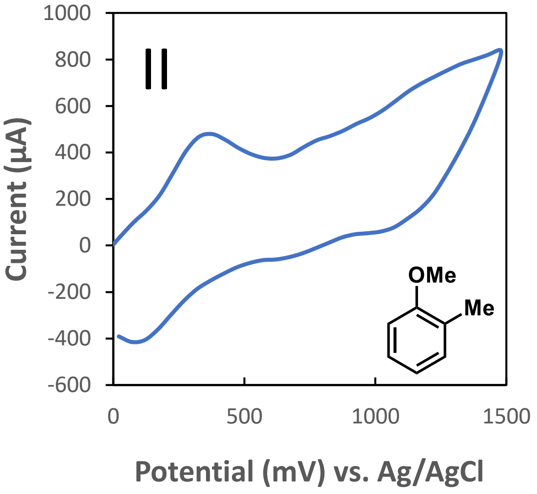

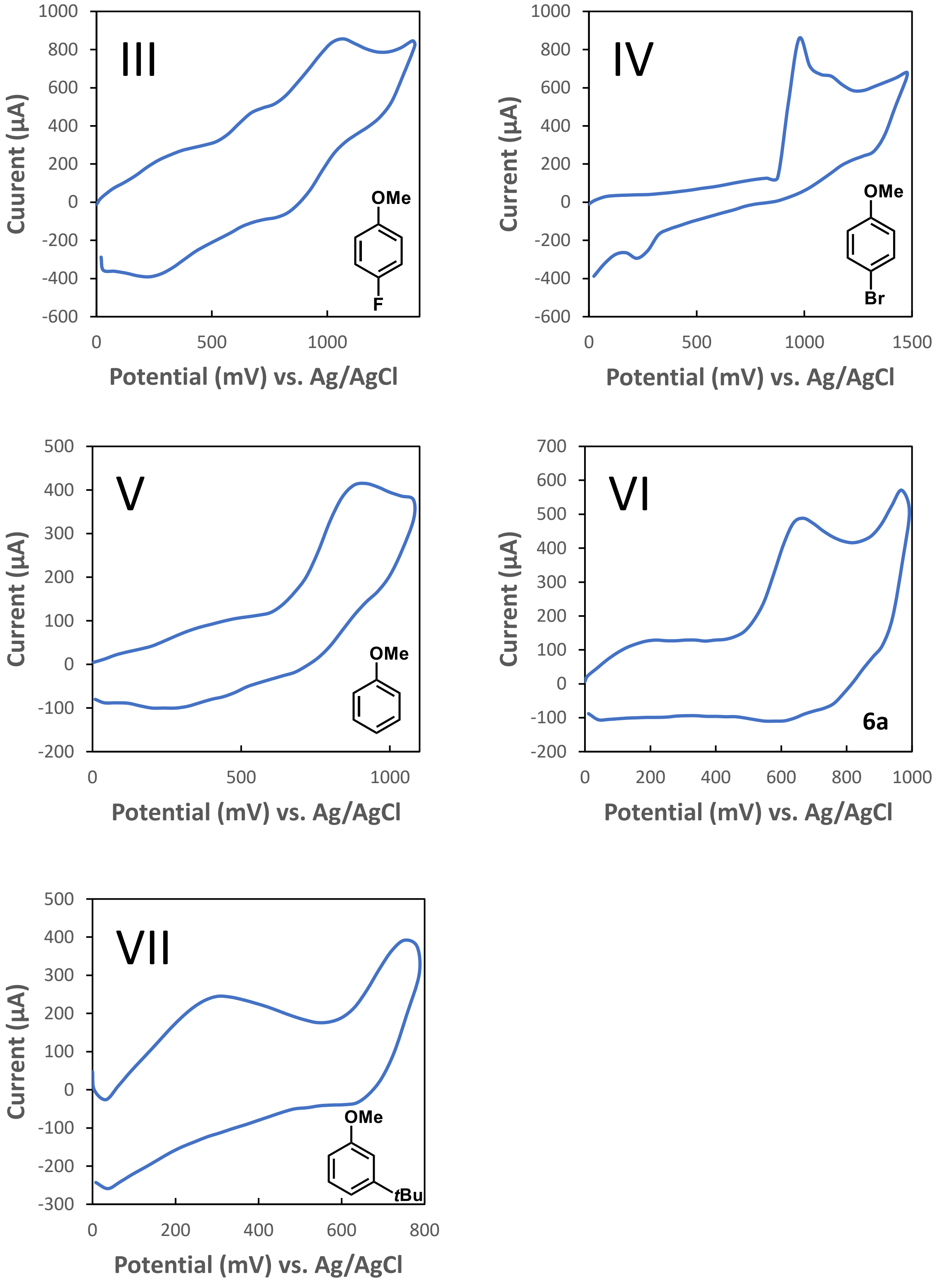

Cyclic voltammogram recorded on Pt foil working electrode and Pt plated copper counter electrode with Ag/AgCl reference electrode. The scan rate was 200 mV s^{-1}. **I**: $FeSO_4$ (30 μM) in 1 M H_2SO_4; **II**: 2-methyl anisole (30 μM) in 0.1 M *n*-Bu_4BF_4 MeCN solution; **III**: 4-fluoroanisole (30 μM) in 0.1 M *n*-Bu_4BF_4

MeCN solution; **IV**: 4-bromoanisole (30 μM) in 0.1 M *n*-Bu_4BF_4 MeCN solution; **V**: anisole (30 μM) in 0.1 M *n*-Bu_4BF_4 MeCN solution; **VI**: diarylmethane **6a** (30 μM) in 0.1 M *n*-Bu_4BF_4 MeCN solution; **VII**: 3-*tert*-butyl anisole (30 μM) in 0.1 M *n*-Bu_4BF_4 MeCN solution.

The CV of $FeSO_4$ was recorded in aqueous solution (1 M HCl) due to a lack of solubility in MeCN.

6. UV-Vis Spectroscopy

Samples used for UV-vis spectroscopy were prepared via the general procedure with 1,3,5-trimethoxybenzene (34 mg, 0.2 mmol, 1 equiv), using a cap with a septum, reacting at 50 °C for 3.5 h. The reaction is removed from the heat and 0.5 μL of the solution is dissolved in methanol (3 mL) in a quartz cuvette.

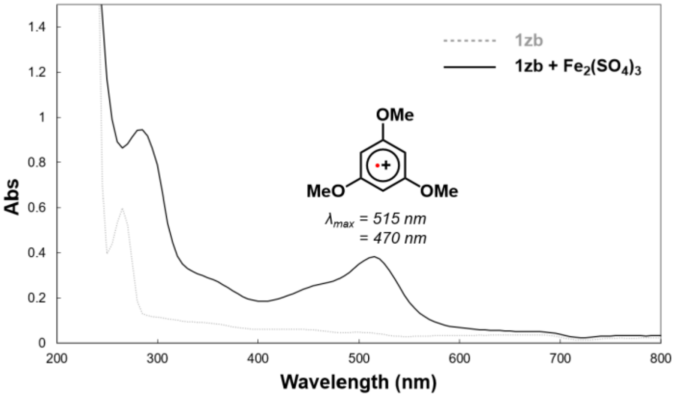

The analogous experiments with 2-methylanisole (**1a**) and 1,3-dimethoxybenzene (**1l**) did not yield signals that are indicative of radical cation species. The UV-vis absorbance spectra of the reaction mixture bear close resemblance to their respective arene reactants.

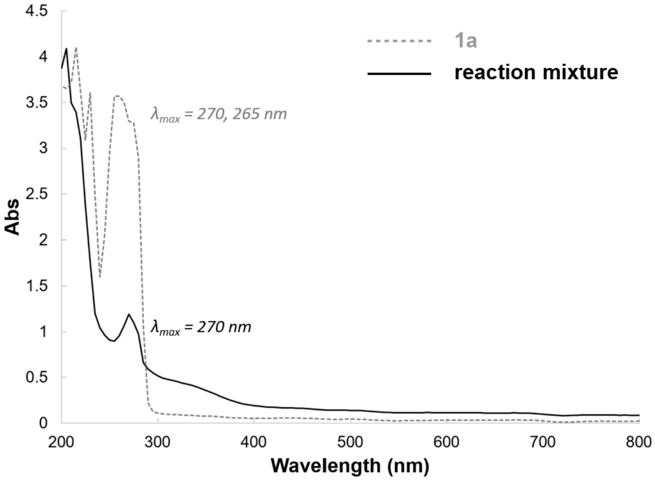

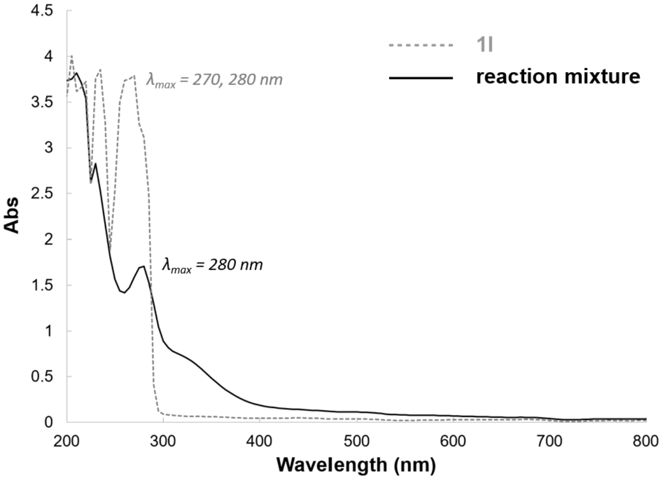

Exemplified with trimethoxybenzene (**1zb**) and its corresponding diarylmethane (**6zb**), their UV/Vis spectra bear similar features.

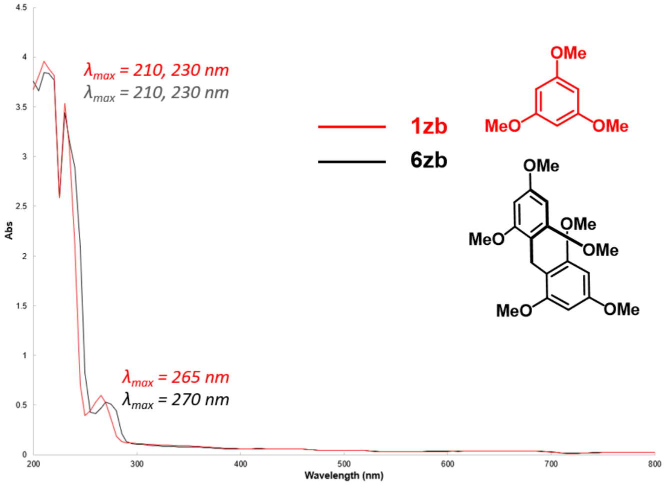

7. References

1. Pan, A.; Chojnacka, M.; Crowley, R., III; Göttemann, L.; Haines, B. E.; Kou, K. G. M. *Chem. Sci.* **2022**, *13*, 3539.

2. Tashiro, M.; Fukata, G. *J. Org. Chem.*, **1977**, *42*, 1208–1213.

3. Tummatorn, J.; Thongsornkleeb, C.; Ruchirawat, S. *Tetrahedron* **2012**, *68*, 4732–4739.

4. Dethe, D. H.; Shukla, M.; Dherange, B. D. *Org. Lett.*, **2020**, *22*, *15*, 5778–5782.

5. Doan, T.; Chardon, A.; Berionni, G. *Chem. Eur. J.*, **2020**, *27*, 1736–1743.

6. Tashiro, M.; Tsuge, A.; Yamoto, T. *J. Org. Chem.*, **1990**, *55*, 2404–2409.

8. NMR Spectra

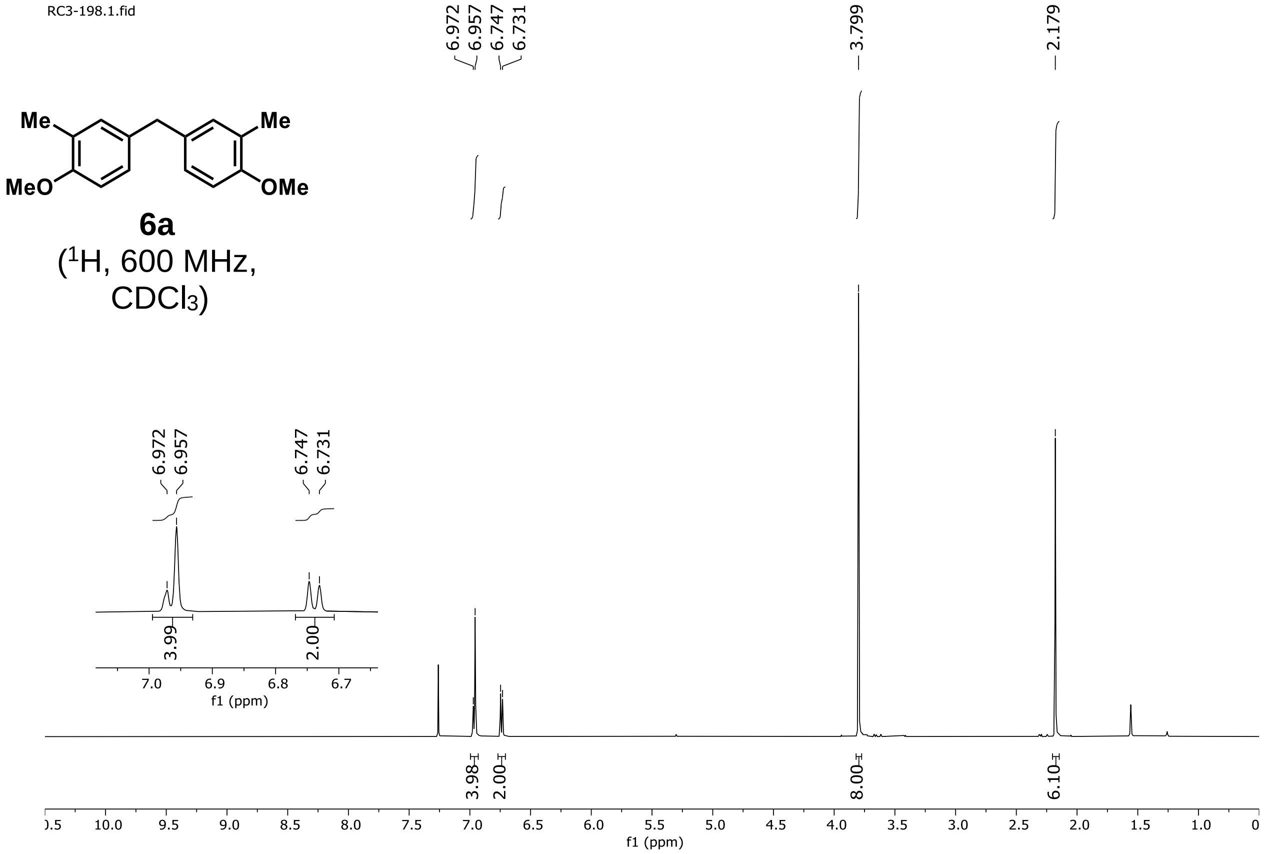

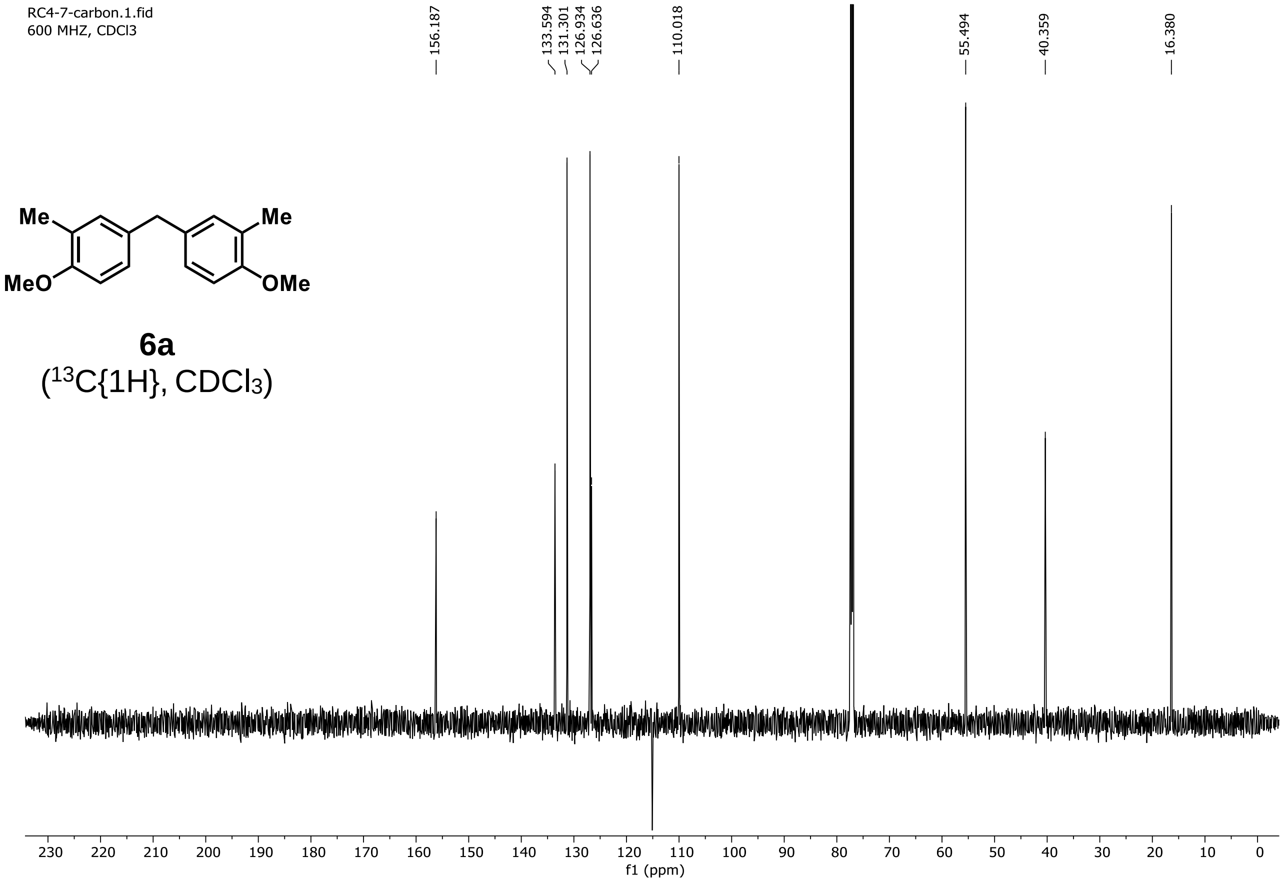

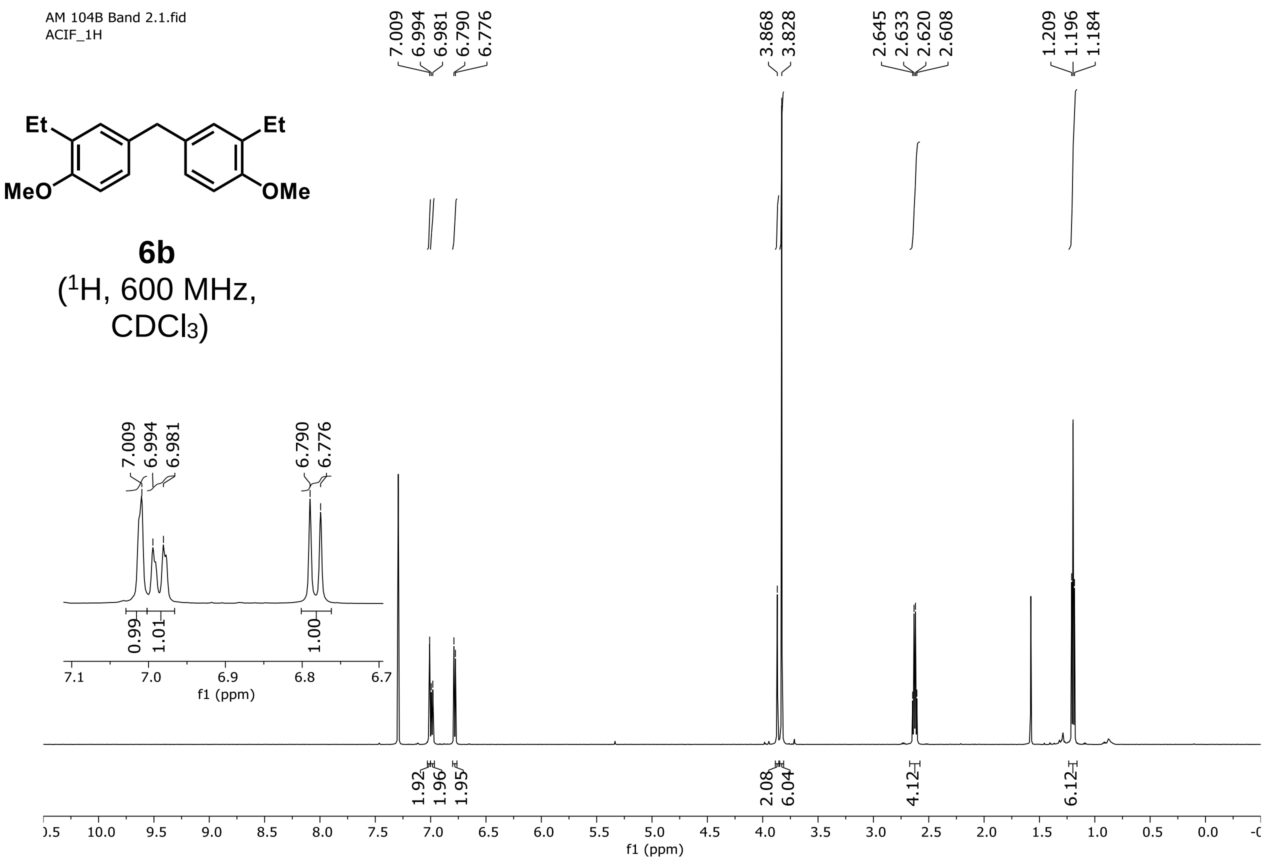

6b
(^{1}H, 600 MHz, $CDCl_3$)

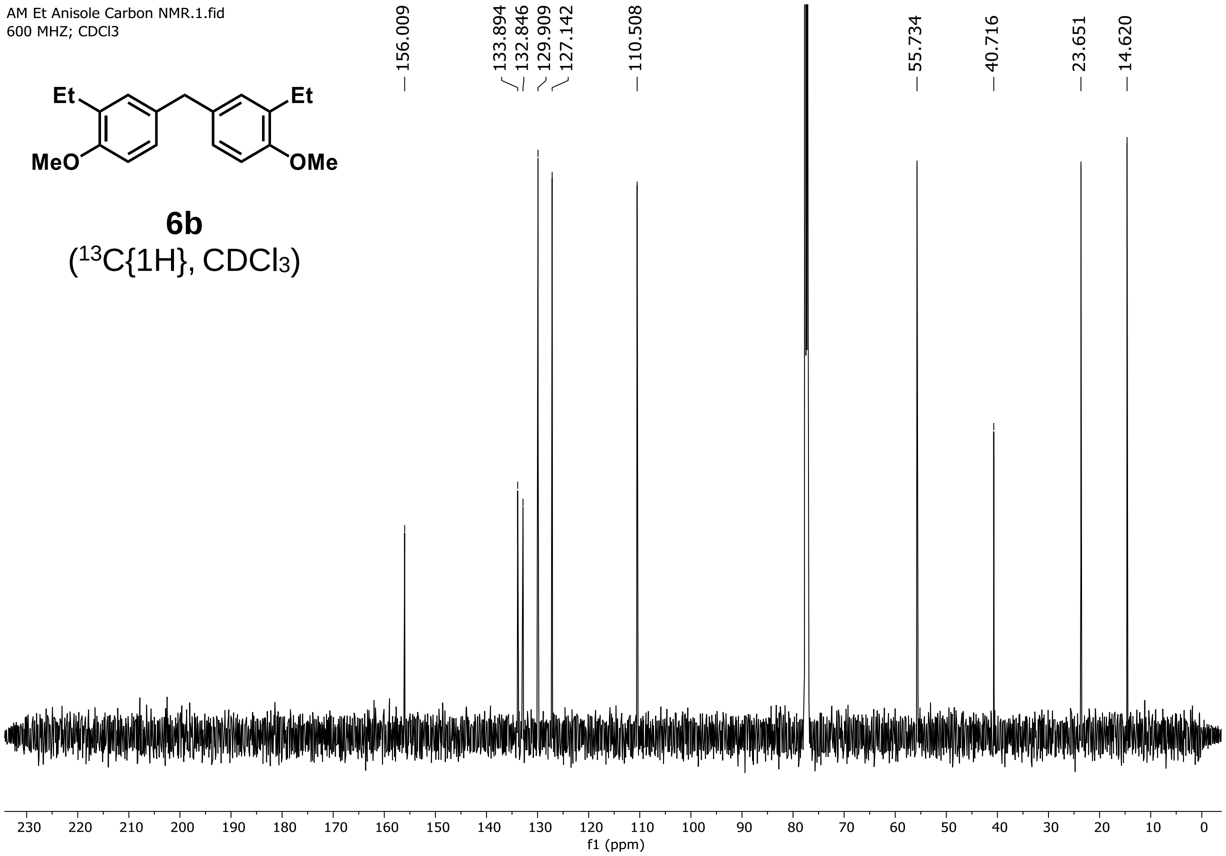

6b
(^{13}C{1H}, $CDCl_3$)

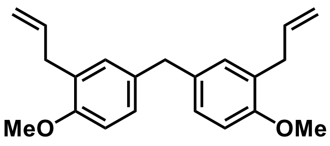

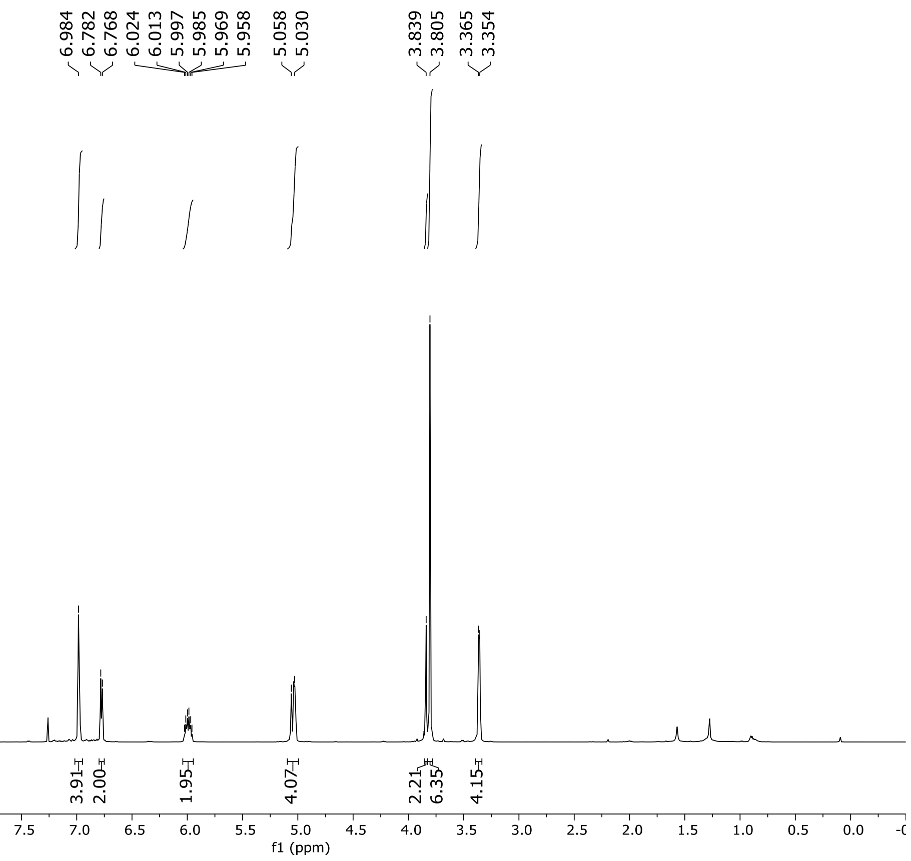

6c
(^{1}H, 600 MHz, $CDCl_3$)

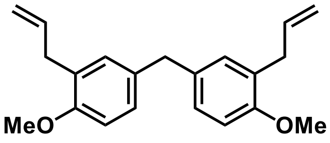

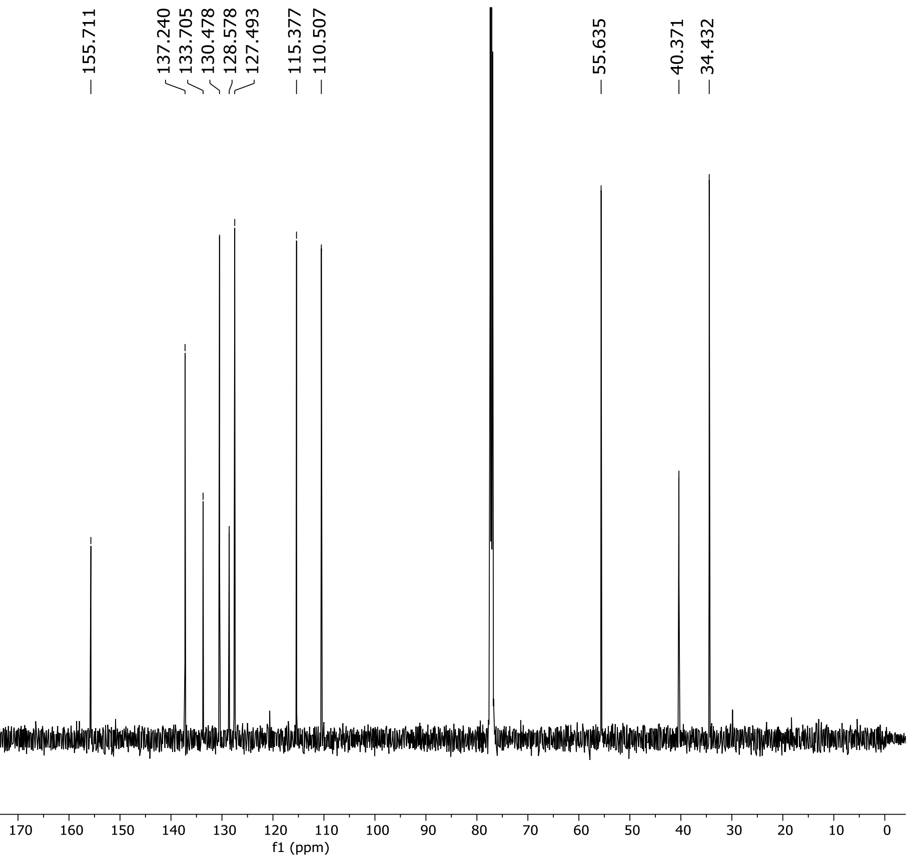

6c
(^{13}C{1H}, 600 MHz, $CDCl_3$)

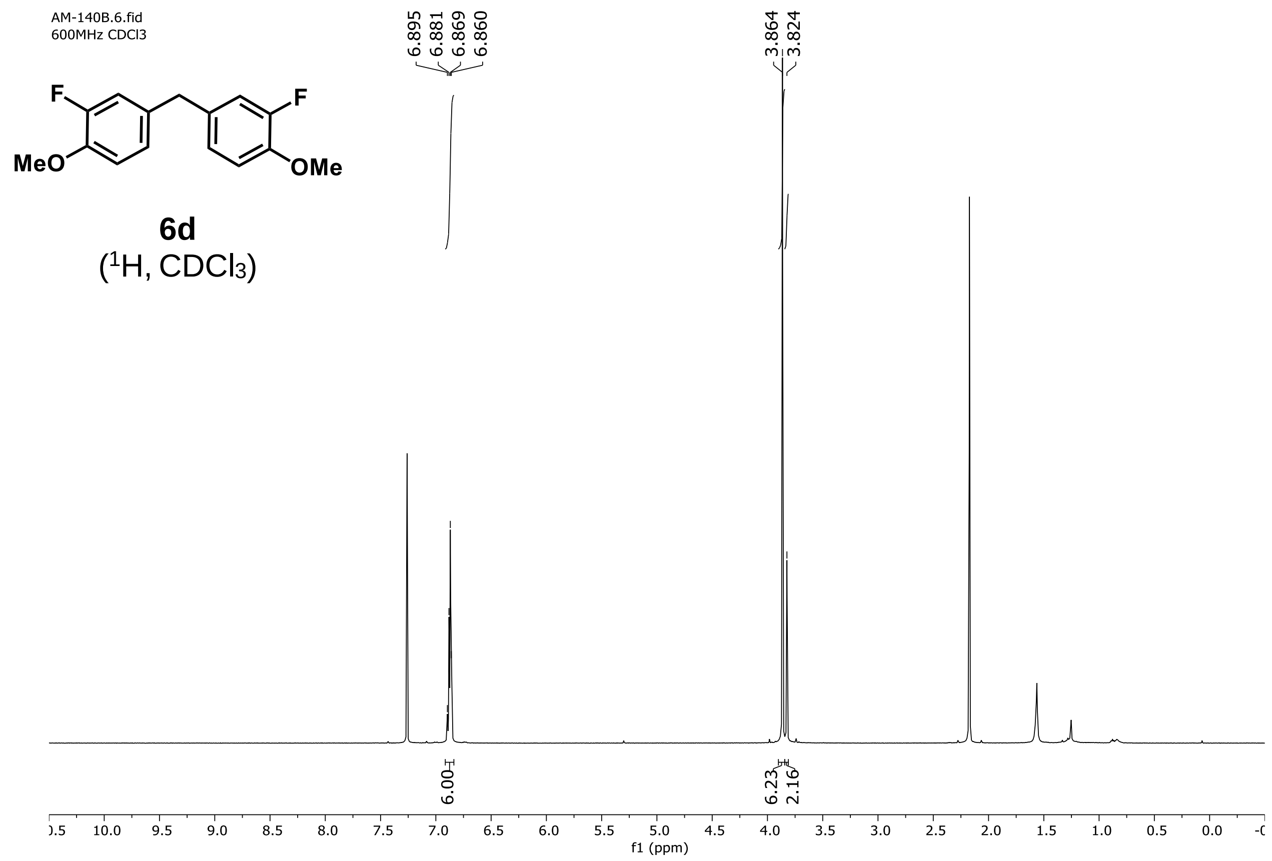
AM-140B.6.fid
600MHz CDCl3
6.895
6.881
6.869
6.860
3.864
3.824
F
F
MeO
OMe
6d
(1H, CDCl3)
6.00
6.23
2.16
10.0
9.5
9.0
8.5
8.0
7.5
7.0
6.5
6.0
5.5
5.0
4.5
4.0
3.5
3.0
2.5
2.0
1.5
1.0
0.5
0.0
f1 (ppm)

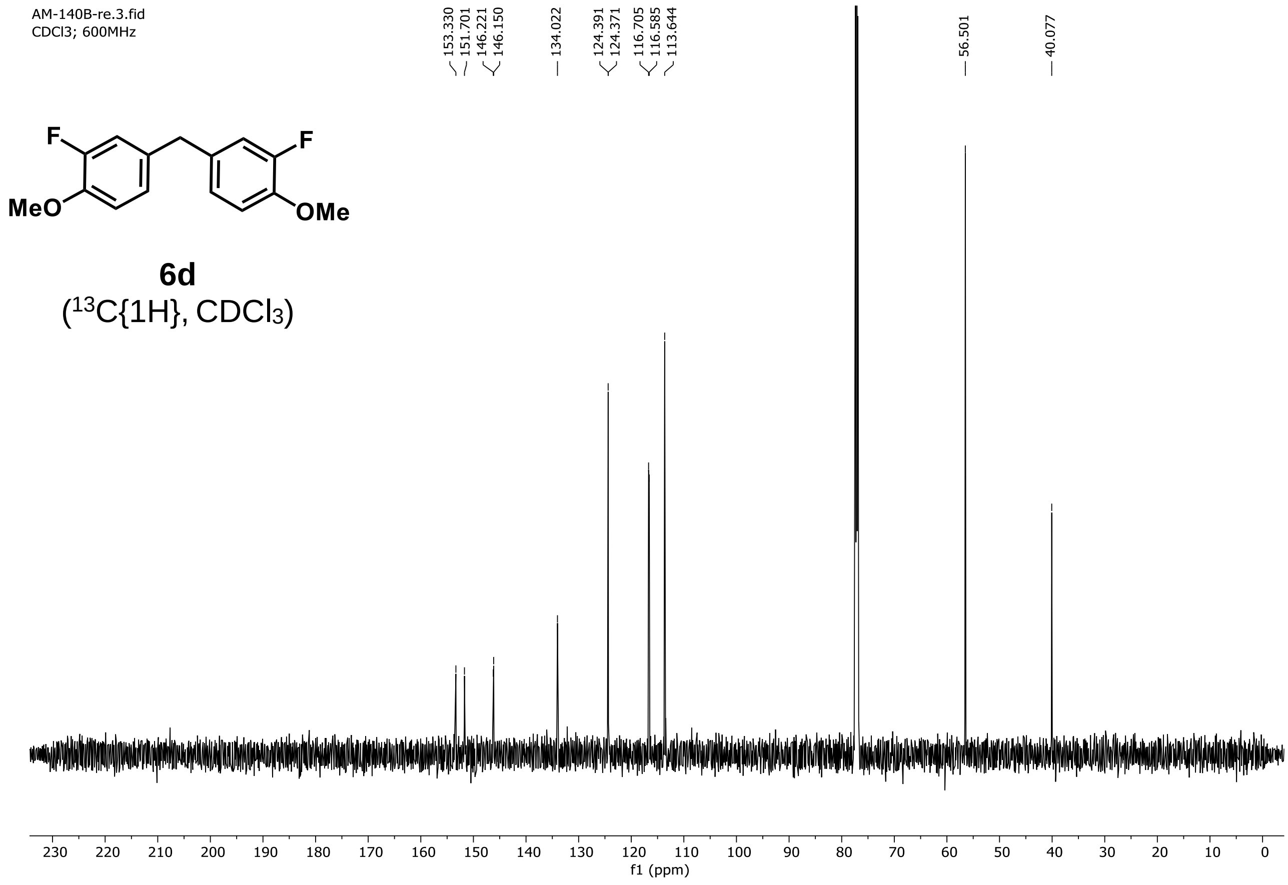
AM-140B-re.3.fid
CDCl3; 600MHz
153.330
151.701
146.221
146.150
134.022
124.391
124.371
116.705
116.585
113.644
56.501
40.077
F
F
MeO
OMe
6d
(13C{1H}, CDCl3)
230
220
210
200
190
180
170
160
150
140
130
120
110
100
90
80
70
60
50
40
30
20
10
0
f1 (ppm)

AM 140B.4.fid

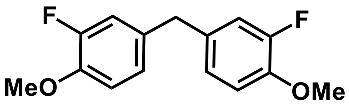

6d

(^{19}F, $CDCl_3$)

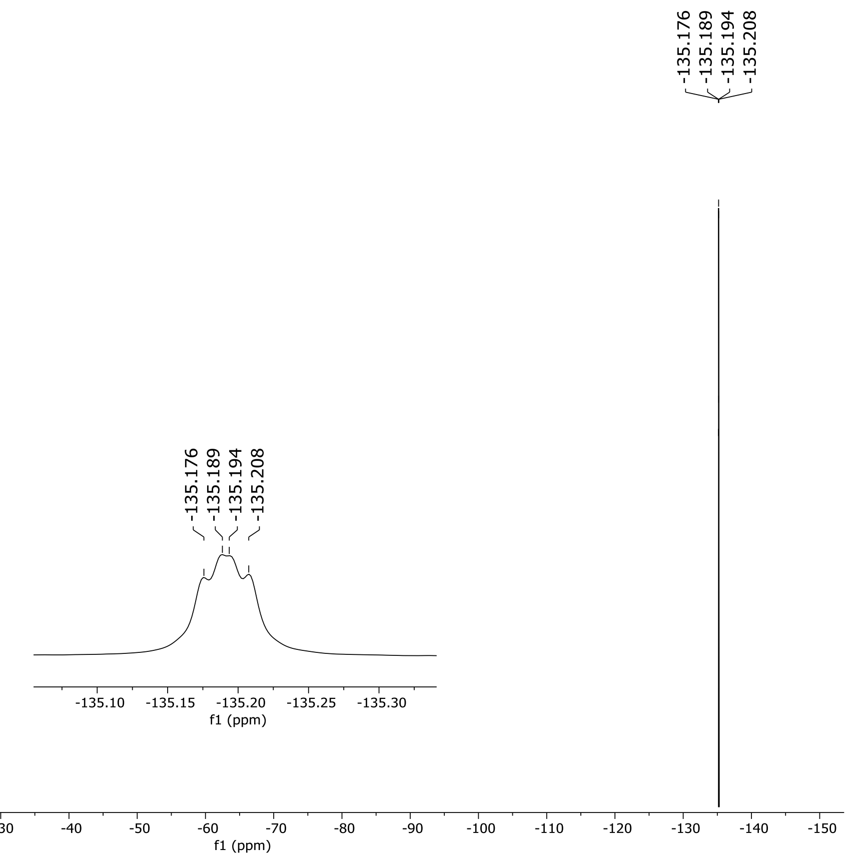

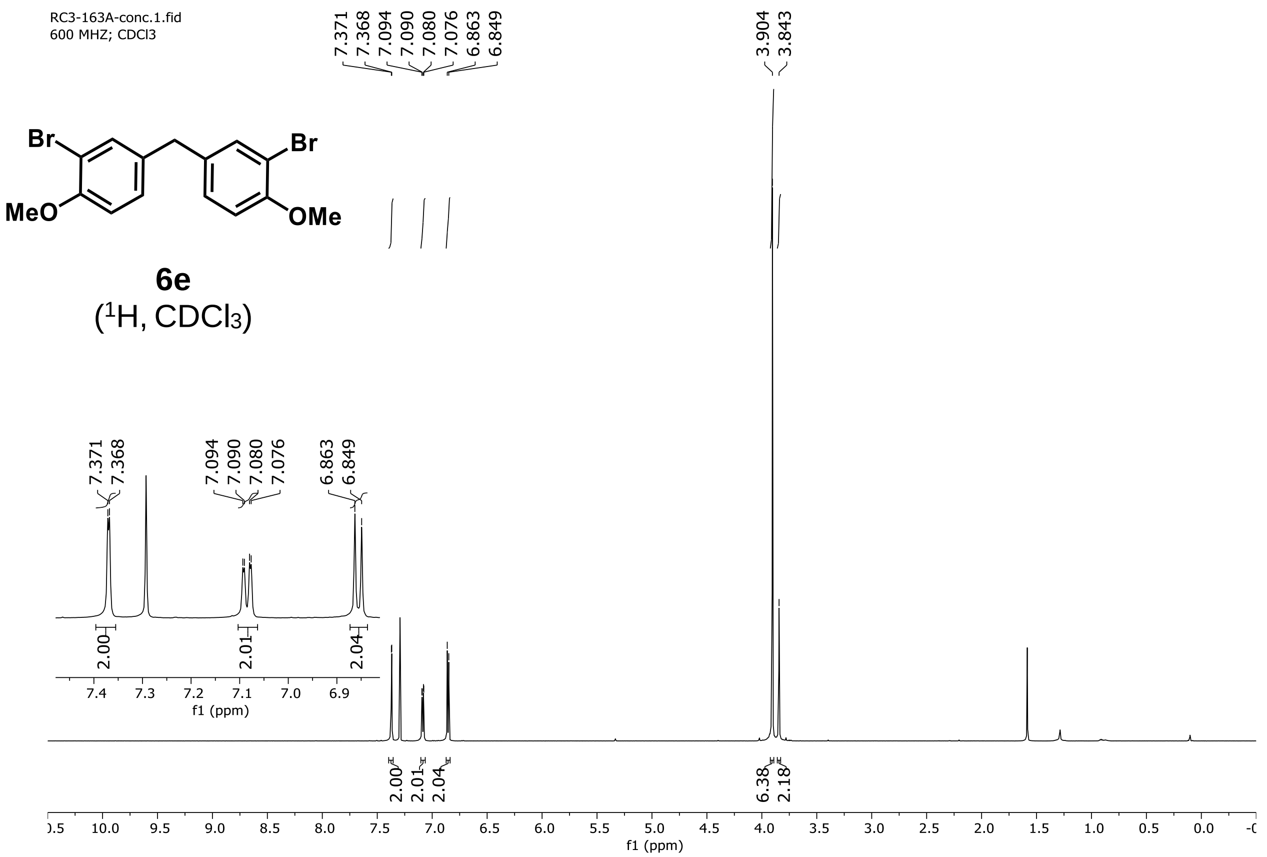
RC3-163A-conc.1.fid
600 MHZ; CDCl3
7.371
7.368
7.094
7.090
7.080
7.076
6.863
6.849
3.904
3.843
Br
Br
MeO
OMe
6e
(1H, CDCl3)
2.00
2.01
2.04
6.38
2.18
f1 (ppm)

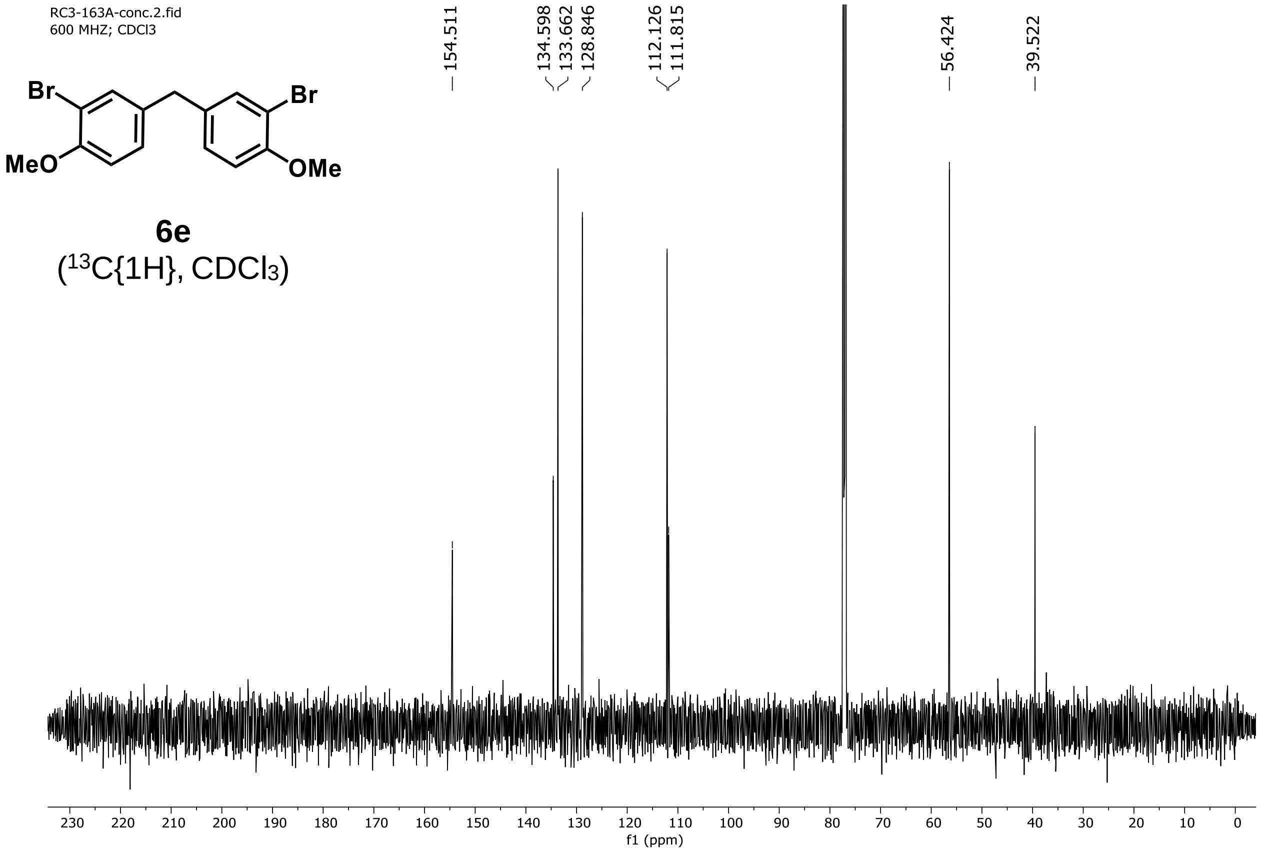
RC3-163A-conc.2.fid
600 MHZ; CDCl3
154.511
134.598
133.662
128.846
112.126
111.815
56.424
39.522
Br
Br
MeO
OMe
6e
(13C{1H}, CDCl3)
f1 (ppm)

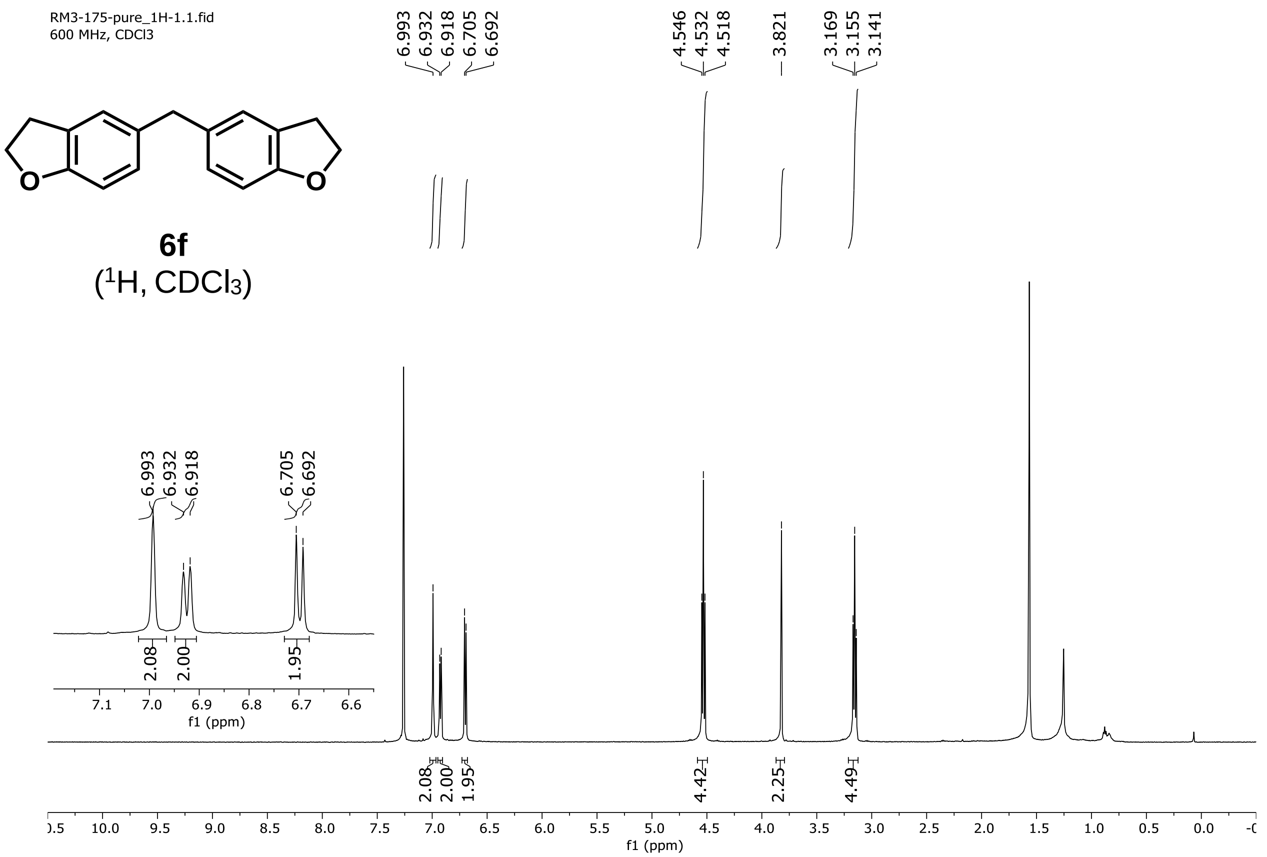
RM3-175-pure_1H-1.1.fid
600 MHz, CDCl3
6f
(1H, CDCl3)
6.993
6.932
6.918
6.705
6.692
4.546
4.532
4.518
3.821
3.169
3.155
3.141
2.08
2.00
1.95
4.42
2.25
4.49
f1 (ppm)

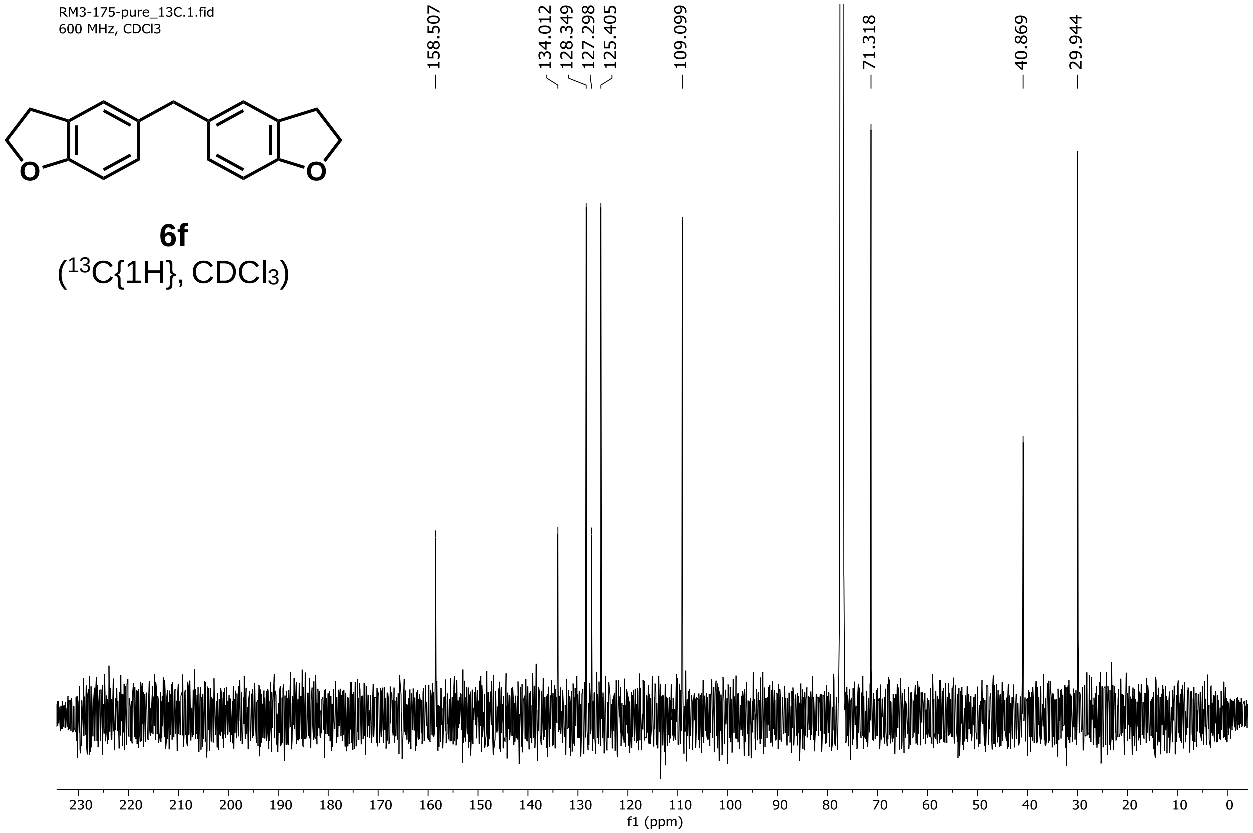
RM3-175-pure_13C.1.fid
600 MHz, CDCl3
6f
(13C{1H}, CDCl3)
158.507
134.012
128.349
127.298
125.405
109.099
71.318
40.869
29.944
f1 (ppm)

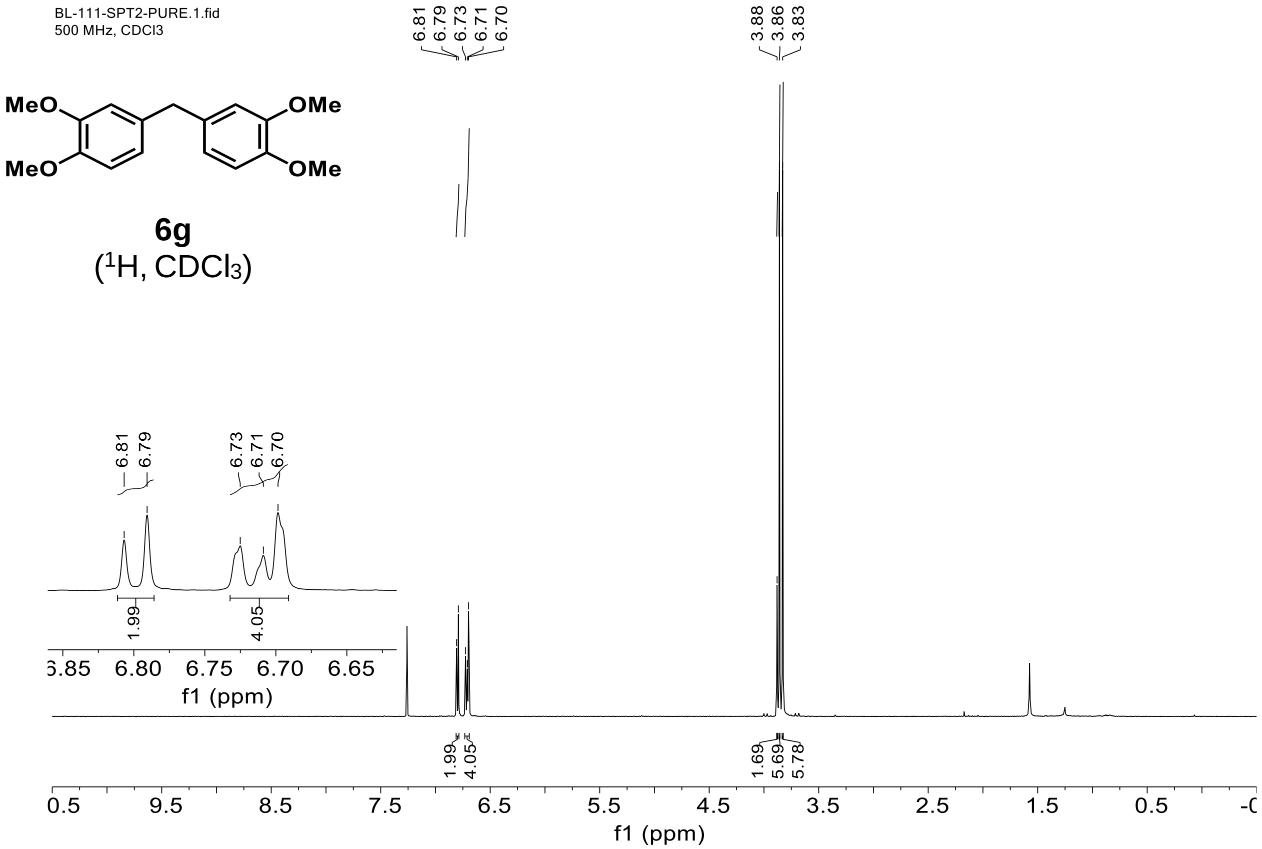
BL-111-SPT2-PURE.1.fid
500 MHz, CDCl3
MeO
MeO
OMe
OMe
6g
(¹H, CDCl3)
6.81 6.79 6.73 6.71 6.70
3.88 3.86 3.83
1.99 4.05
1.69 5.69 5.78
f1 (ppm)

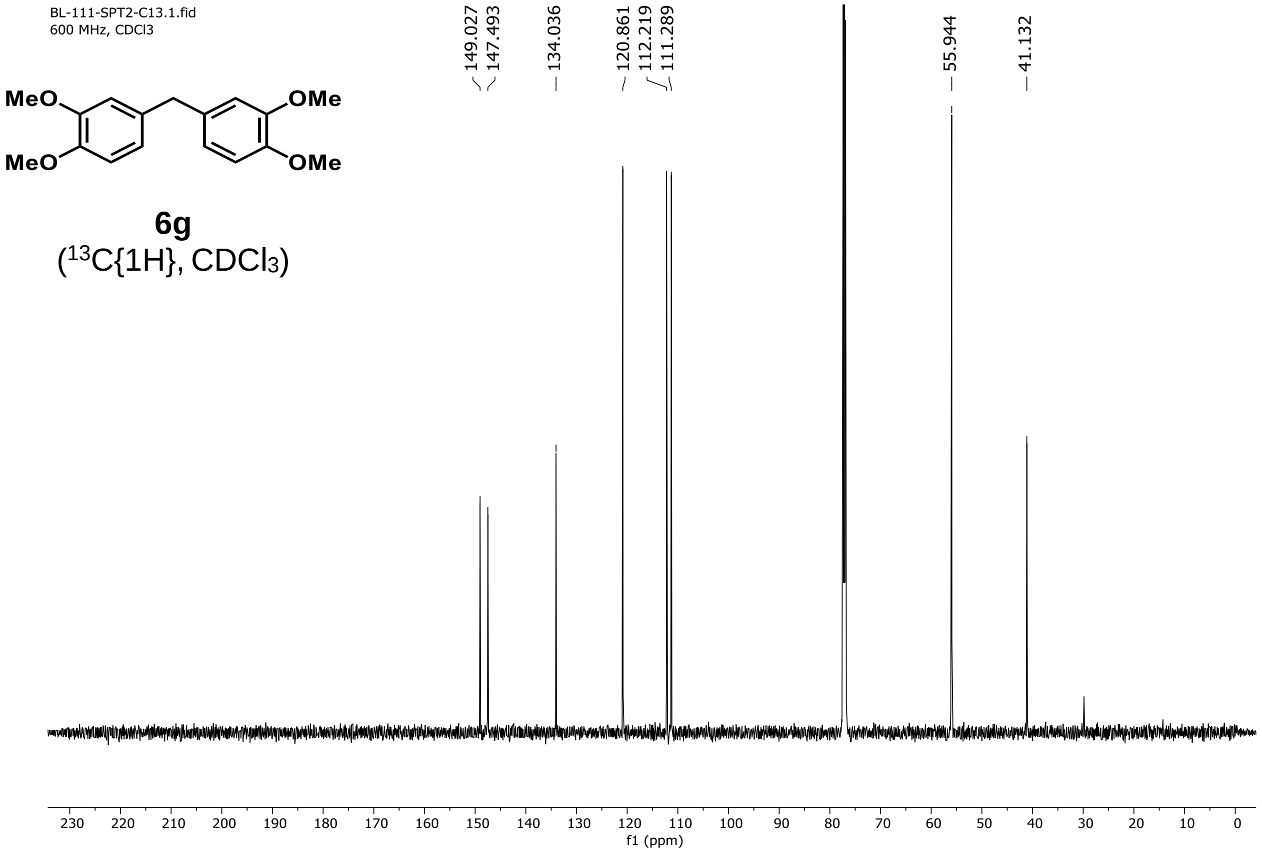
BL-111-SPT2-C13.1.fid
600 MHz, CDCl3
MeO
MeO
OMe
OMe
6g
(¹³C{1H}, CDCl3)
149.027 147.493 134.036 120.861 112.219 111.289 55.944 41.132
f1 (ppm)

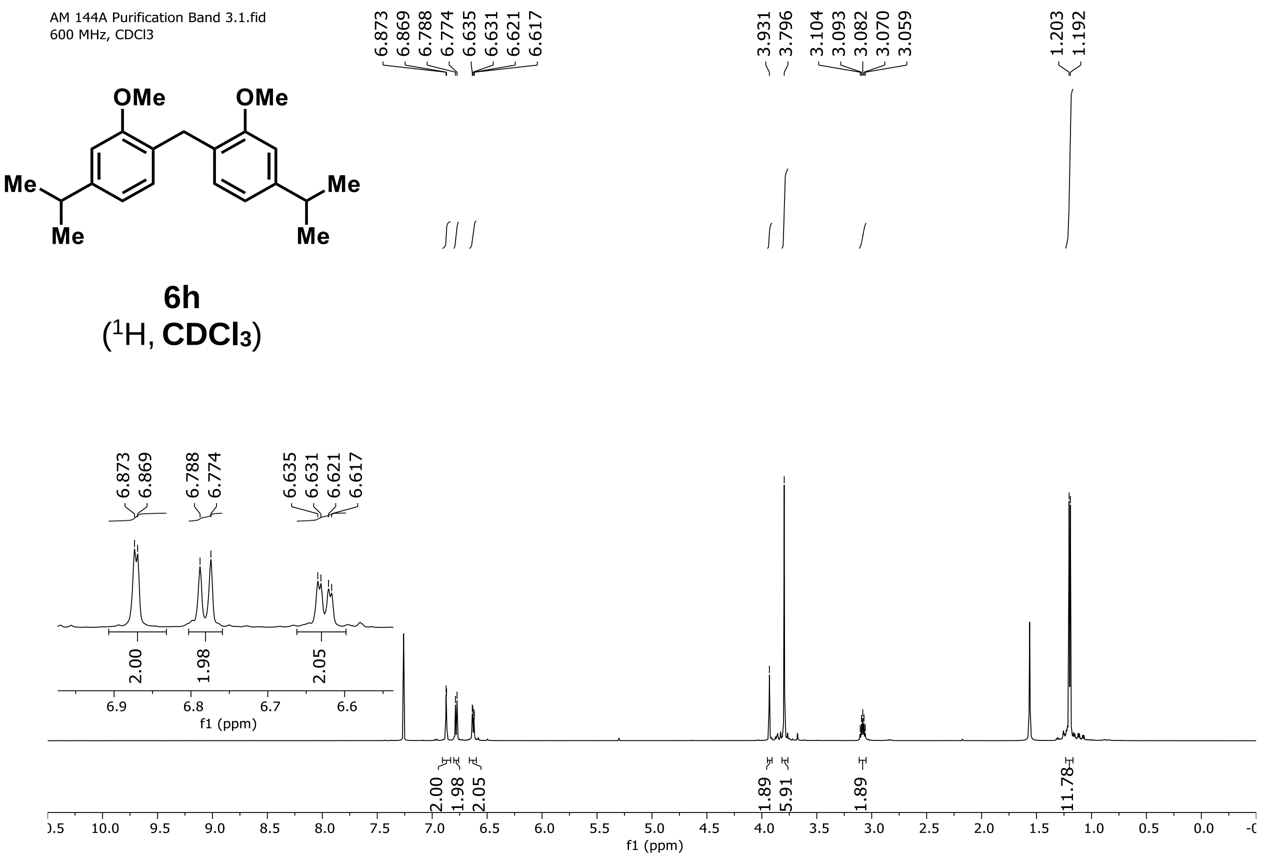
AM 144A Purification Band 3.1.fid
600 MHz, CDCl3
OMe
OMe
Me
Me
Me
Me
6h
(1H, CDCl3)
6.873
6.869
6.788
6.774
6.635
6.631
6.621
6.617
3.931
3.796
3.104
3.093
3.082
3.070
3.059
1.203
1.192
2.00
1.98
2.05
1.89
5.91
1.89
11.78
f1 (ppm)

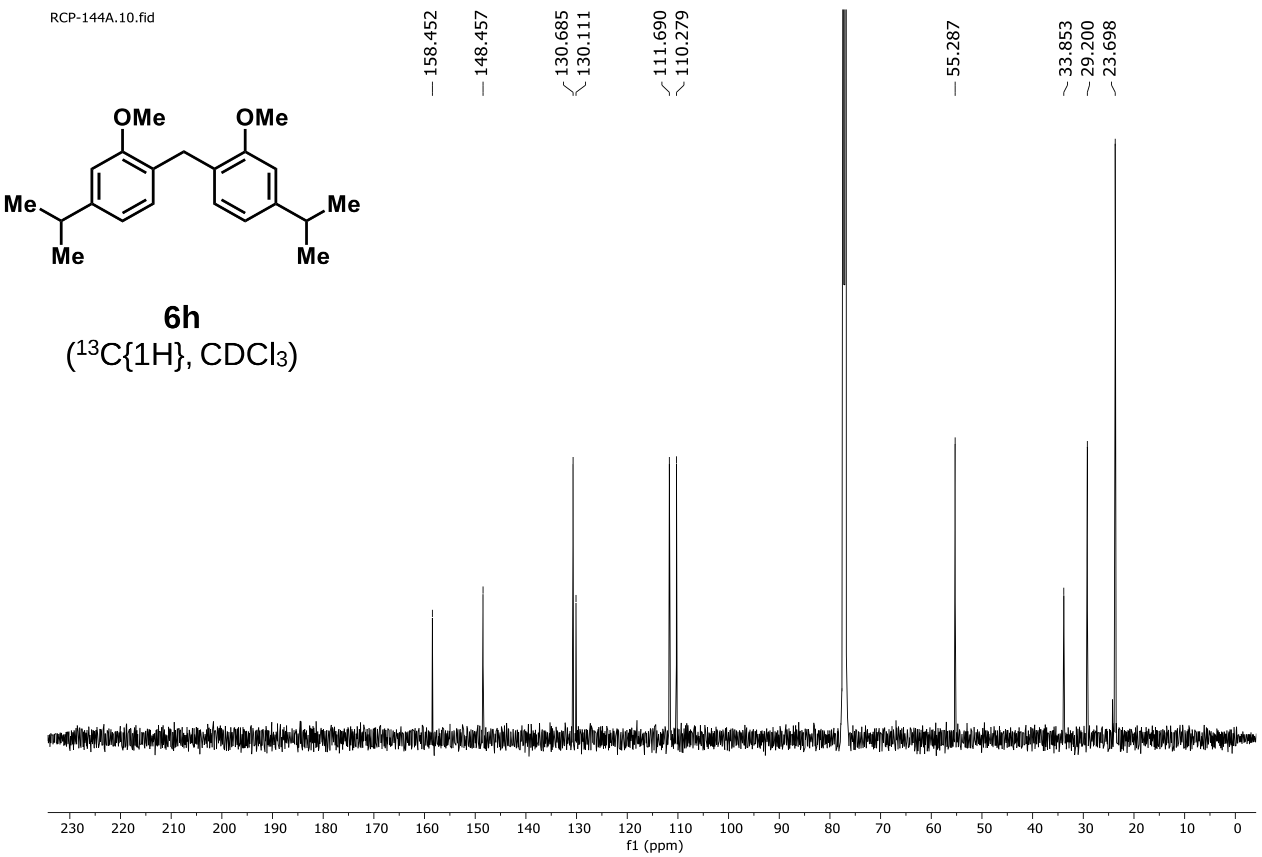
RCP-144A.10.fid
OMe
OMe
Me
Me
Me
Me
6h
(13C{1H}, CDCl3)
158.452
148.457
130.685
130.111
111.690
110.279
55.287
33.853
29.200
23.698
f1 (ppm)

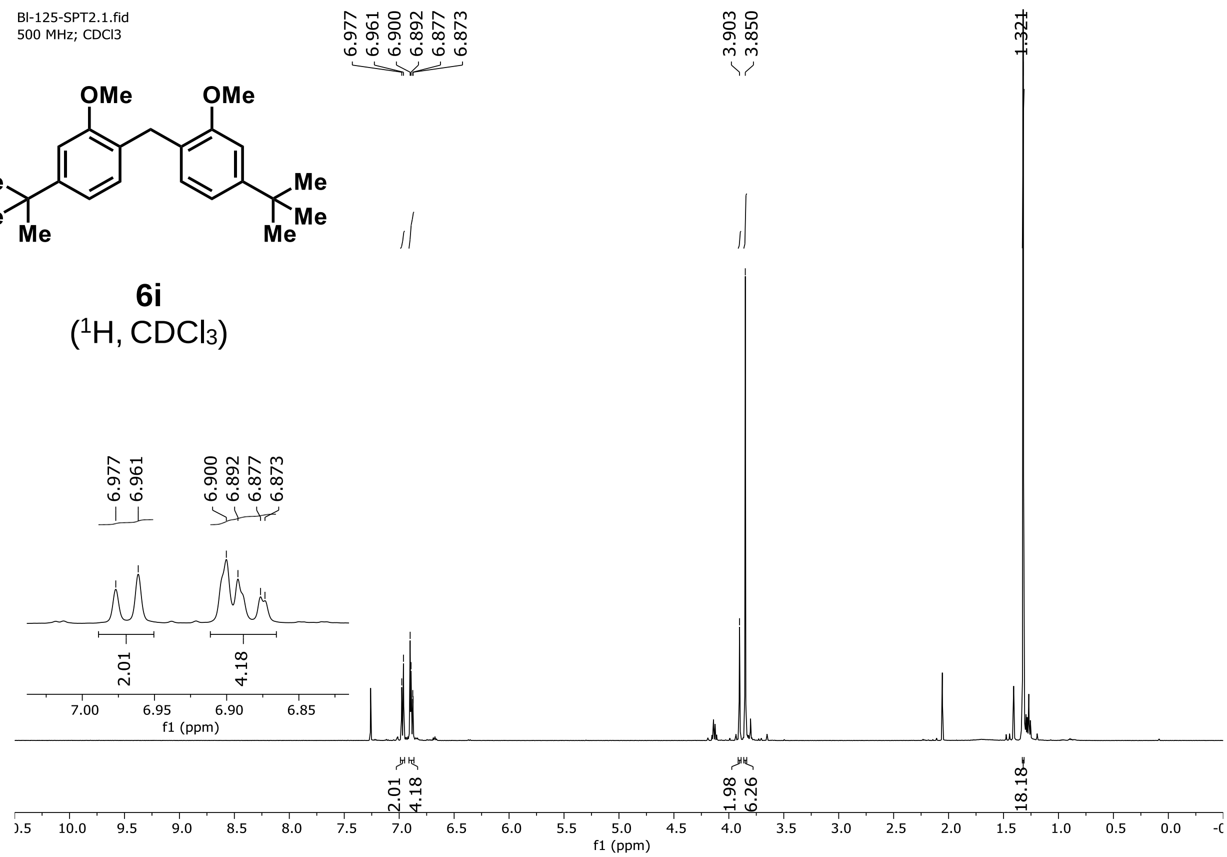

6i
(^{1}H, $CDCl_3$)

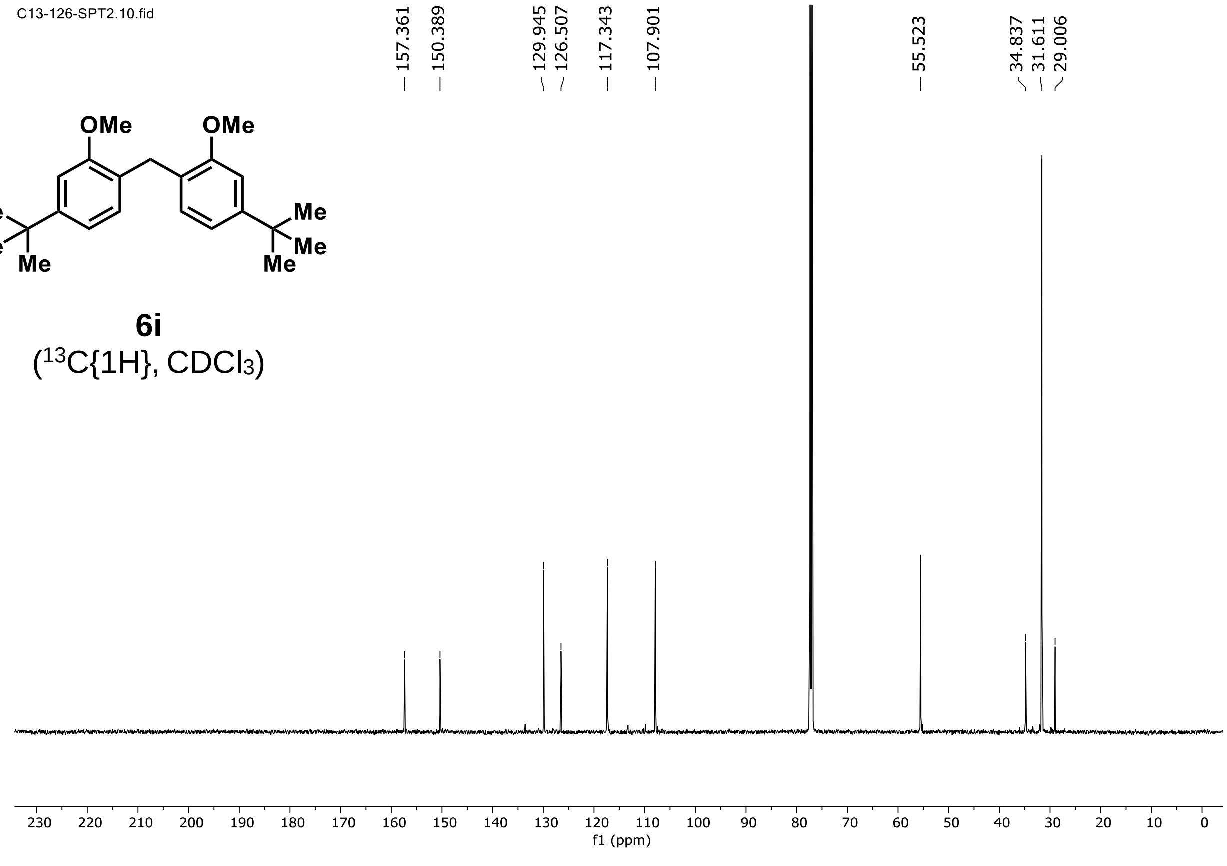

6i
(^{13}C{1H}, $CDCl_3$)

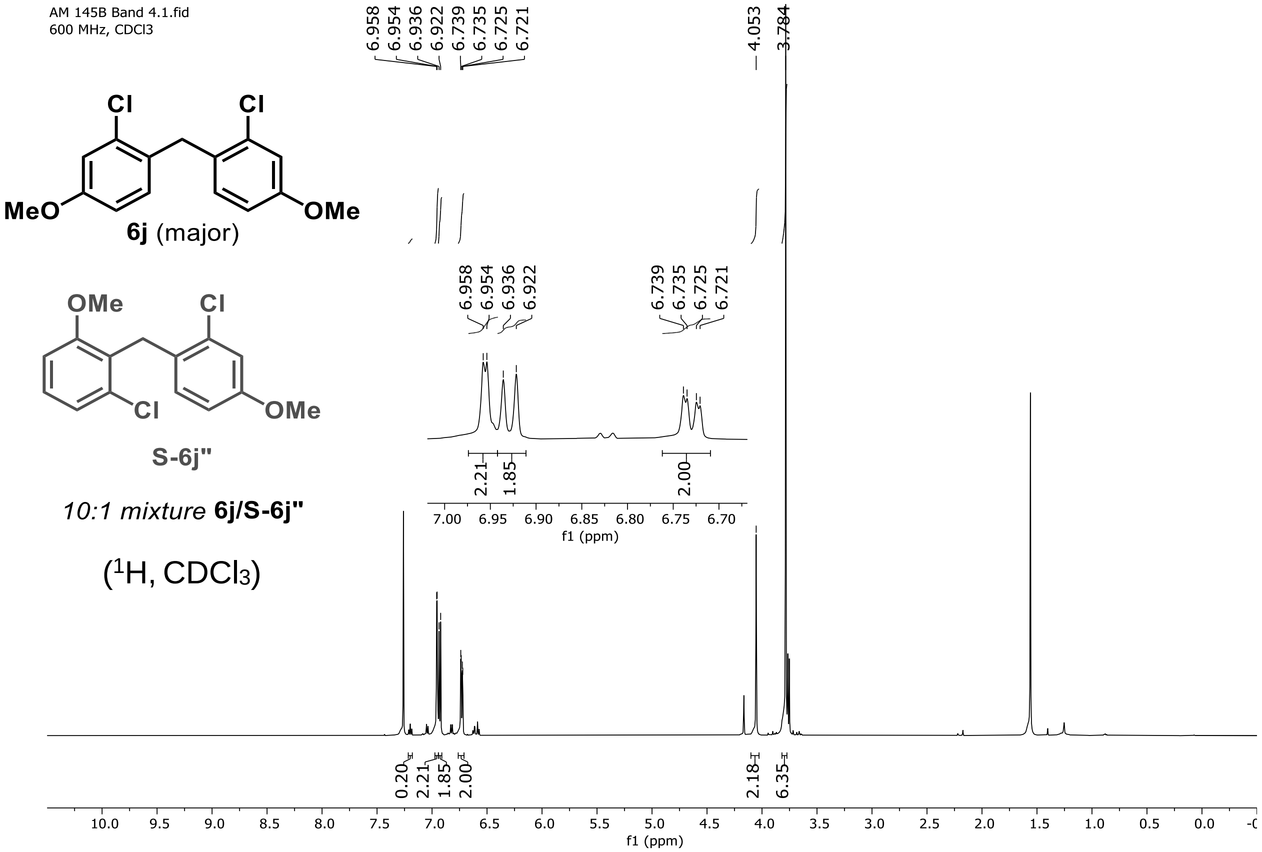

AM 145B Band 4.1.fid
600 MHz, CDCl3
6.958
6.954
6.936
6.922
6.739
6.735
6.725
6.721
4.053
3.784
Cl
Cl
MeO
OMe
6j (major)
OMe
Cl
Cl
OMe
S-6j"
10:1 mixture 6j/S-6j"
(1H, CDCl3)
2.21
1.85
2.00
f1 (ppm)
0.20
2.21
1.85
2.00
2.18
6.35
f1 (ppm)

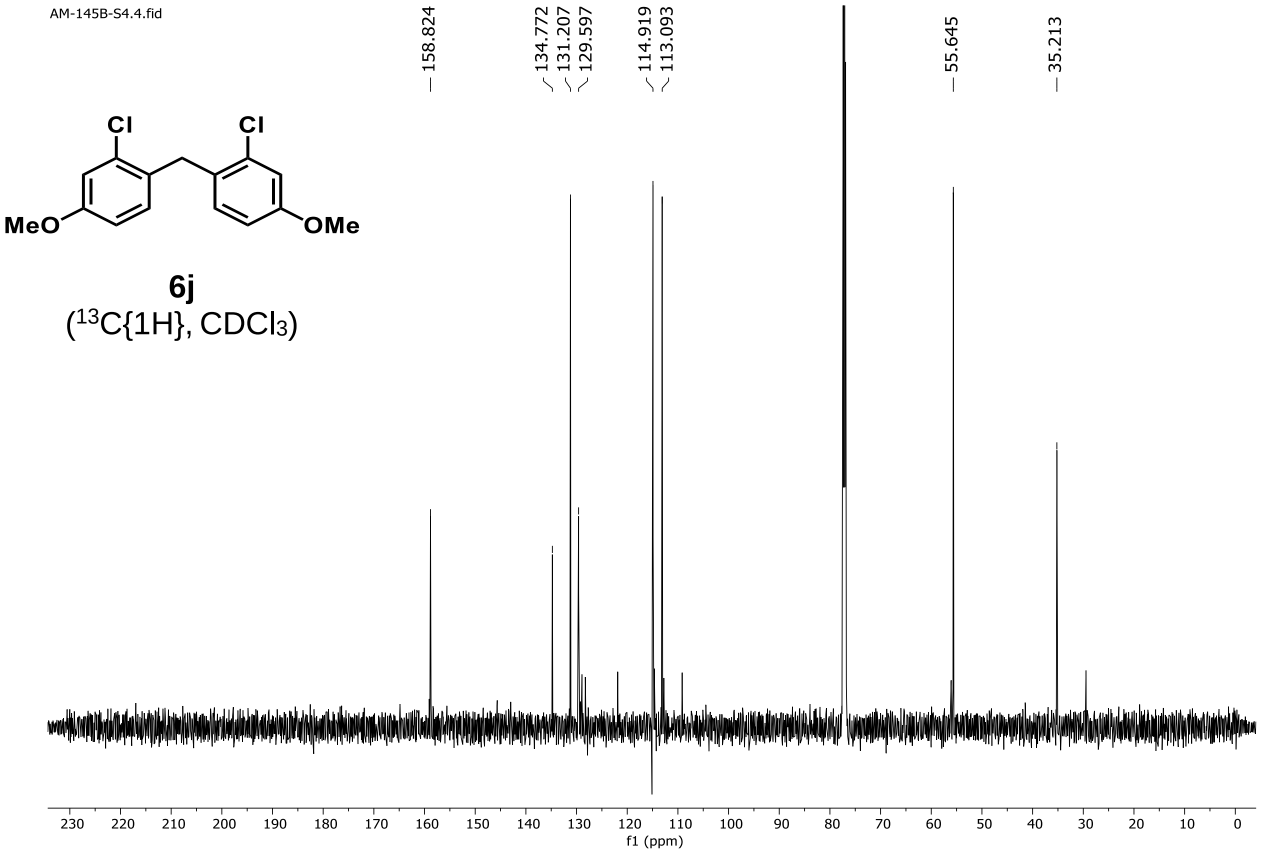

AM-145B-S4.4.fid
158.824
134.772
131.207
129.597
114.919
113.093
55.645
35.213
Cl
Cl
MeO
OMe
6j
(13C{1H}, CDCl3)
f1 (ppm)

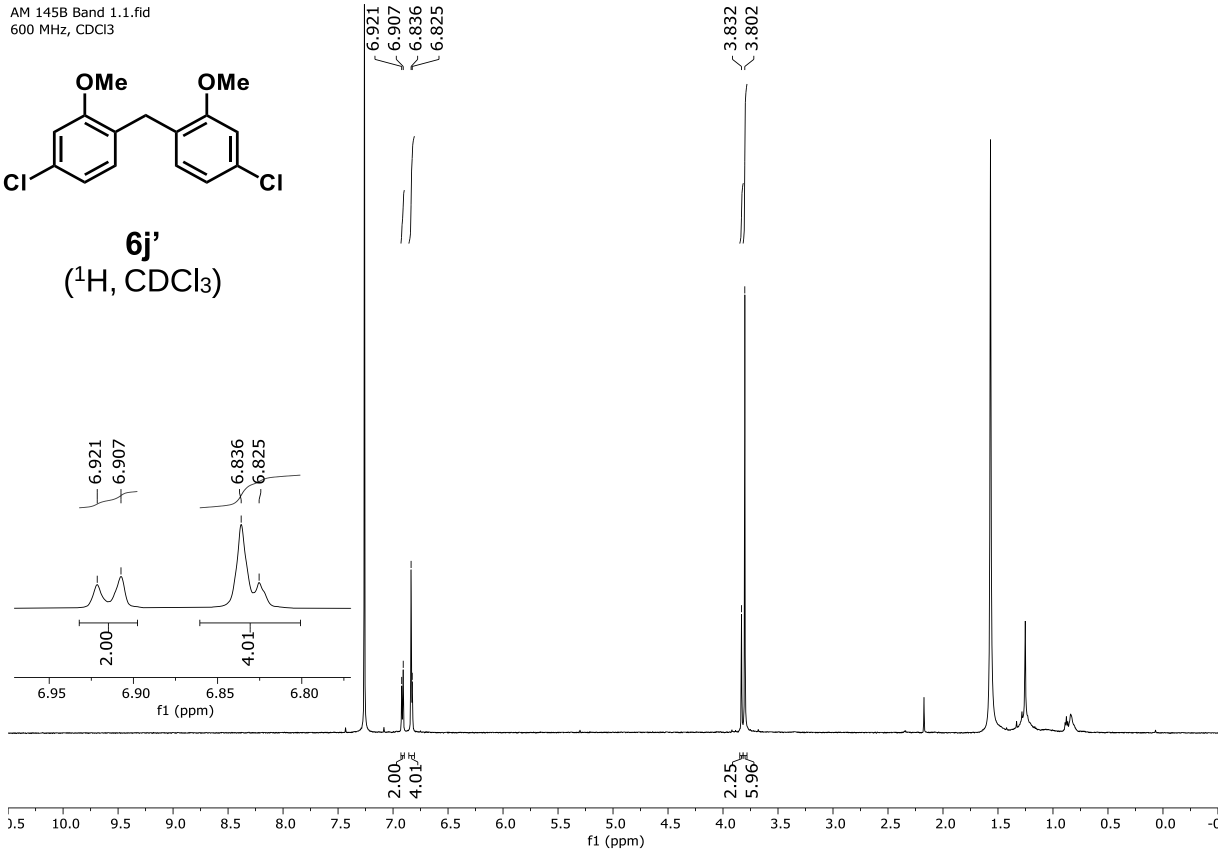
AM 145B Band 1.1.fid
600 MHz, CDCl3
OMe
OMe
Cl
Cl
6j’
(1H, CDCl3)
6.921
6.907
6.836
6.825
3.832
3.802
2.00
4.01
2.25
5.96
f1 (ppm)

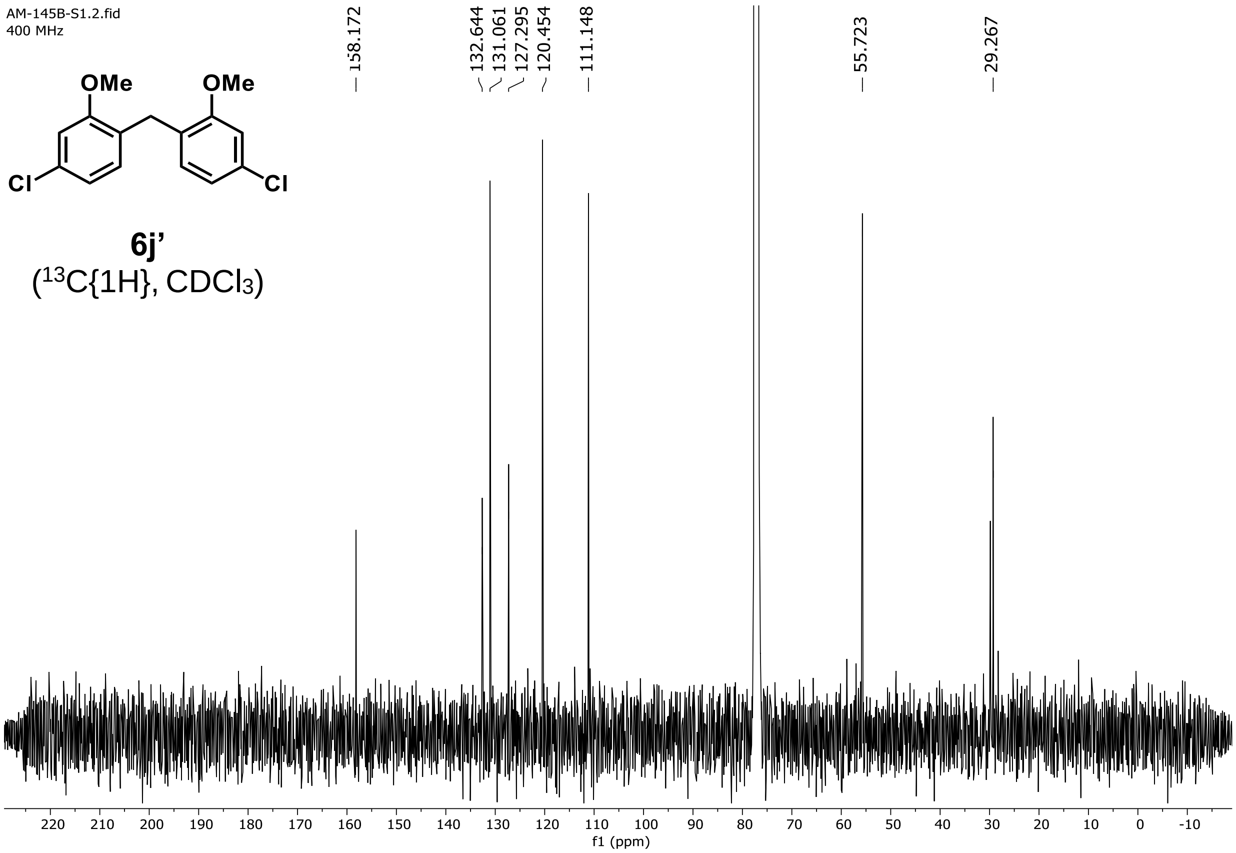
AM-145B-S1.2.fid
400 MHz
OMe
OMe
Cl
Cl
6j’
(13C{1H}, CDCl3)
158.172
132.644
131.061
127.295
120.454
111.148
55.723
29.267
f1 (ppm)

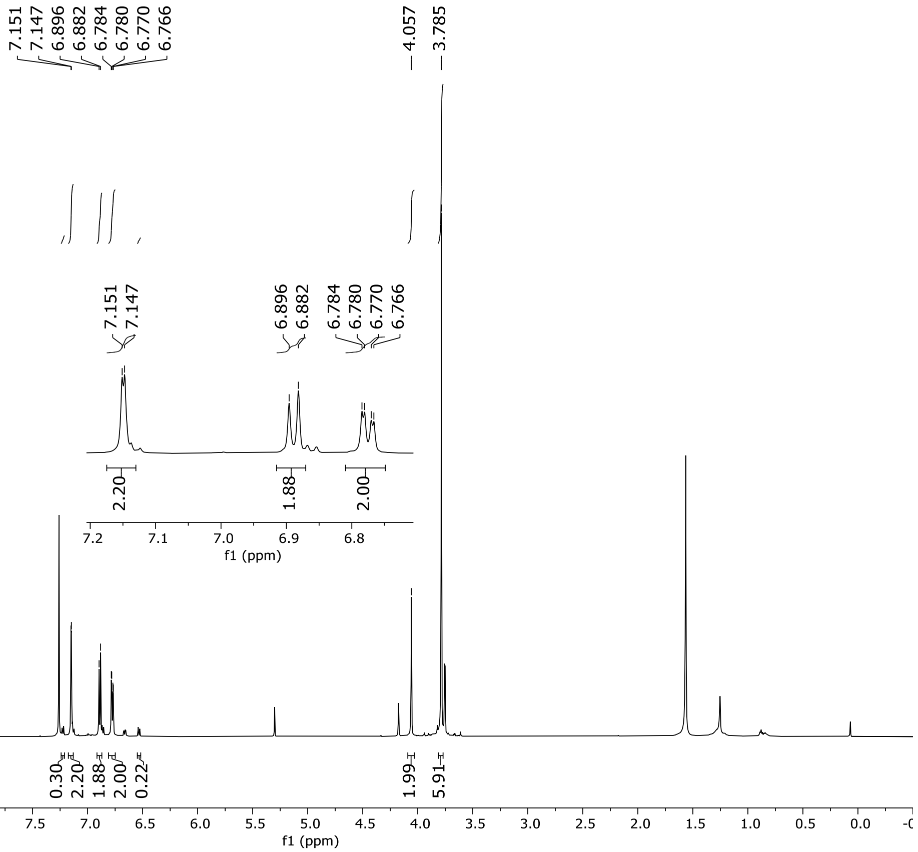

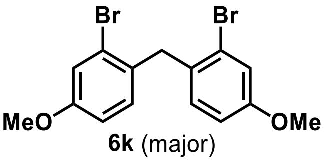

6k (major)

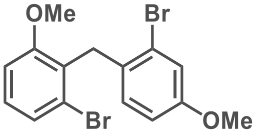

S-6k"

9:1 mixture **6k/6k"**

(^{1}H, $CDCl_3$)

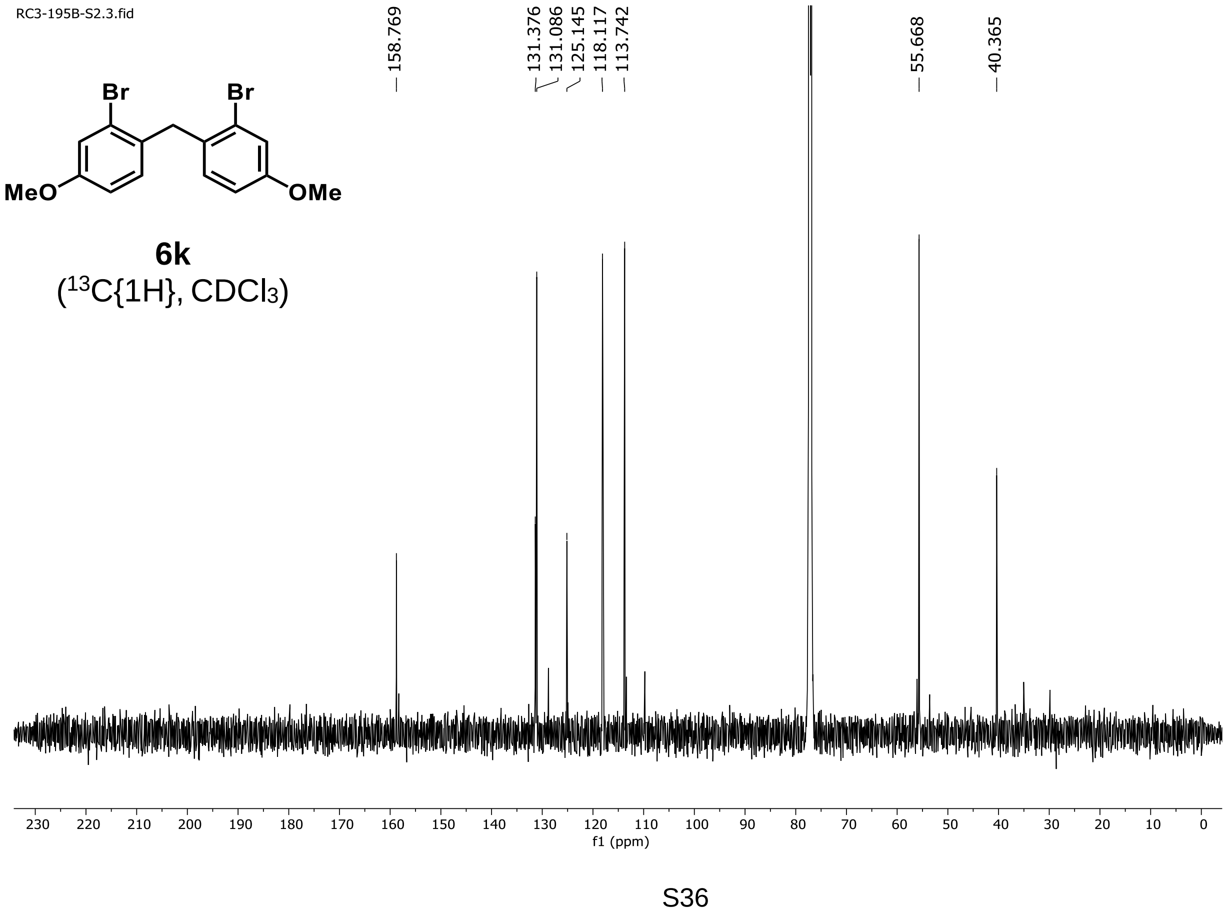

6k

(^{13}C{1H}, $CDCl_3$)

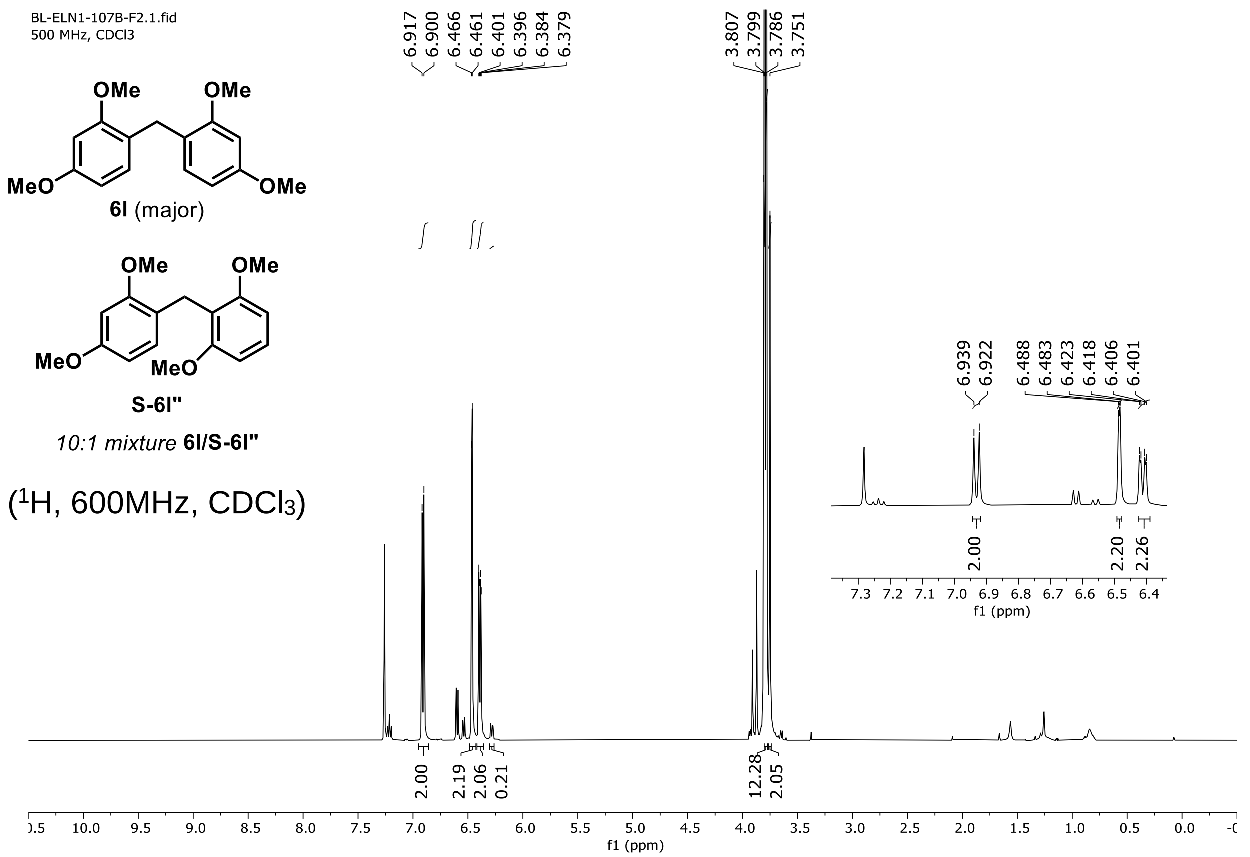
BL-ELN1-107B-F2.1.fid
500 MHz, CDCl3
6.917
6.900
6.466
6.461
6.401
6.396
6.384
6.379
3.807
3.799
3.786
3.751
OMe
OMe
MeO
OMe
6l (major)
OMe
OMe
MeO
MeO
S-6l"
10:1 mixture 6l/S-6l"
(1H, 600MHz, CDCl3)
6.939
6.922
6.488
6.483
6.423
6.418
6.406
6.401
2.00
2.20
2.26
f1 (ppm)
2.00
2.19
2.06
0.21
12.28
2.05
f1 (ppm)

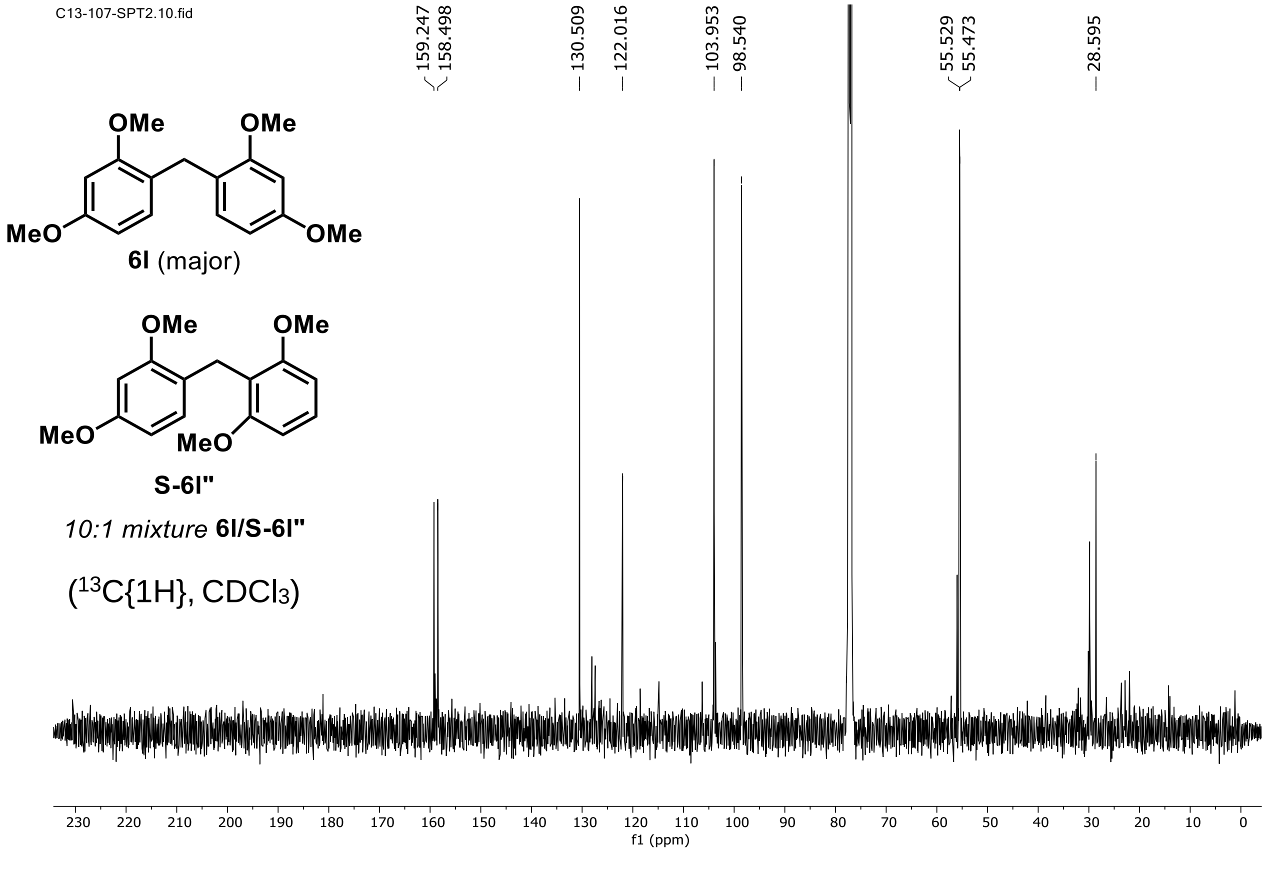
C13-107-SPT2.10.fid
159.247
158.498
130.509
122.016
103.953
98.540
55.529
55.473
28.595
OMe
OMe
MeO
OMe
6l (major)
OMe
OMe
MeO
MeO
S-6l"
10:1 mixture 6l/S-6l"
(13C{1H}, CDCl3)
f1 (ppm)

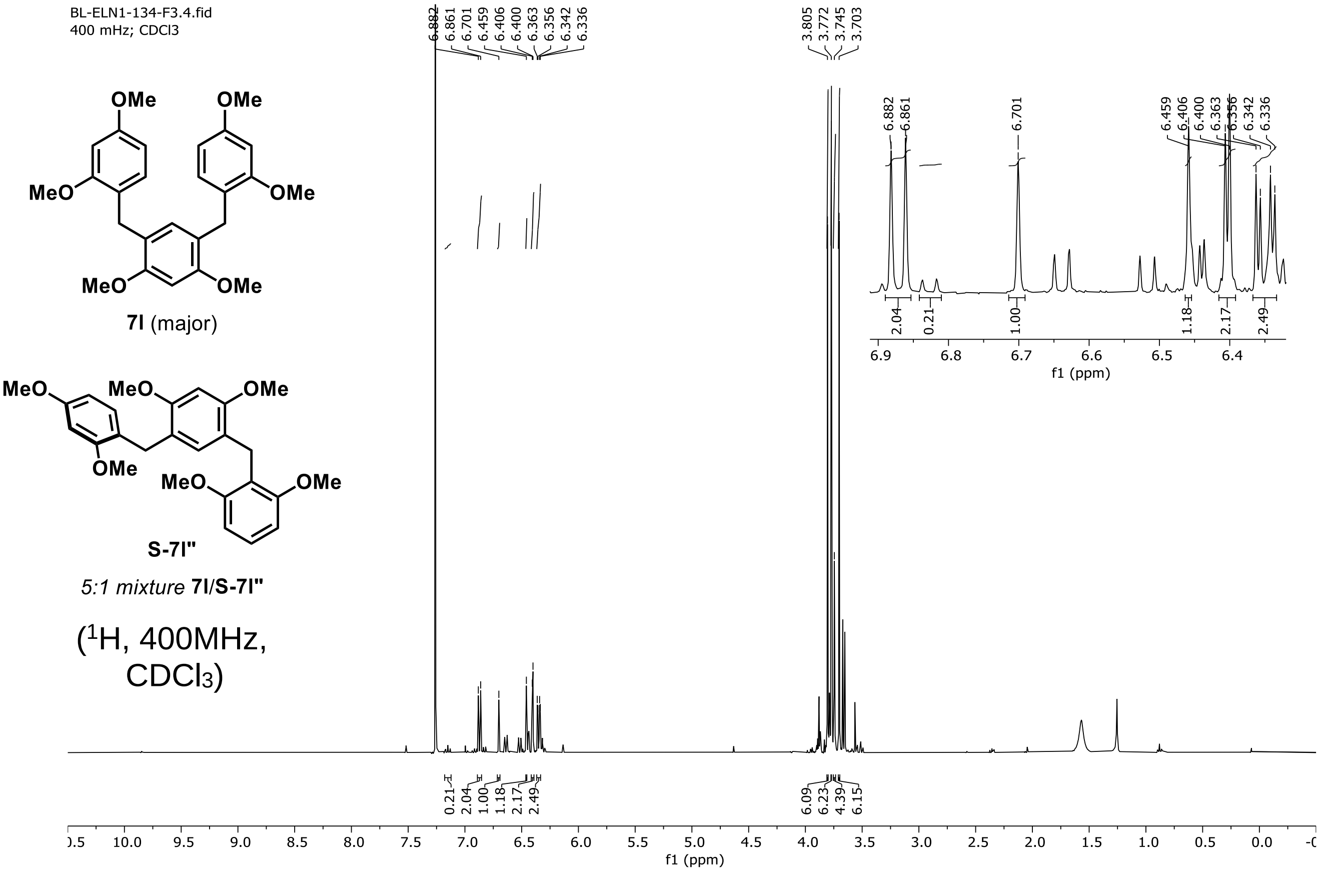
BL-ELN1-134-F3.4.fid
400 mHz; CDCl3
7l (major)
S-7l"
5:1 mixture 7l/S-7l"
(1H, 400MHz, CDCl3)
6.882
6.861
6.701
6.459
6.406
6.400
6.363
6.356
6.342
6.336
3.805
3.772
3.745
3.703
f1 (ppm)

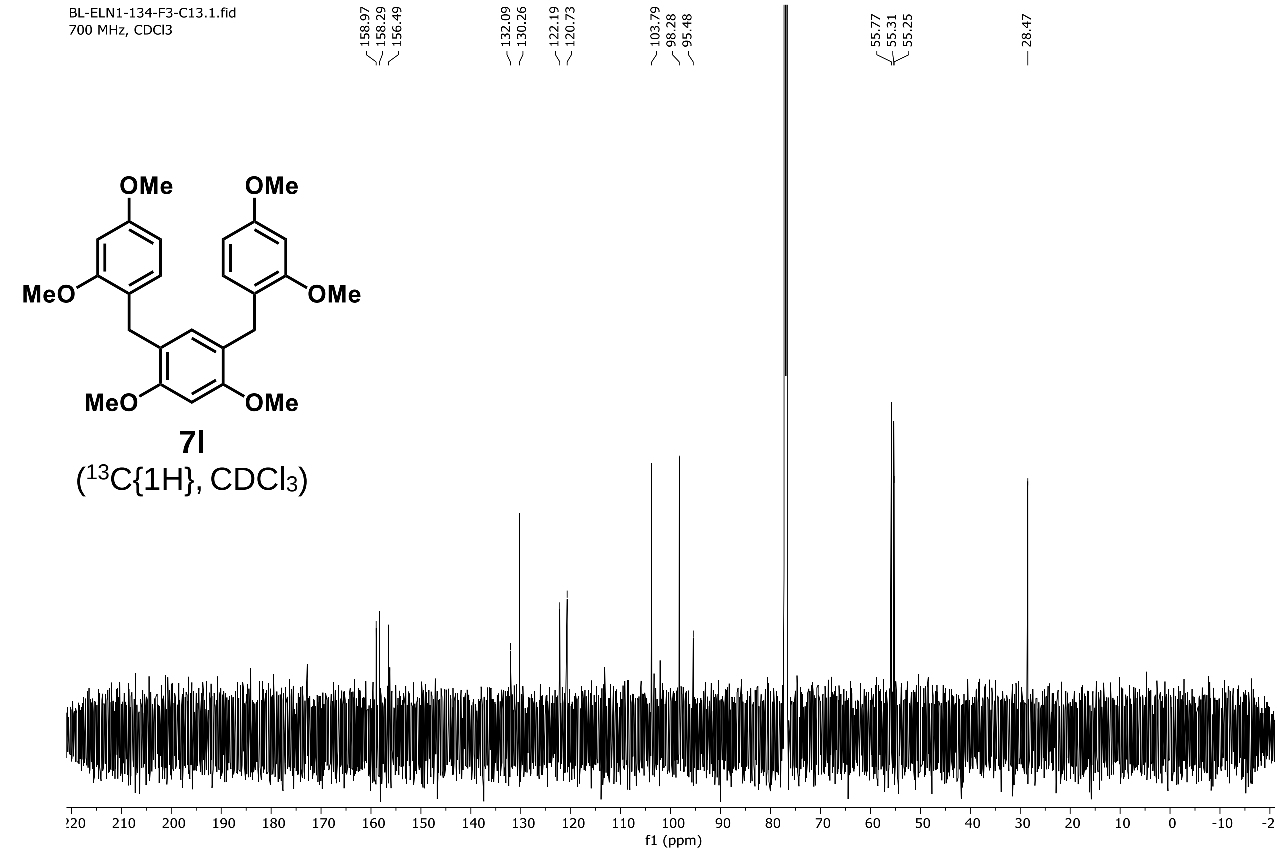
BL-ELN1-134-F3-C13.1.fid
700 MHz, CDCl3
158.97
158.29
156.49
132.09
130.26
122.19
120.73
103.79
98.28
95.48
55.77
55.31
55.25
28.47
7l
(13C{1H}, CDCl3)
f1 (ppm)

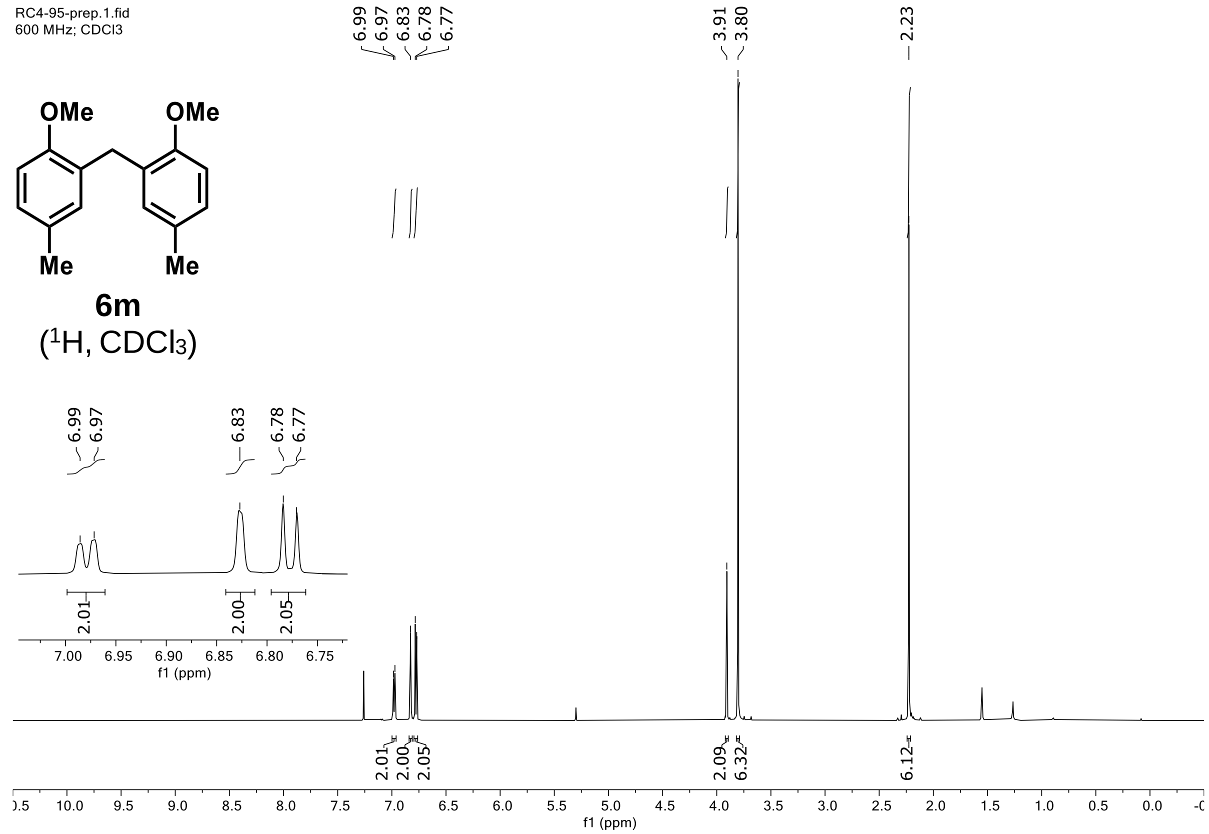
RC4-95-prep.1.fid
600 MHz; CDCl3
6.99
6.97
6.83
6.78
6.77
3.91
3.80
2.23
OMe
OMe
Me
Me
6m
(1H, CDCl3)
2.01
2.00
2.05
2.09
6.32
6.12
f1 (ppm)

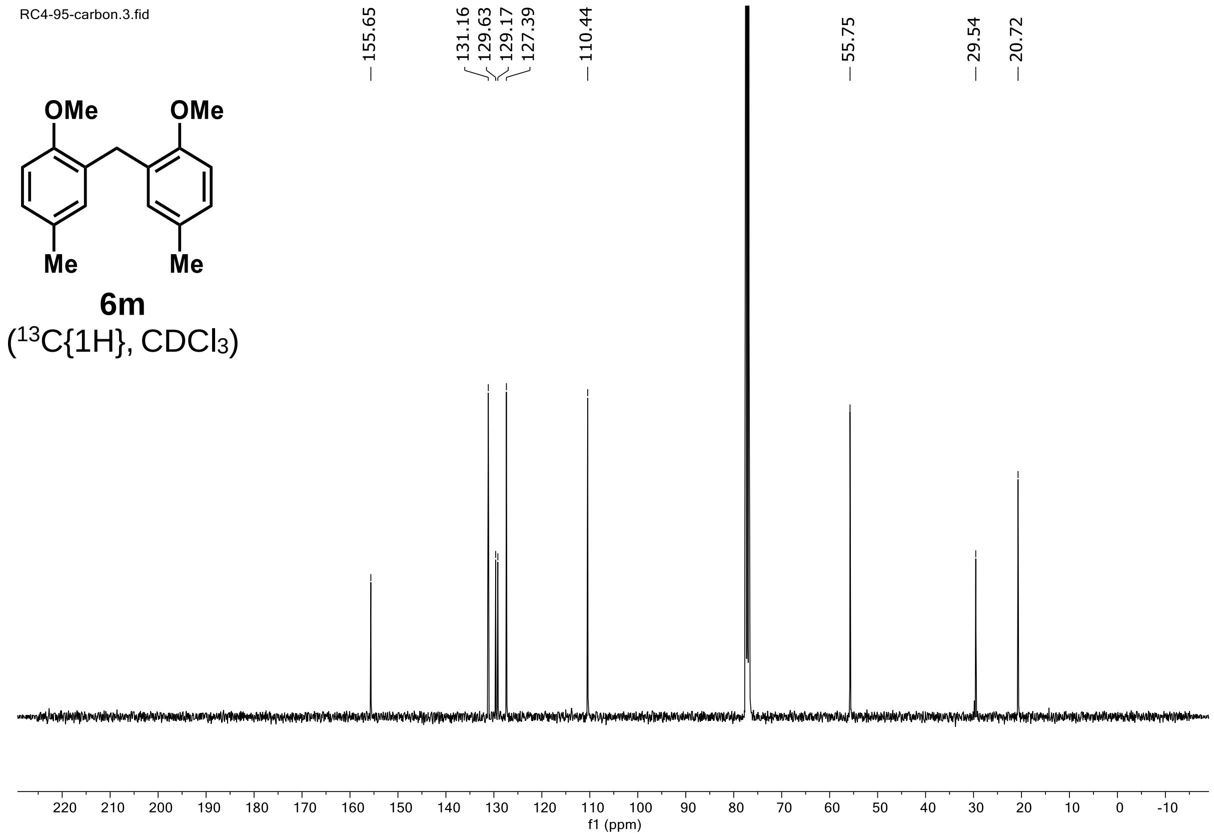
RC4-95-carbon.3.fid
155.65
131.16
129.63
129.17
127.39
110.44
55.75
29.54
20.72
OMe
OMe
Me
Me
6m
(13C{1H}, CDCl3)
f1 (ppm)

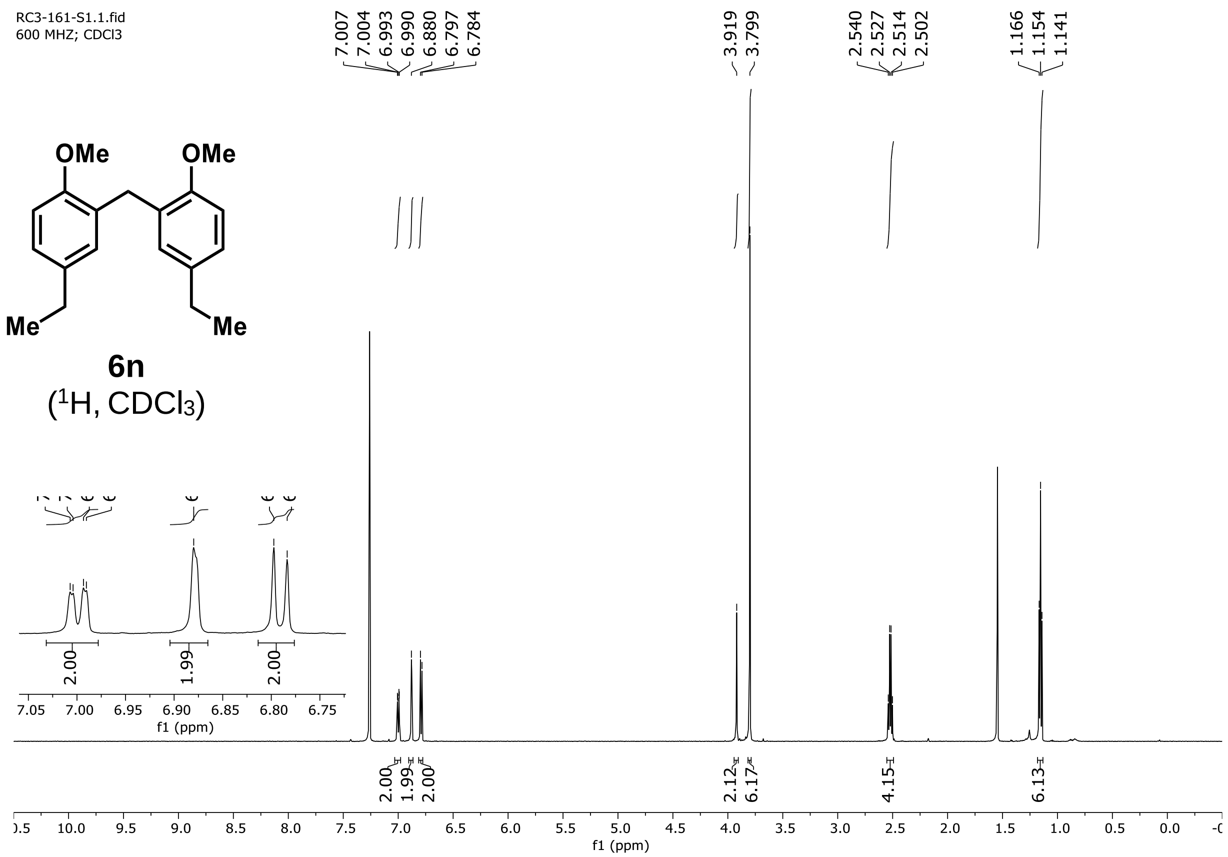

6n
(^{1}H, $CDCl_3$)

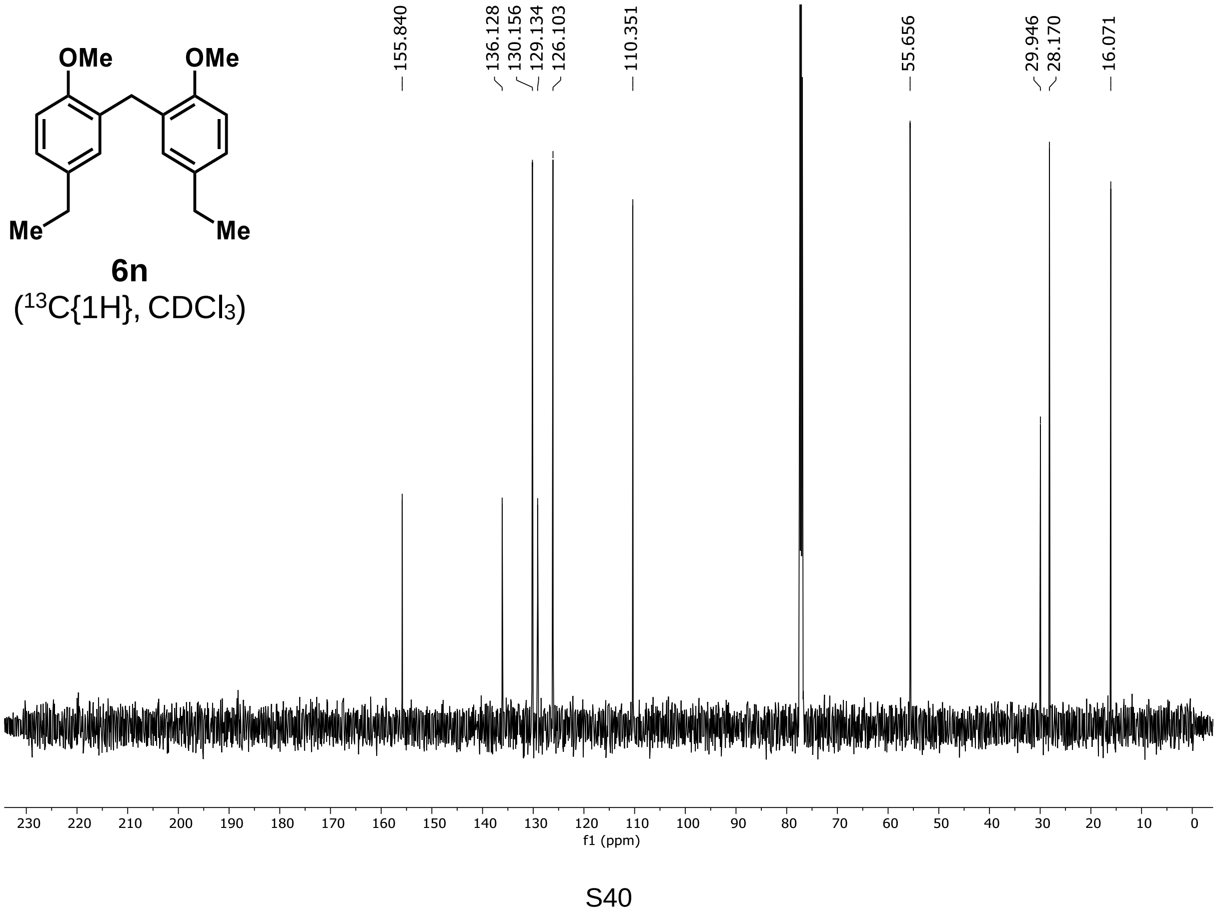

6n
(^{13}C{1H}, $CDCl_3$)

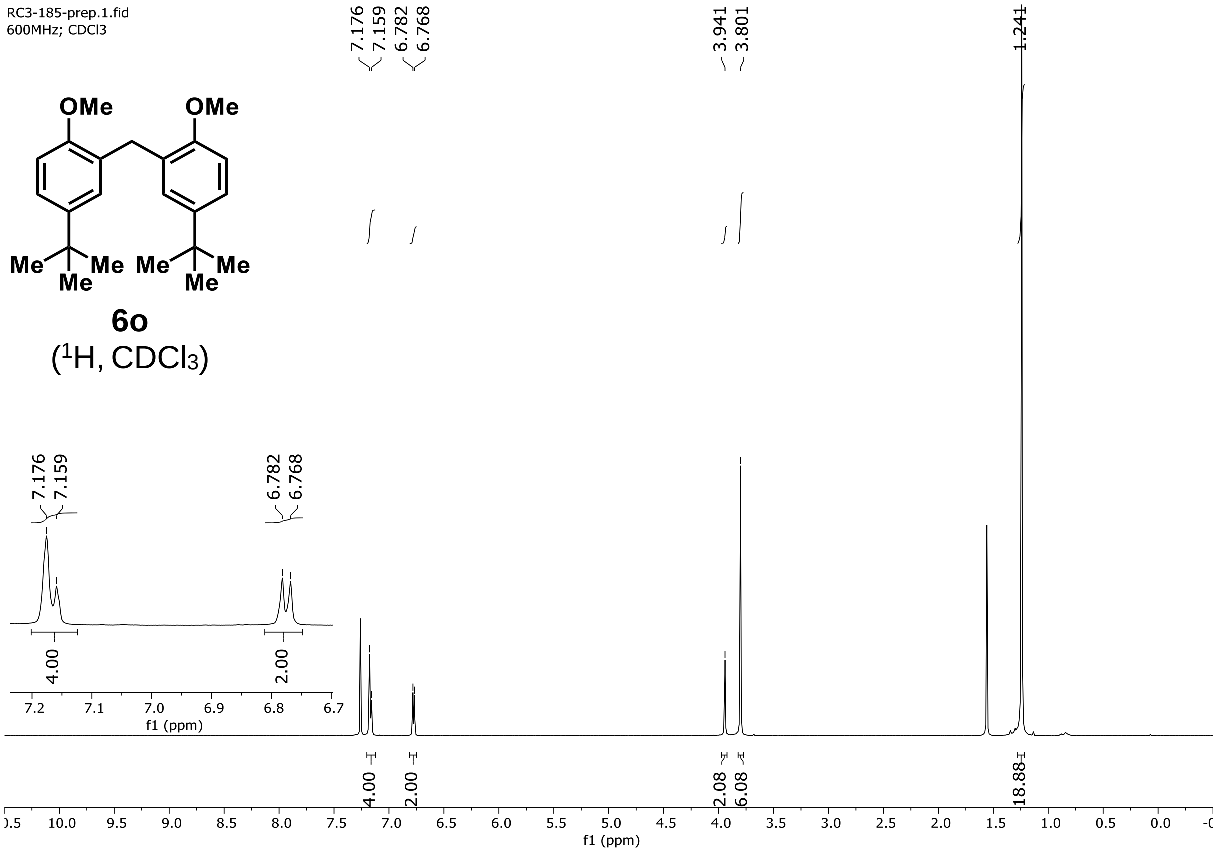

RC3-185-prep.1.fid
600MHz; CDCl3
OMe
OMe
Me
Me
Me
Me
Me
Me
6o
(1H, CDCl3)
7.176
7.159
6.782
6.768
3.941
3.801
1.241
4.00
2.00
2.08
6.08
18.88
f1 (ppm)

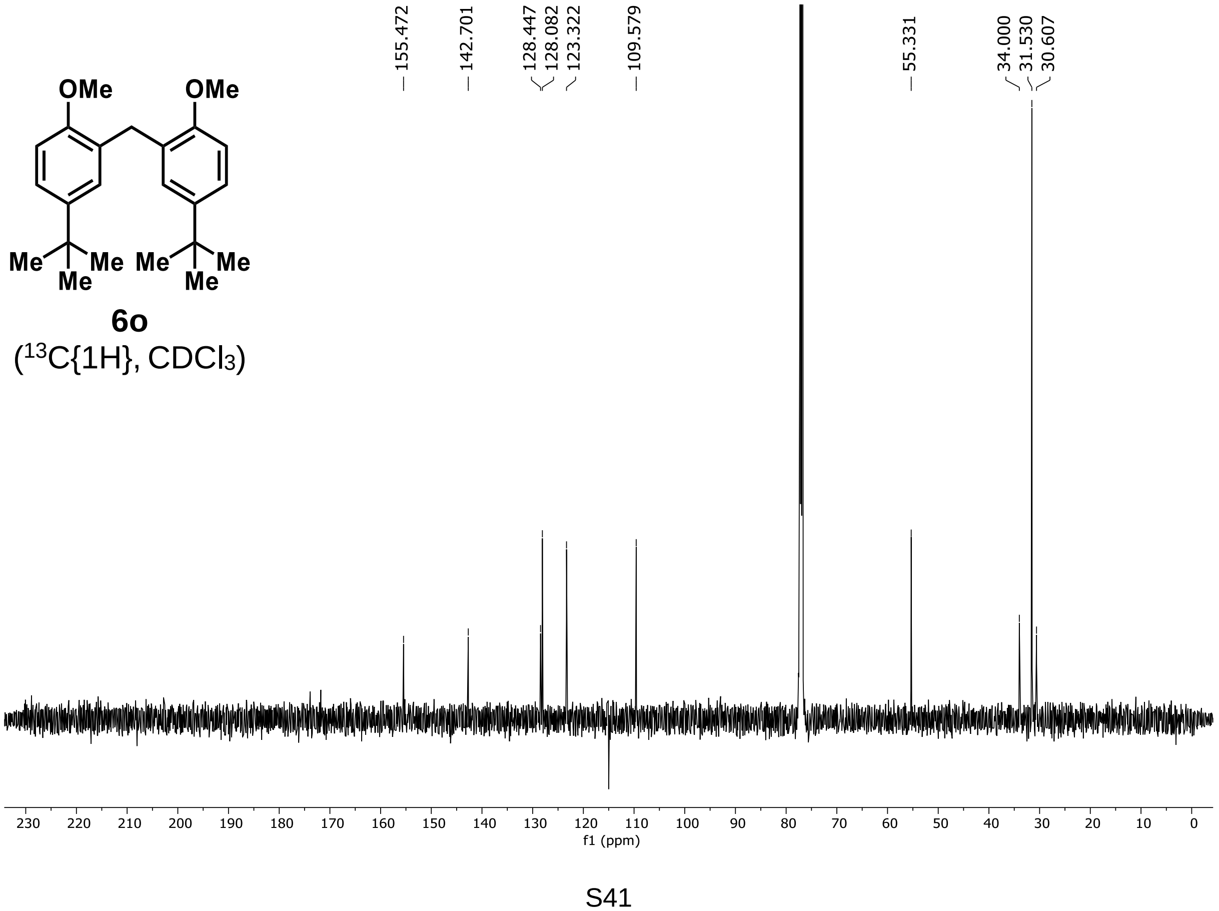

OMe
OMe
Me
Me
Me
Me
Me
Me
6o
(13C{1H}, CDCl3)
155.472
142.701
128.447
128.082
123.322
109.579
55.331
34.000
31.530
30.607
f1 (ppm)

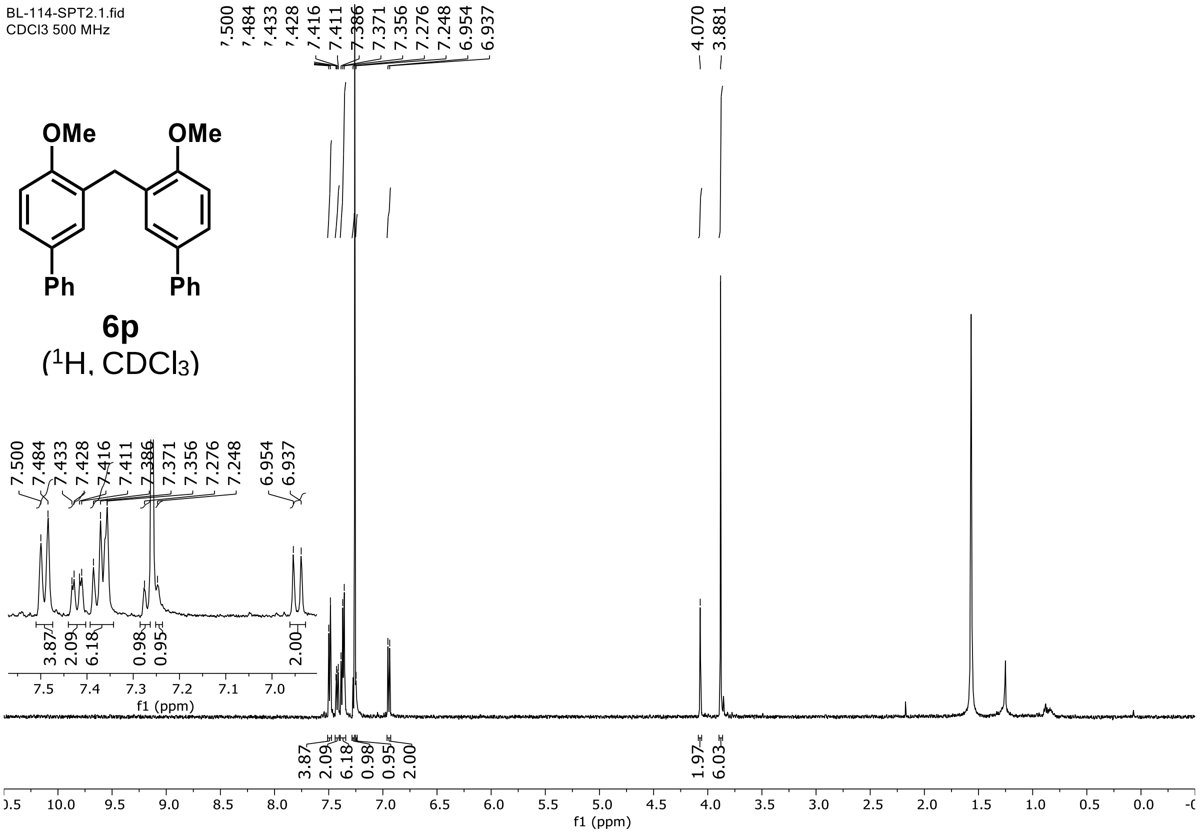
BL-114-SPT2.1.fid
CDCl3 500 MHz
7.500 7.484 7.433 7.428 7.416 7.411 7.386 7.371 7.356 7.276 7.248 6.954 6.937
4.070
3.881
OMe
OMe
Ph
Ph
6p
(1H, CDCl3)
3.87 2.09 6.18 0.98 0.95 2.00
1.97 6.03
f1 (ppm)

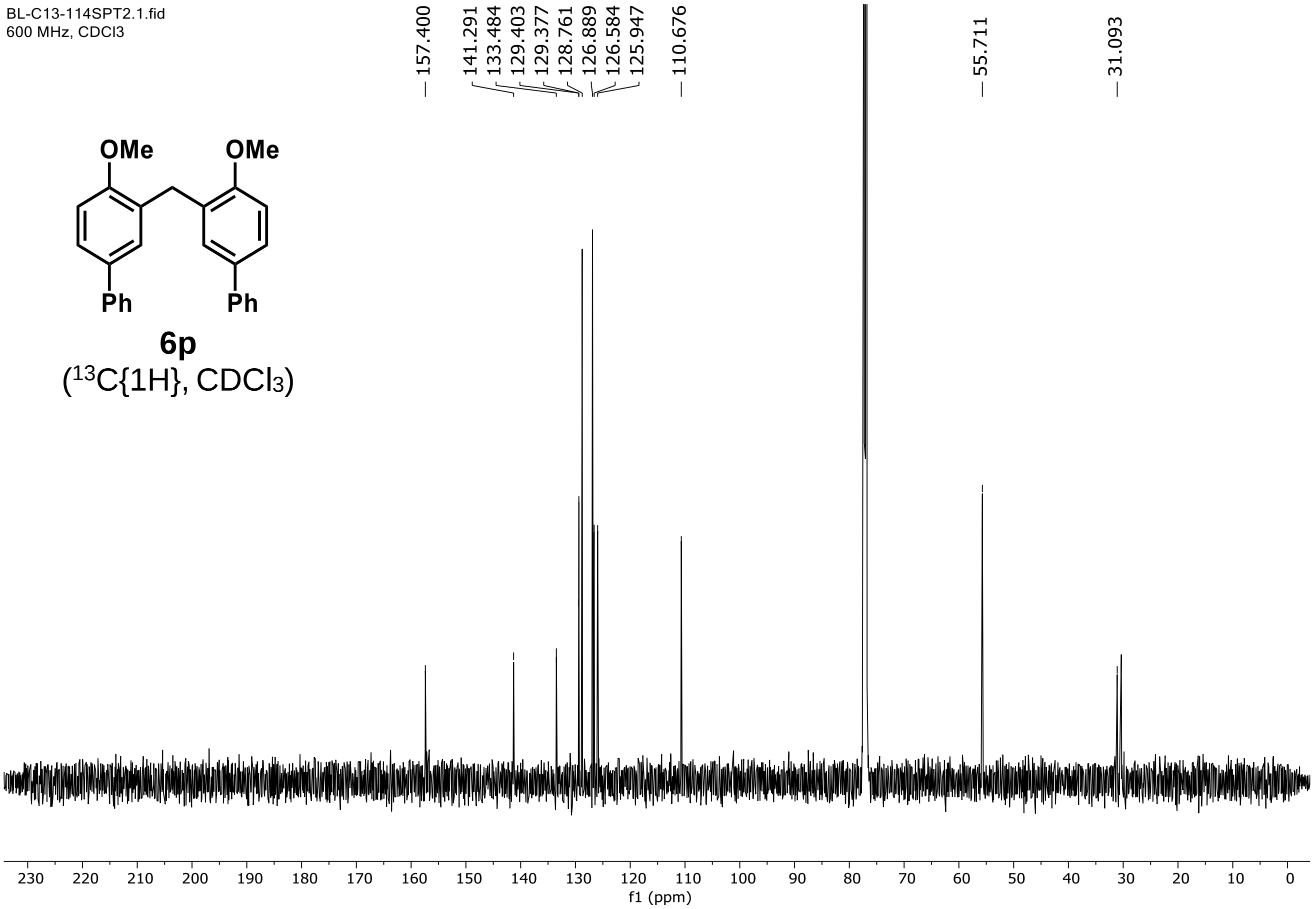
BL-C13-114SPT2.1.fid
600 MHz, CDCl3
157.400
141.291 133.484 129.403 129.377 128.761 126.889 126.584 125.947
110.676
55.711
31.093
OMe
OMe
Ph
Ph
6p
(13C{1H}, CDCl3)
f1 (ppm)

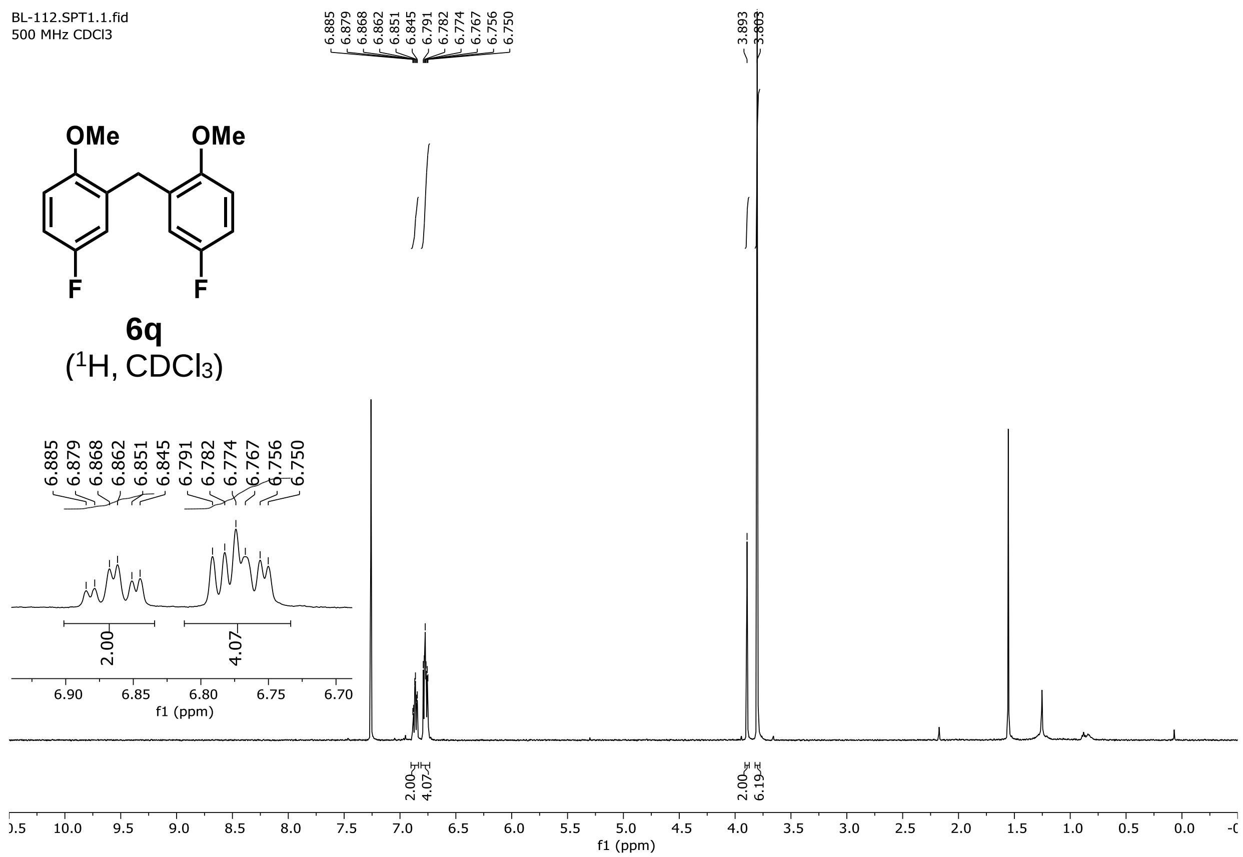
BL-112.SPT1.1.fid
500 MHz CDCl3
6.885 6.879 6.868 6.862 6.851 6.845 6.791 6.782 6.774 6.767 6.756 6.750
3.893 3.803
OMe
OMe
F
F
6q
(^{1}H, CDCl3)
2.00
4.07
6.90 6.85 6.80 6.75 6.70
f1 (ppm)
2.00
4.07
2.00
6.19
10.0 9.5 9.0 8.5 8.0 7.5 7.0 6.5 6.0 5.5 5.0 4.5 4.0 3.5 3.0 2.5 2.0 1.5 1.0 0.5 0.0
f1 (ppm)

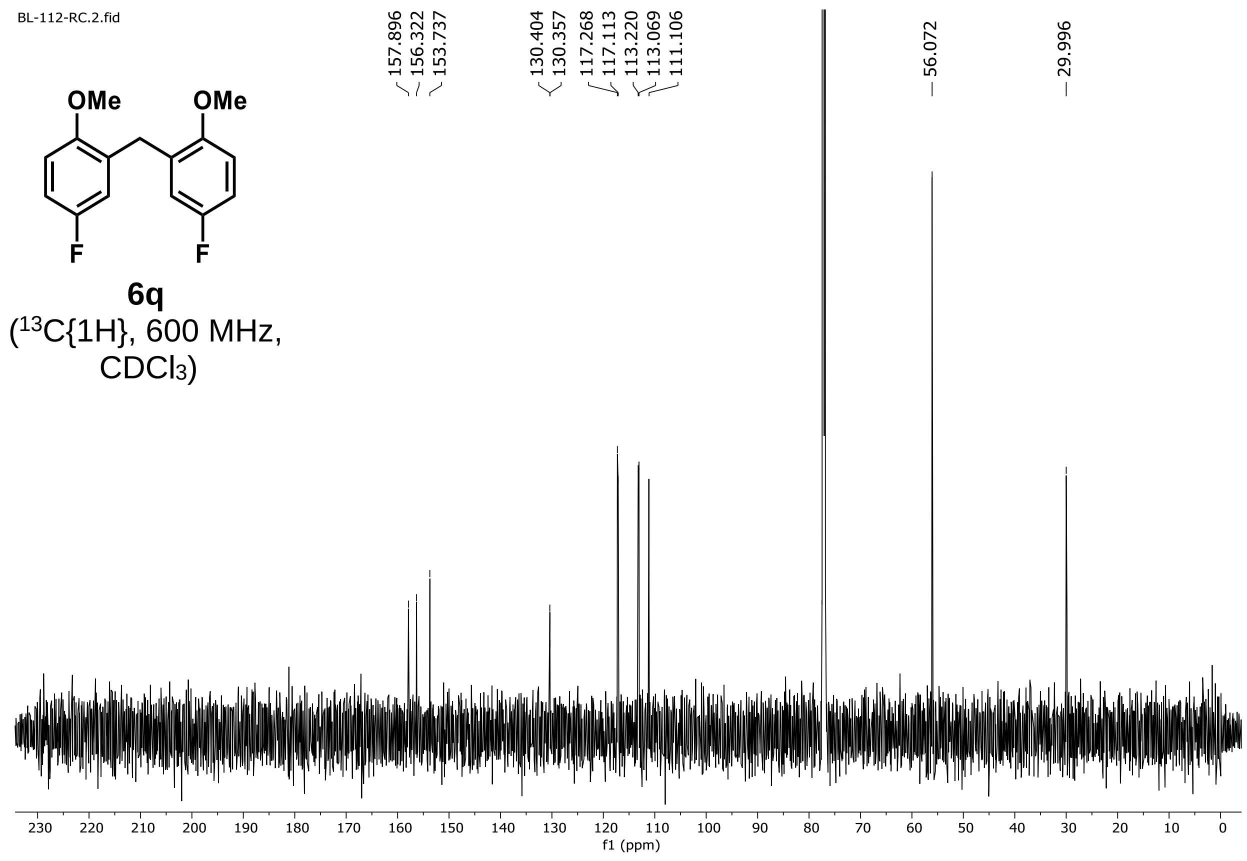
BL-112-RC.2.fid
157.896 156.322 153.737
130.404 130.357 117.268 117.113 113.220 113.069 111.106
56.072
29.996
OMe
OMe
F
F
6q
(^{13}C{1H}, 600 MHz,
CDCl3)
230 220 210 200 190 180 170 160 150 140 130 120 110 100 90 80 70 60 50 40 30 20 10 0
f1 (ppm)

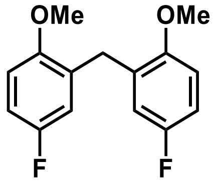

6q
(^{19}F, 600 MHz, $CDCl_3$)

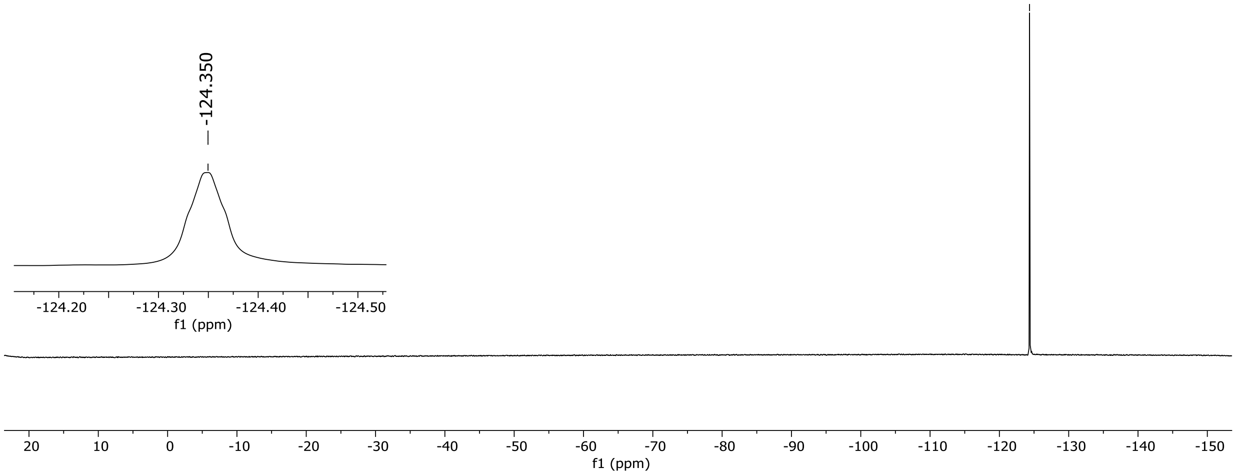

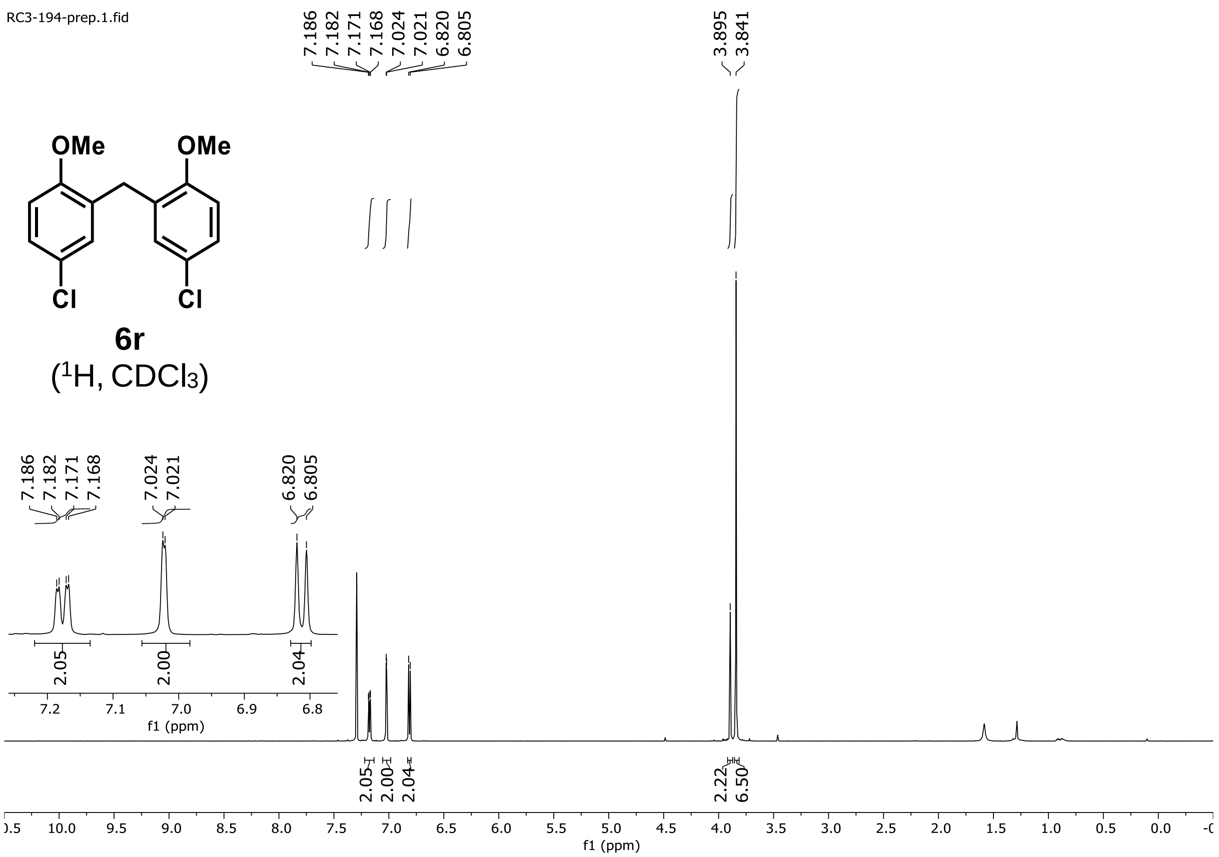

RC3-194-prep.1.fid
7.186
7.182
7.171
7.168
7.024
7.021
6.820
6.805
3.895
3.841
OMe
OMe
Cl
Cl
6r
(1H, CDCl3)
2.05
2.00
2.04
2.22
6.50
f1 (ppm)

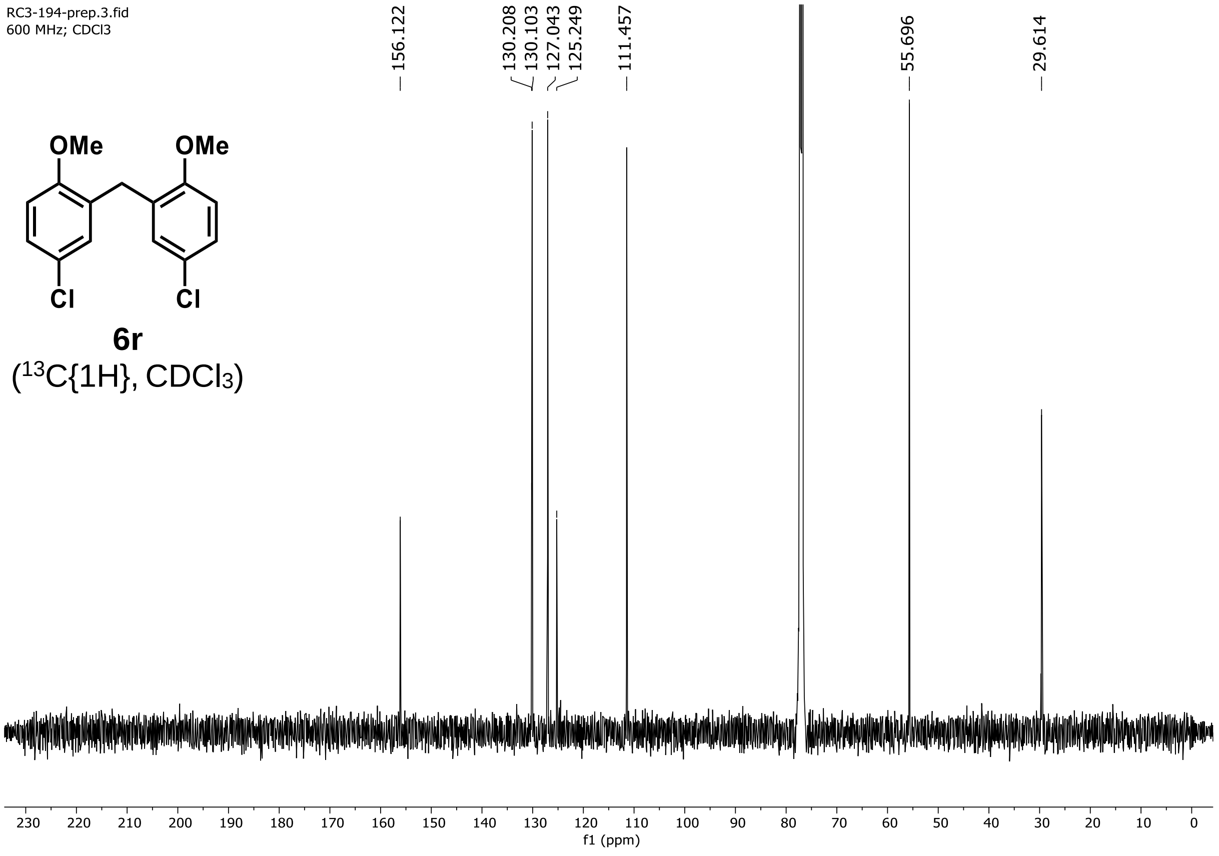

RC3-194-prep.3.fid
600 MHz; CDCl3
156.122
130.208
130.103
127.043
125.249
111.457
55.696
29.614
OMe
OMe
Cl
Cl
6r
(13C{1H}, CDCl3)
f1 (ppm)

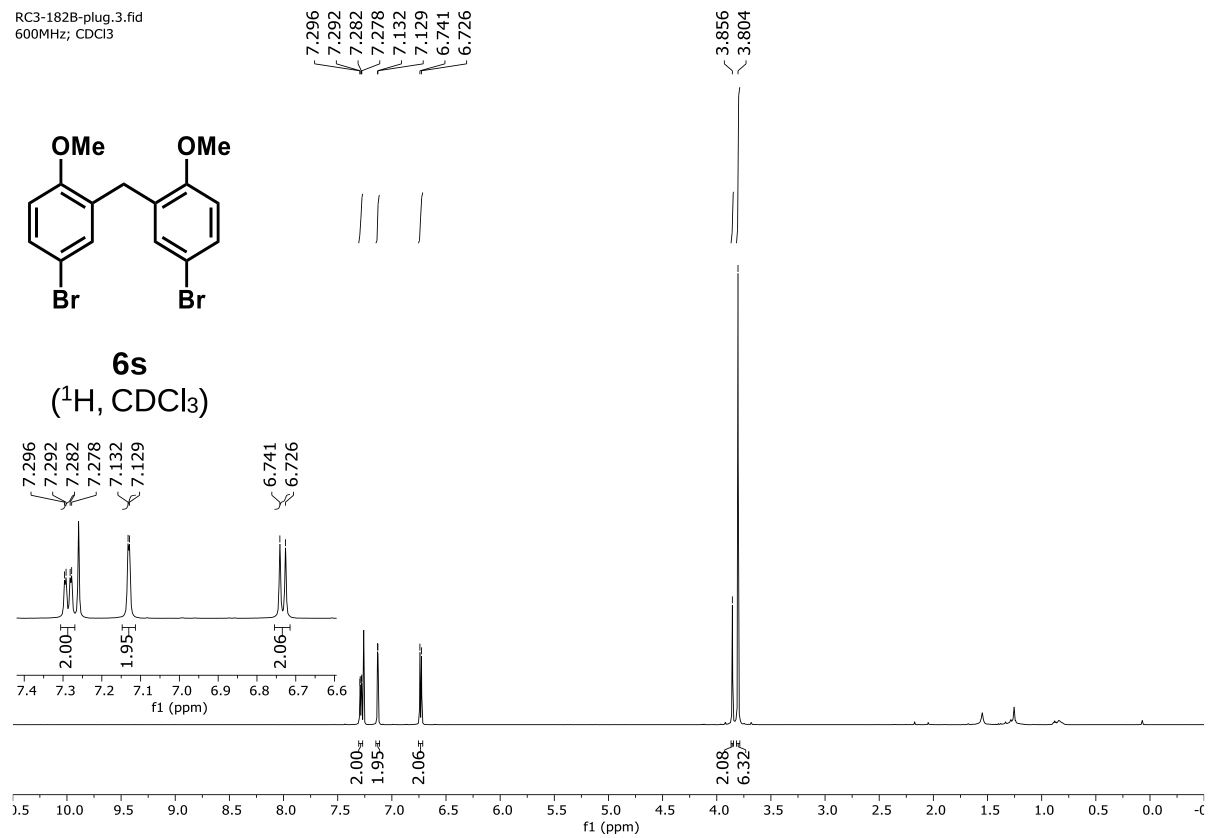
RC3-182B-plug.3.fid
600MHz; CDCl3
7.296
7.292
7.282
7.278
7.132
7.129
6.741
6.726
3.856
3.804
OMe
OMe
Br
Br
6s
(¹H, CDCl3)
2.00
1.95
2.06
2.08
6.32
f1 (ppm)

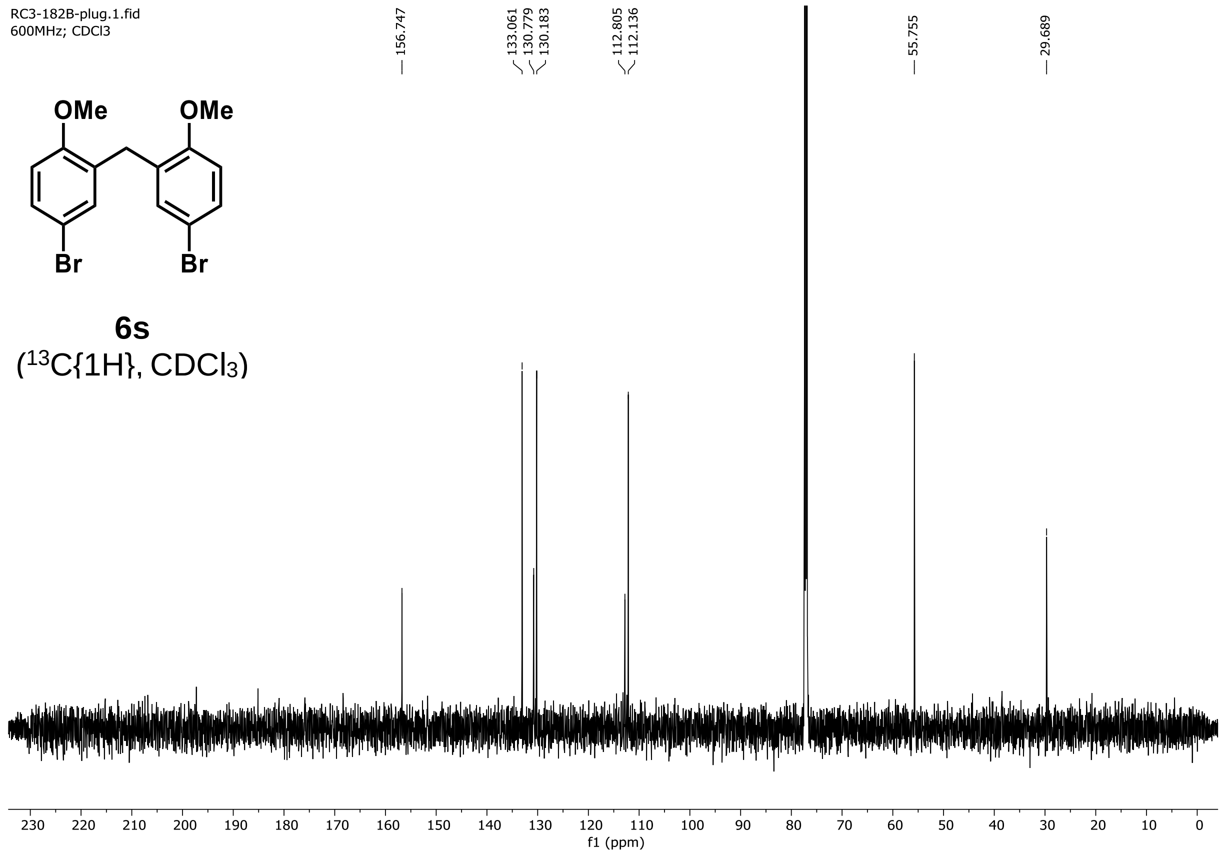
RC3-182B-plug.1.fid
600MHz; CDCl3
156.747
133.061
130.779
130.183
112.805
112.136
55.755
29.689
OMe
OMe
Br
Br
6s
(¹³C{1H}, CDCl3)
f1 (ppm)

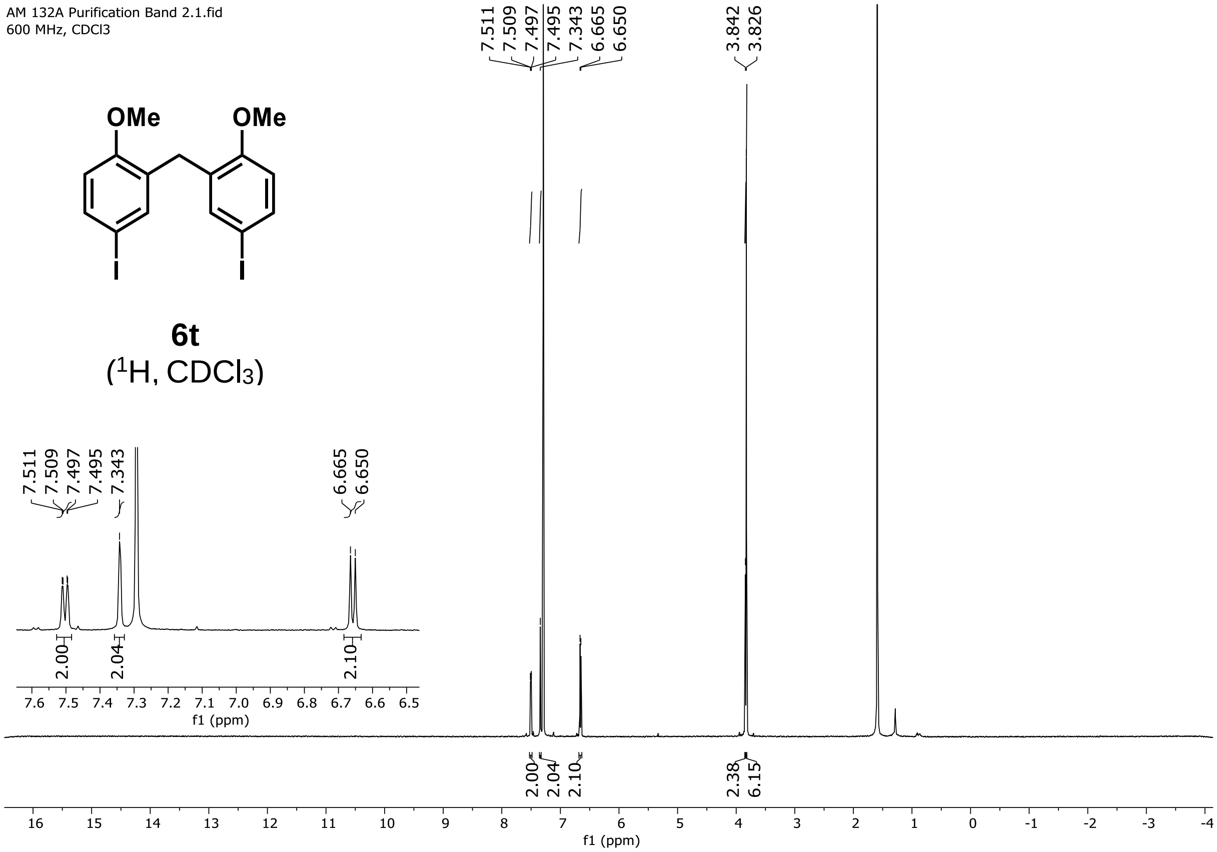
AM 132A Purification Band 2.1.fid
600 MHz, CDCl3
OMe
OMe
I
I
6t
(1H, CDCl3)
7.511
7.509
7.497
7.495
7.343
6.665
6.650
3.842
3.826
2.00
2.04
2.10
2.38
6.15
f1 (ppm)

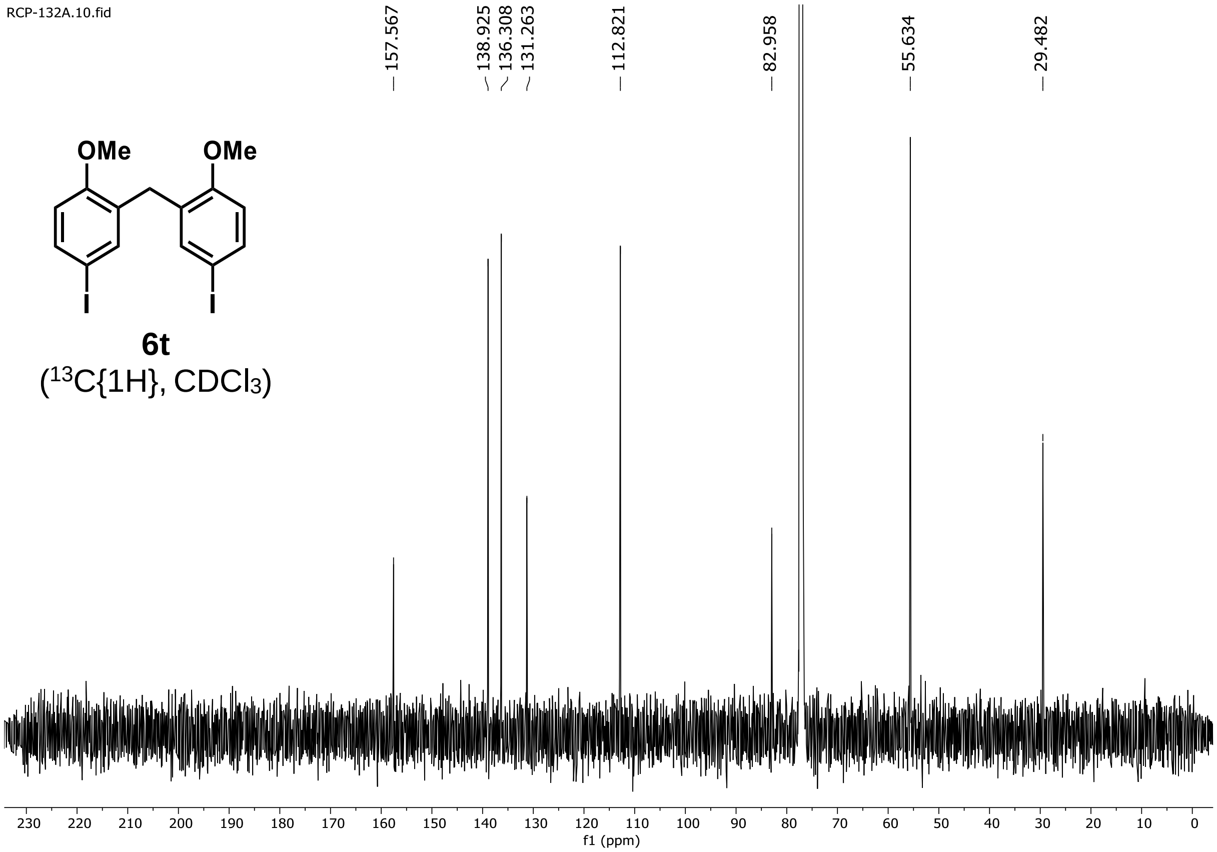
RCP-132A.10.fid
157.567
138.925
136.308
131.263
112.821
82.958
55.634
29.482
OMe
OMe
I
I
6t
(13C{1H}, CDCl3)
f1 (ppm)

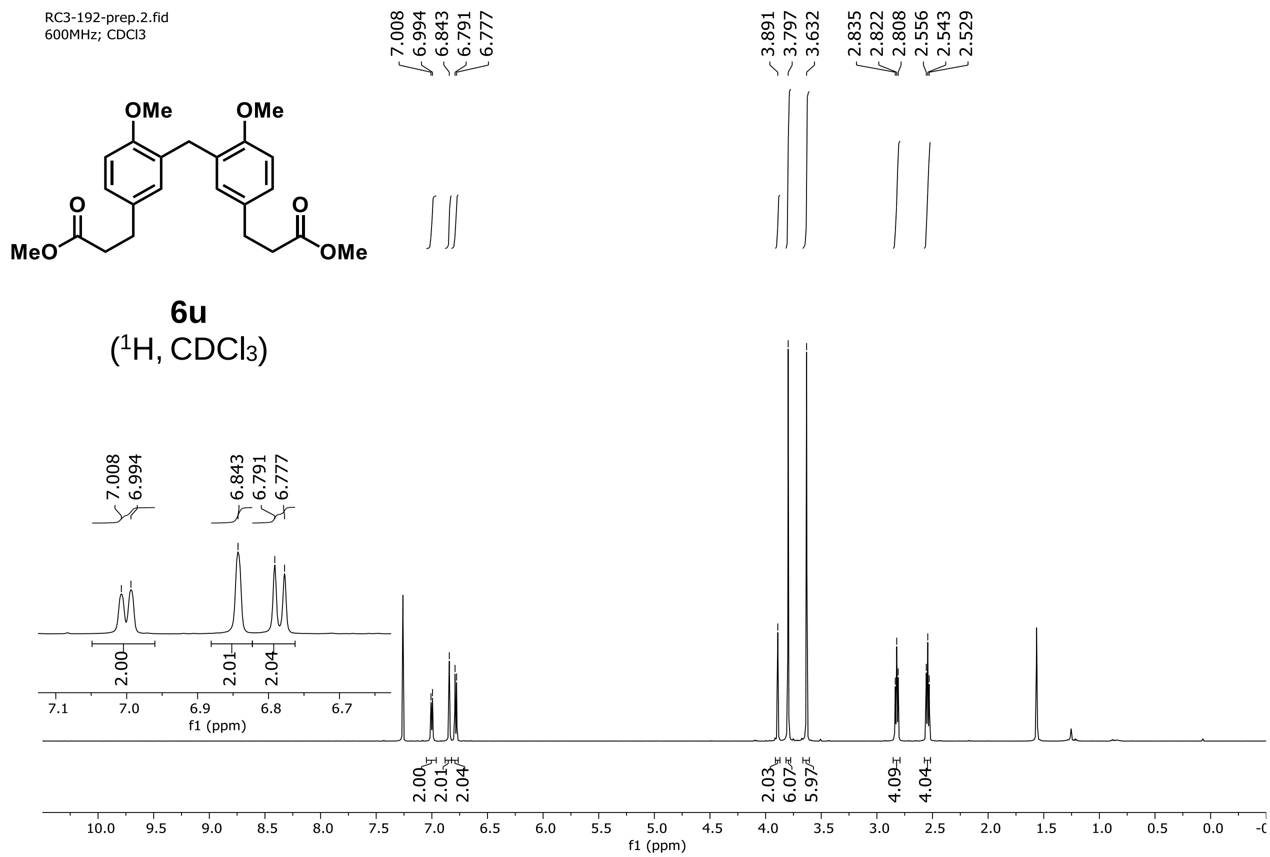
RC3-192-prep.2.fid
600MHz; CDCl3
OMe
OMe
O
O
MeO
OMe
6u
(^{1}H, CDCl$_3$)
7.008
6.994
6.843
6.791
6.777
3.891
3.797
3.632
2.835
2.822
2.808
2.556
2.543
2.529
2.00
2.01
2.04
2.03
6.07
5.97
4.09
4.04
f1 (ppm)

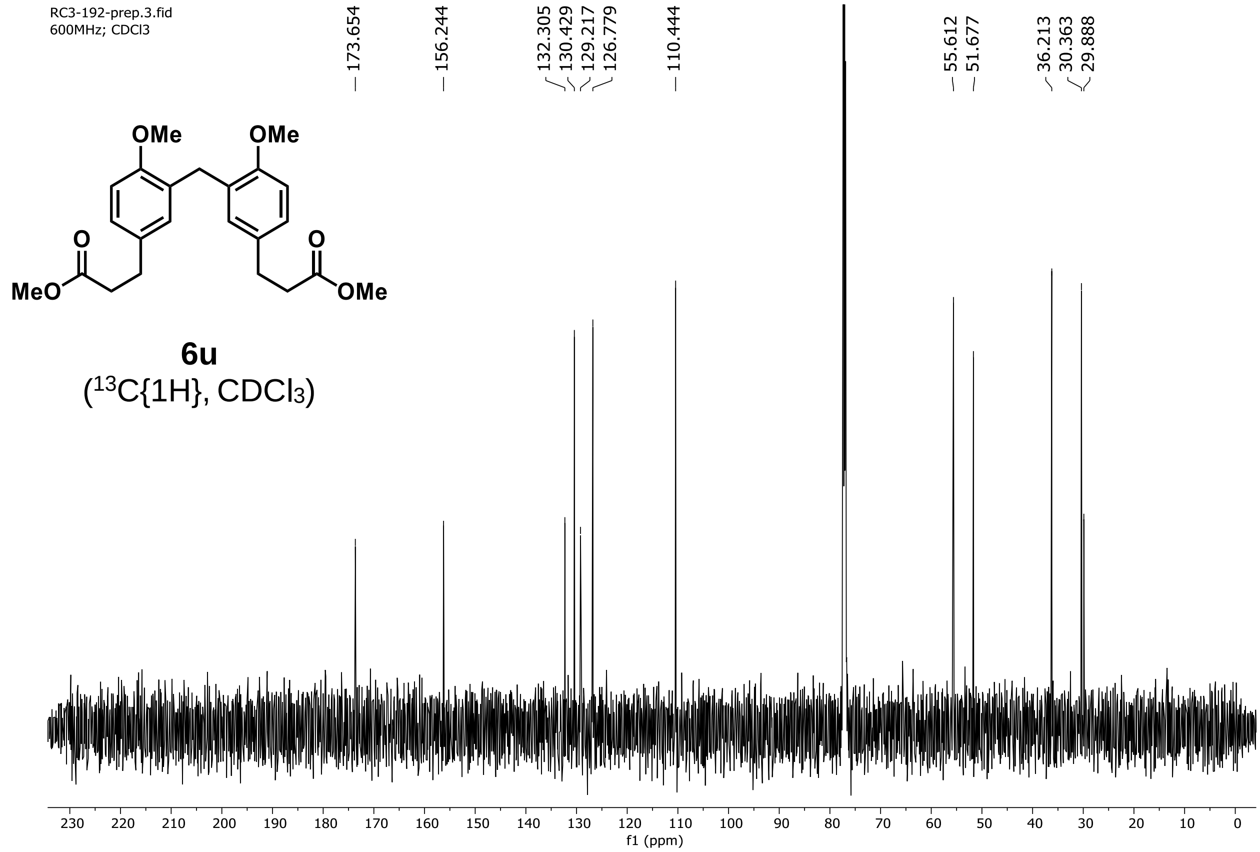
RC3-192-prep.3.fid
600MHz; CDCl3
173.654
156.244
132.305
130.429
129.217
126.779
110.444
55.612
51.677
36.213
30.363
29.888
OMe
OMe
O
O
MeO
OMe
6u
(^{13}C{1H}, CDCl$_3$)
f1 (ppm)

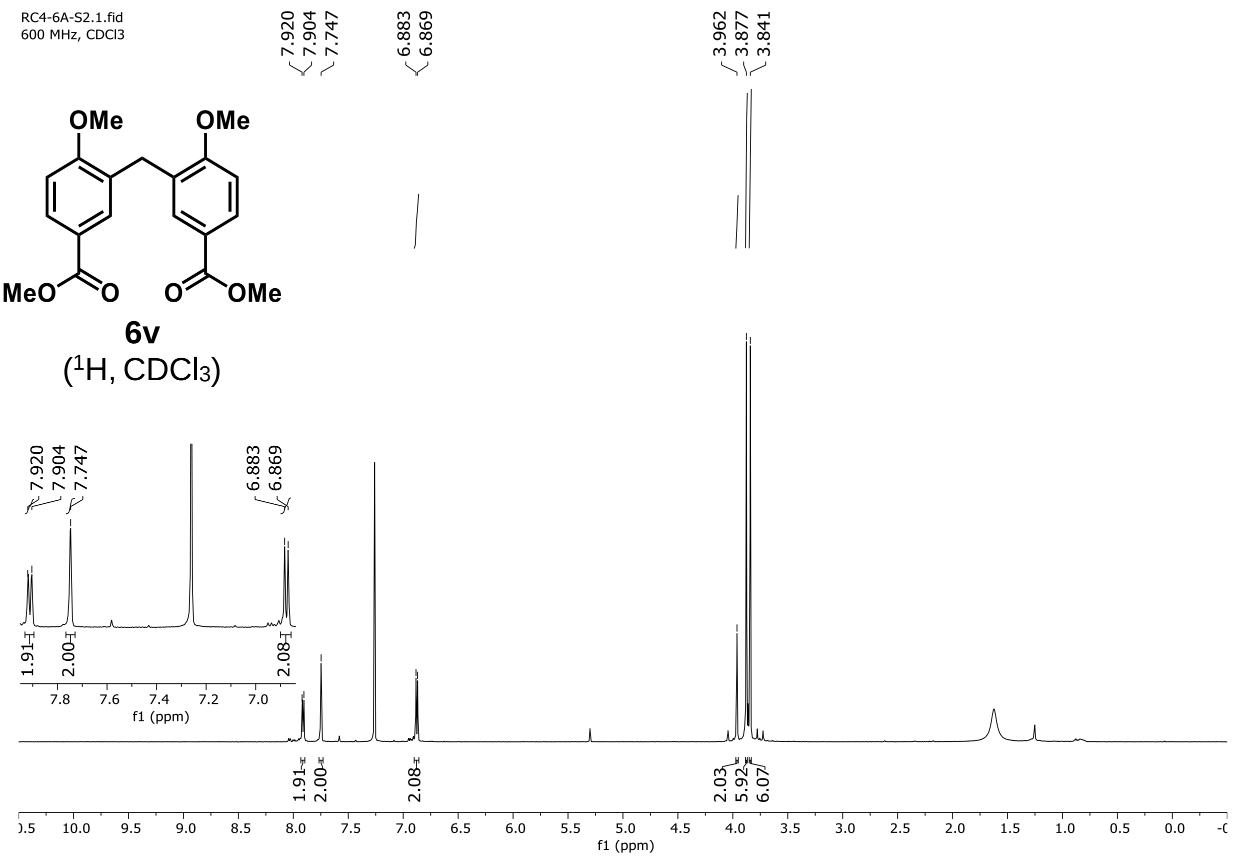
RC4-6A-S2.1.fid
600 MHz, CDCl3
7.920
7.904
7.747
6.883
6.869
3.962
3.877
3.841
OMe
OMe
MeO
O
O
OMe
6v
(1H, CDCl3)
1.91
2.00
2.08
7.8 7.6 7.4 7.2 7.0
f1 (ppm)
2.03
5.92
6.07
10.0 9.5 9.0 8.5 8.0 7.5 7.0 6.5 6.0 5.5 5.0 4.5 4.0 3.5 3.0 2.5 2.0 1.5 1.0 0.5 0.0
f1 (ppm)

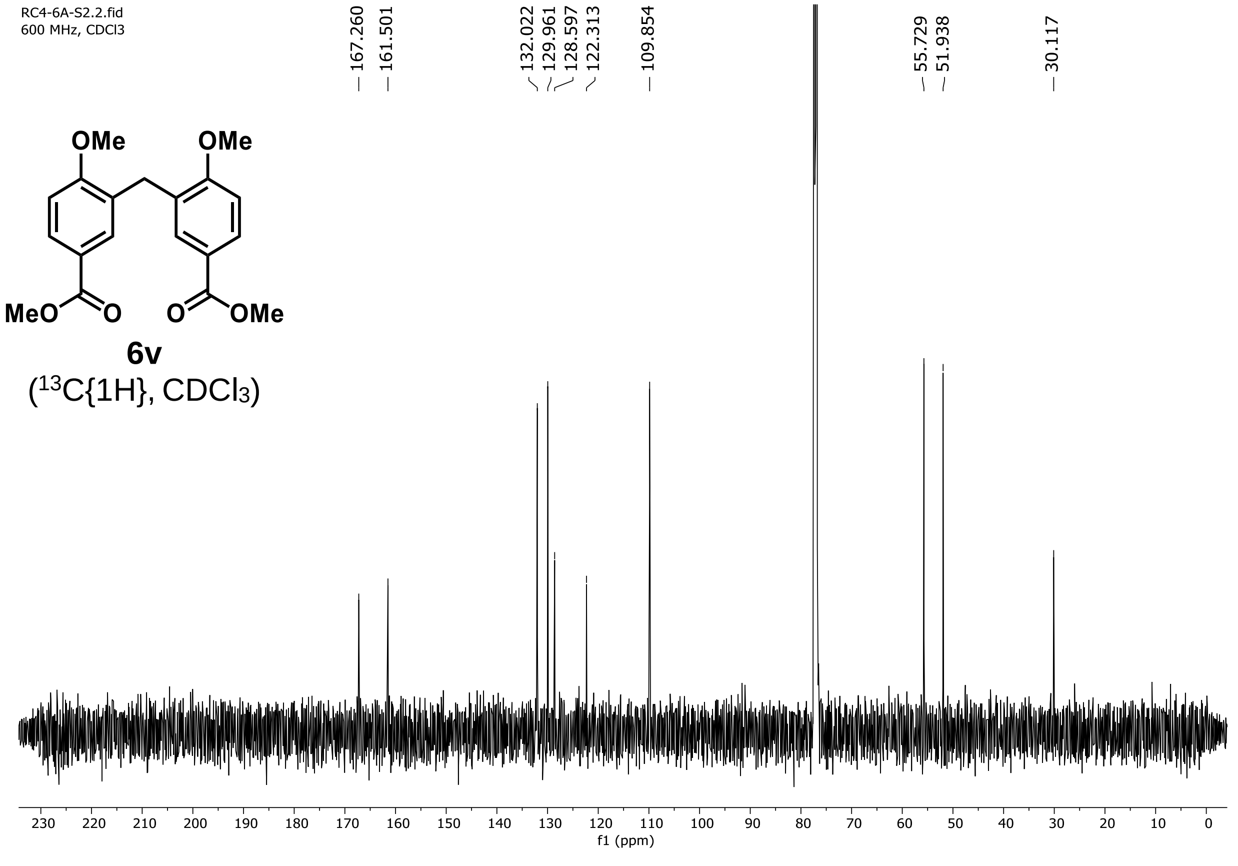
RC4-6A-S2.2.fid
600 MHz, CDCl3
167.260
161.501
132.022
129.961
128.597
122.313
109.854
55.729
51.938
30.117
OMe
OMe
MeO
O
O
OMe
6v
(13C{1H}, CDCl3)
230 220 210 200 190 180 170 160 150 140 130 120 110 100 90 80 70 60 50 40 30 20 10 0
f1 (ppm)

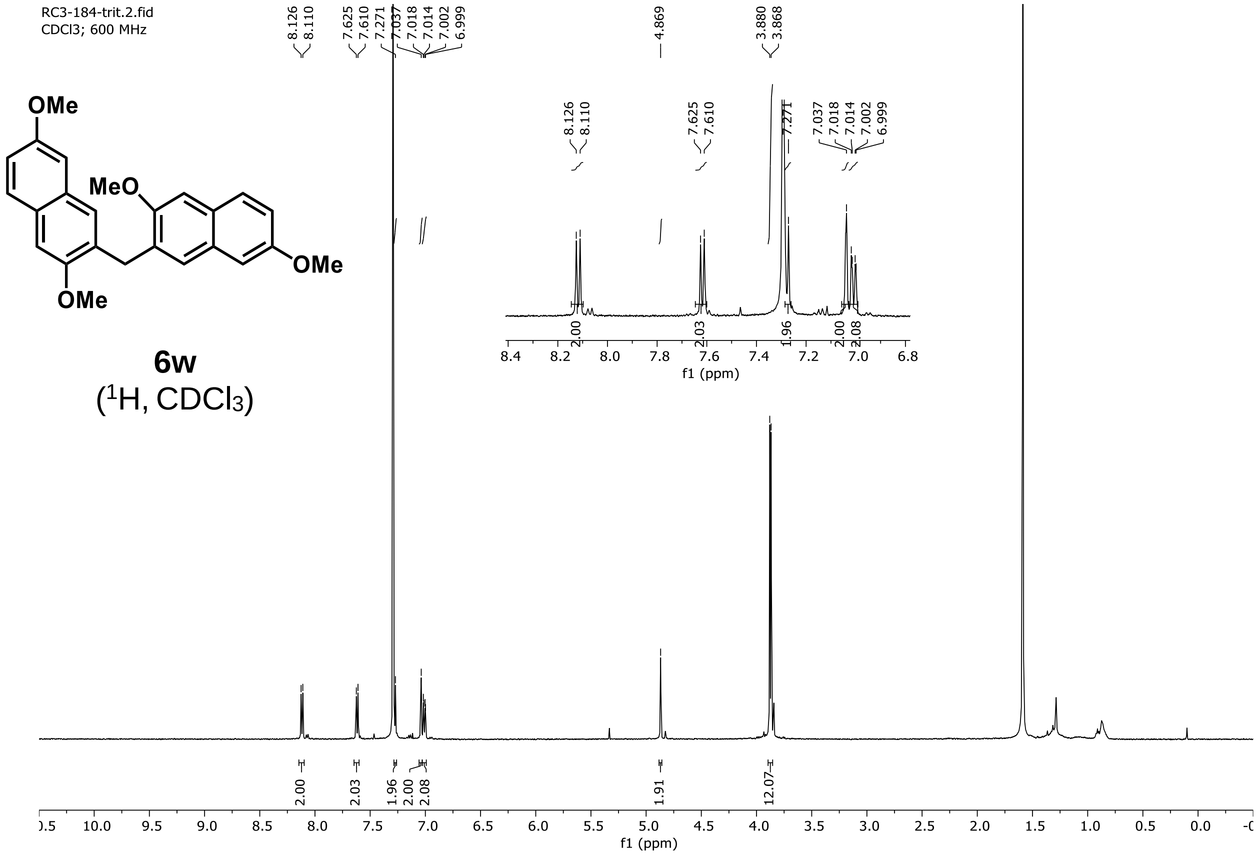

6w
(^{1}H, $CDCl_3$)

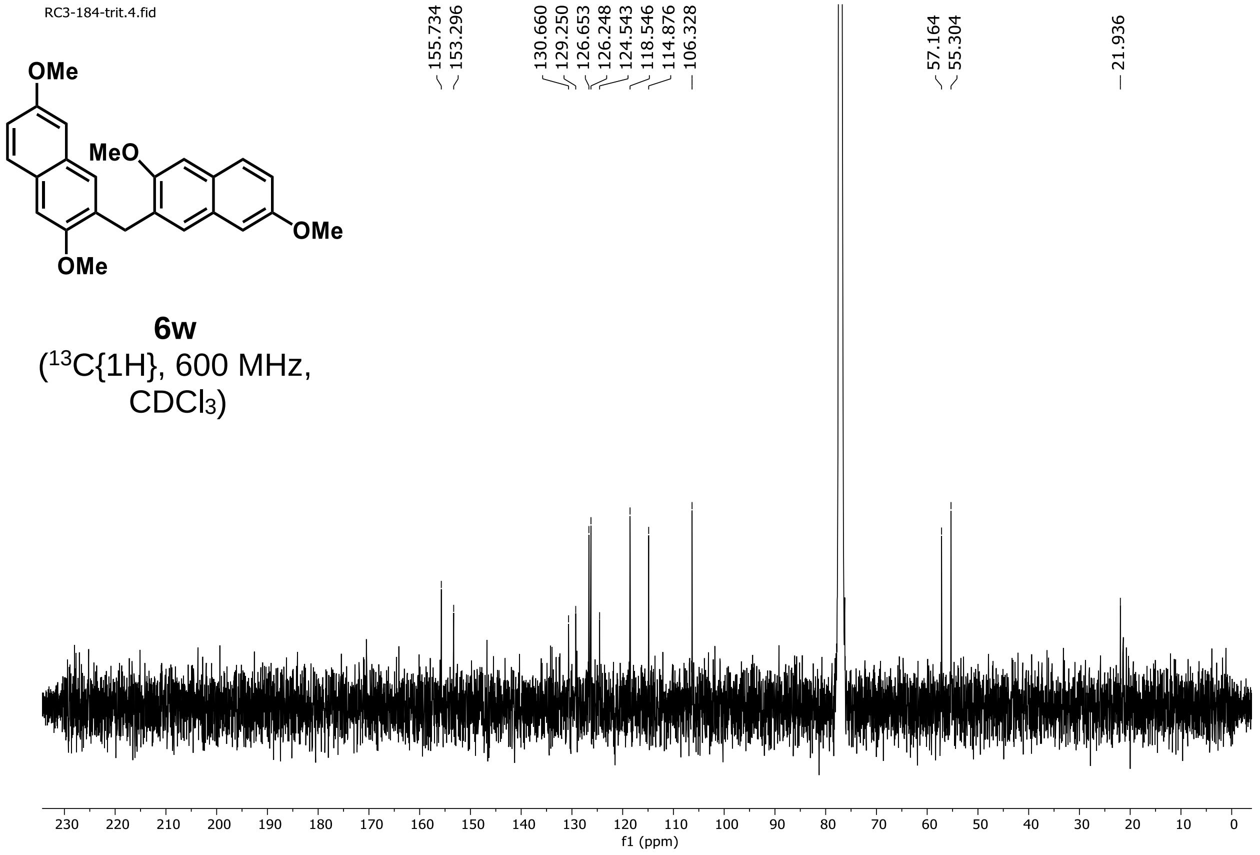

6w
(^{13}C{1H}, 600 MHz, $CDCl_3$)

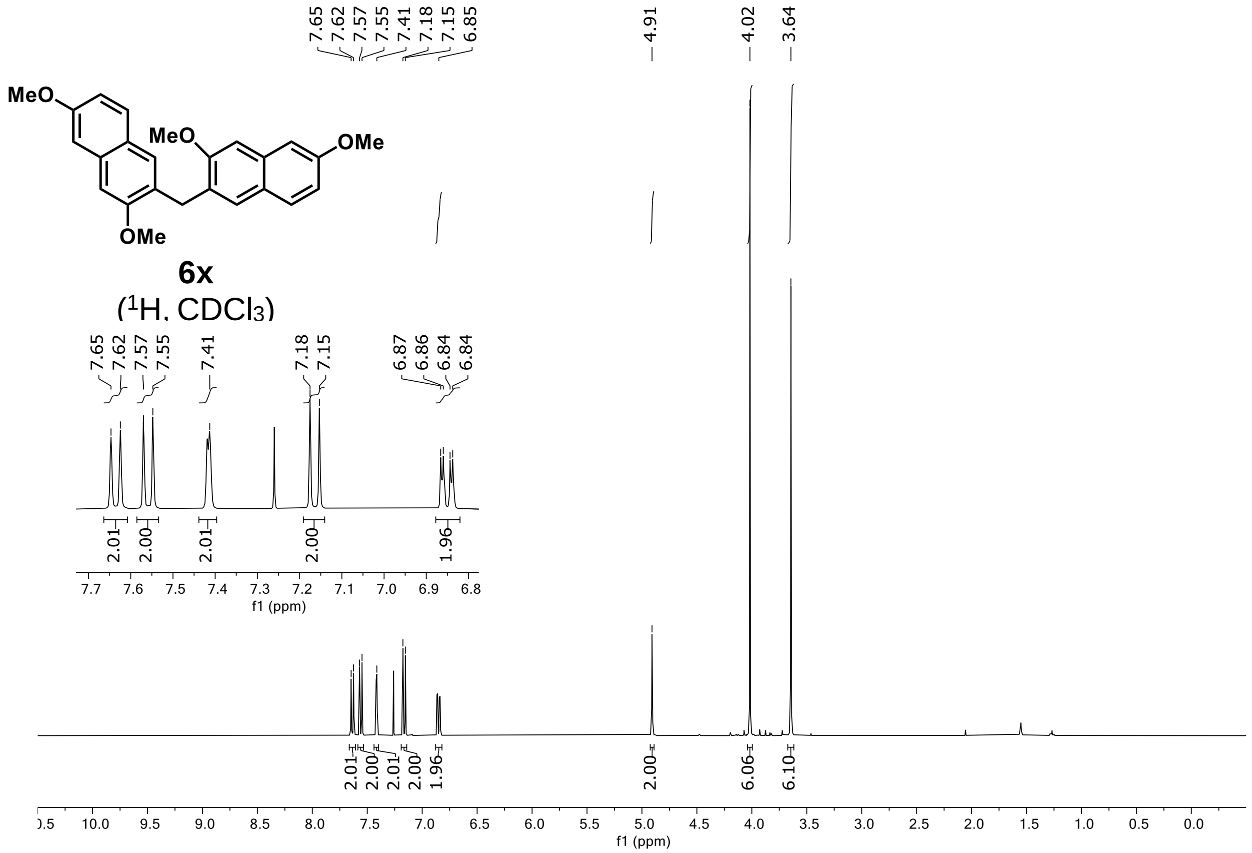
7.65
7.62
7.57
7.55
7.41
7.18
7.15
6.85
4.91
4.02
3.64
MeO
MeO
OMe
OMe
6x
(1H, CDCl3)
6.87
6.86
6.84
6.84
2.01
2.00
2.01
2.00
1.96
6.06
6.10
f1 (ppm)

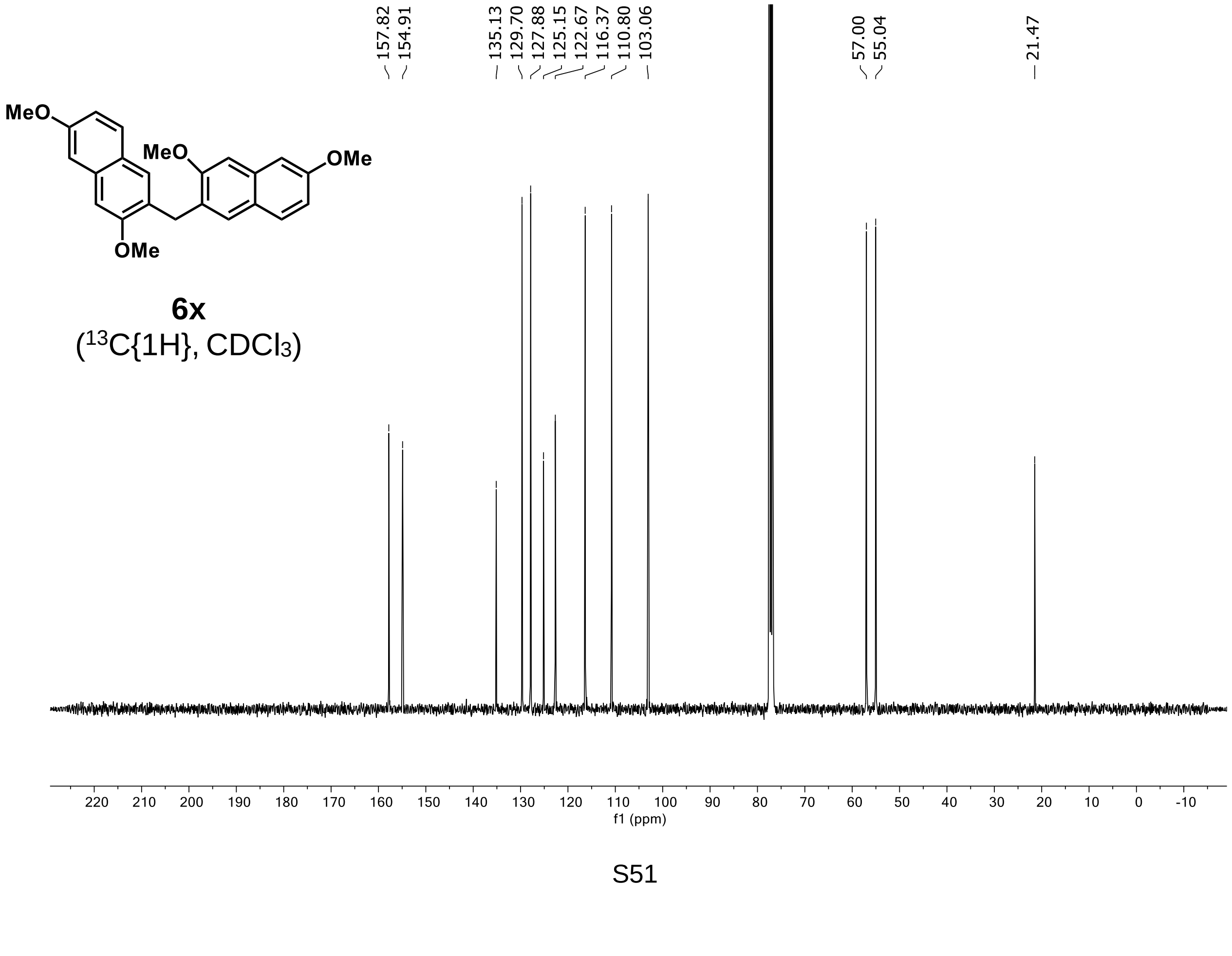
157.82
154.91
135.13
129.70
127.88
125.15
122.67
116.37
110.80
103.06
57.00
55.04
21.47
MeO
MeO
OMe
OMe
6x
(13C{1H}, CDCl3)
f1 (ppm)

S-7x
(^{1}H, $CDCl_3$)

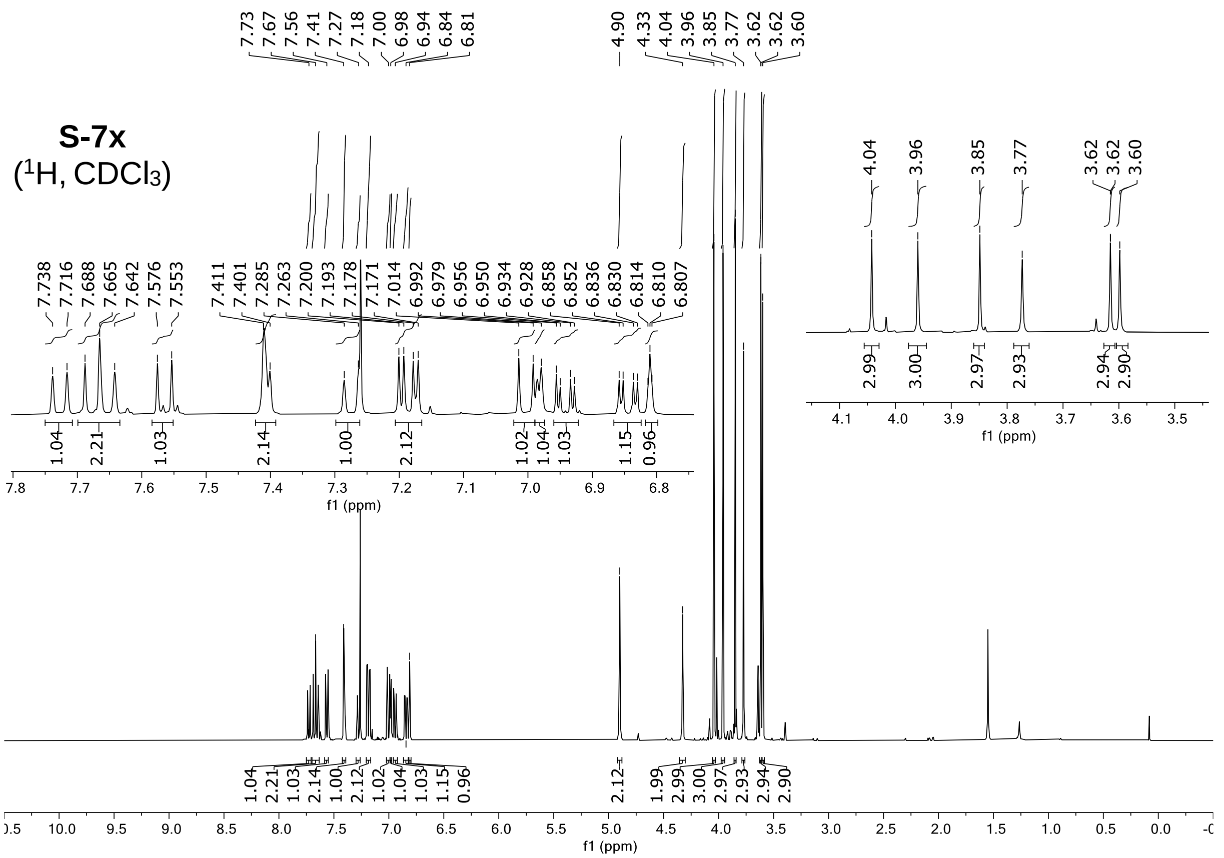

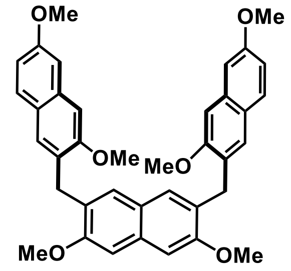

S-7x
($^{13}C\{1H\}$, $CDCl_3$)

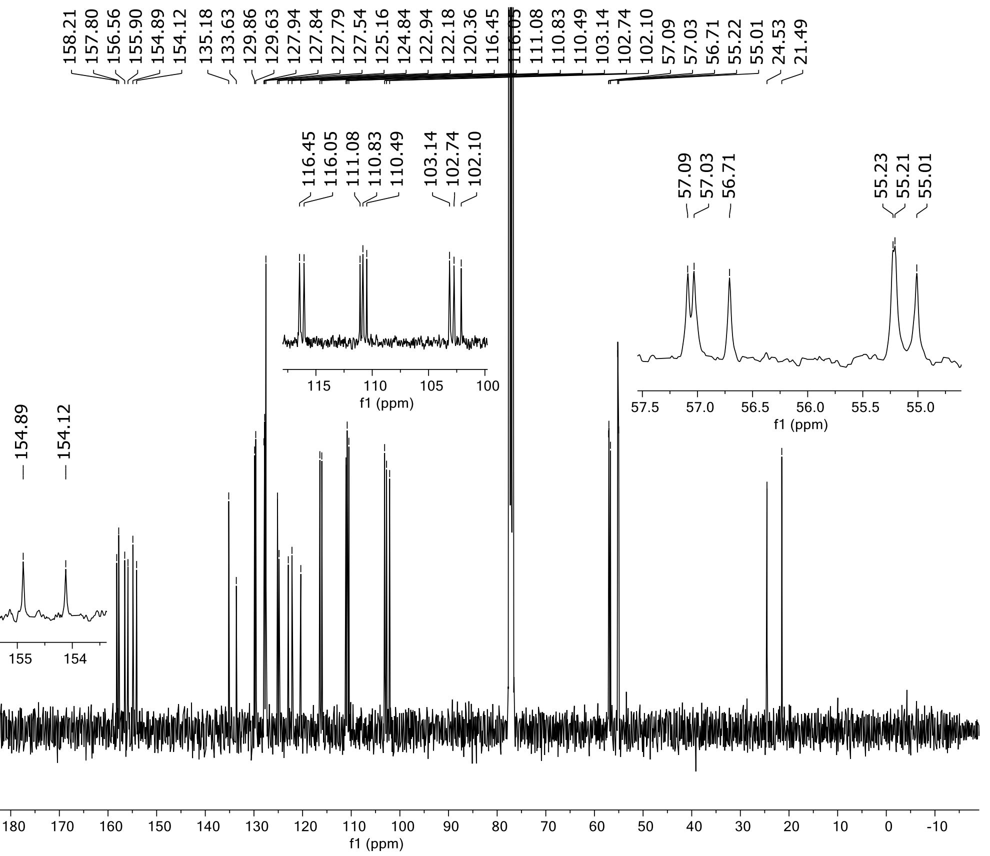

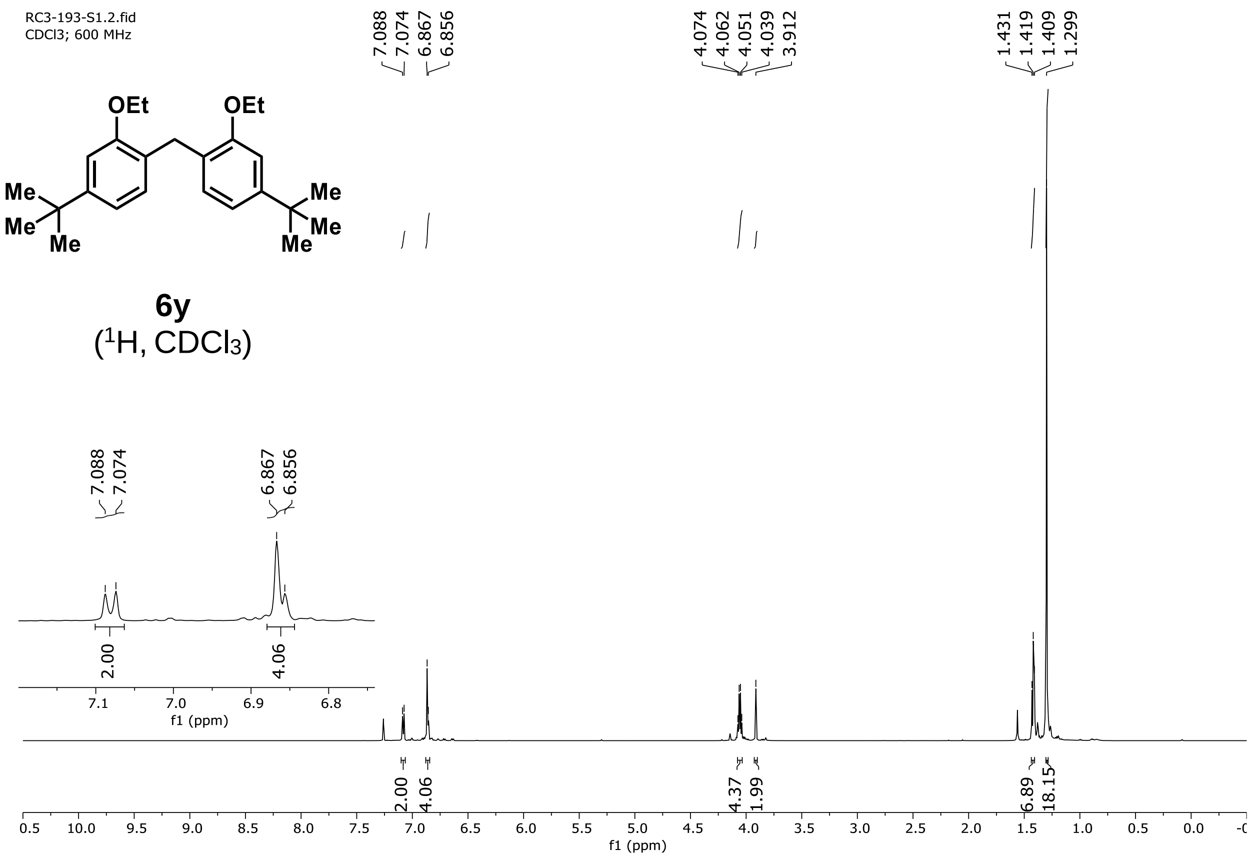

RC3-193-S1.2.fid
CDCl3; 600 MHz
OEt
OEt
Me
Me
Me
Me
Me
Me
6y
(1H, CDCl3)
7.088
7.074
6.867
6.856
4.074
4.062
4.051
4.039
3.912
1.431
1.419
1.409
1.299
2.00
4.06
4.37
1.99
6.89
18.15
f1 (ppm)

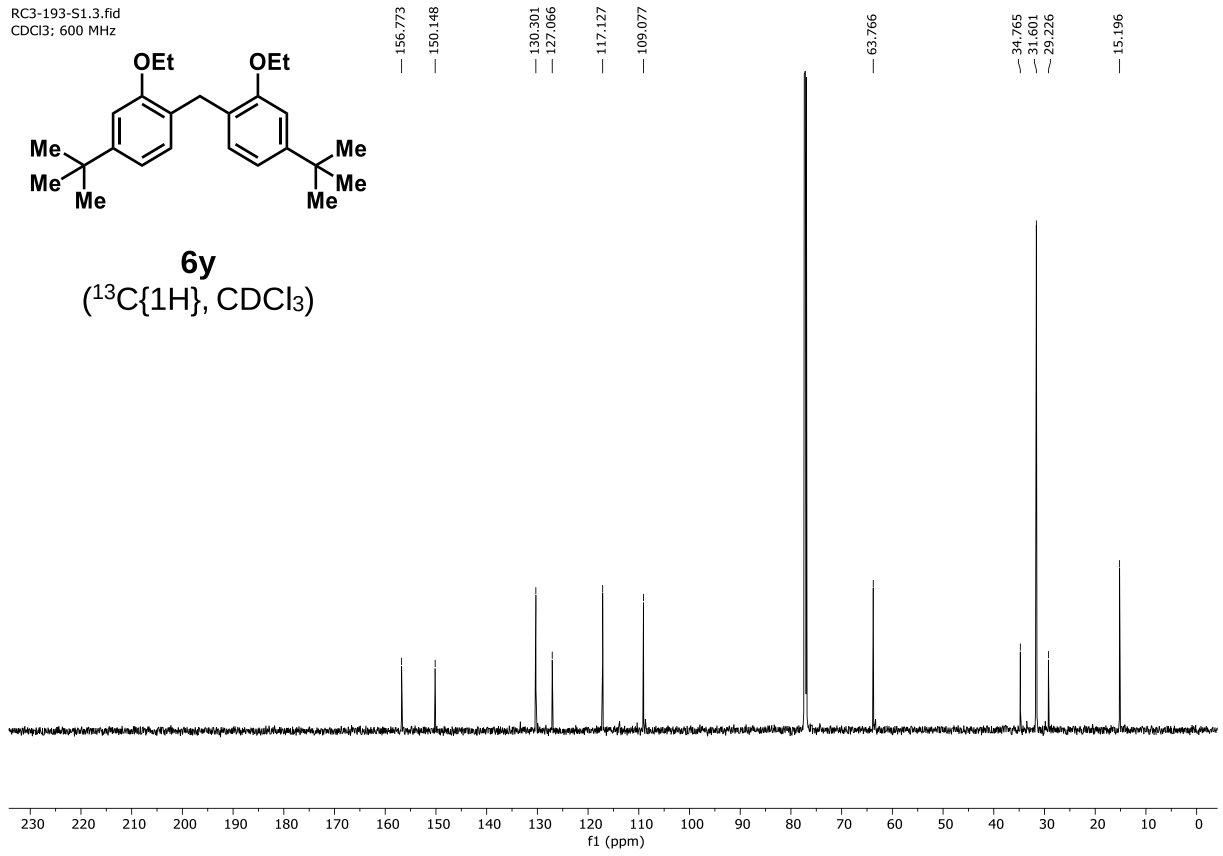

RC3-193-S1.3.fid
CDCl3; 600 MHz
OEt
OEt
Me
Me
Me
Me
Me
Me
6y
(13C{1H}, CDCl3)
156.773
150.148
130.301
127.066
117.127
109.077
63.766
34.765
31.601
29.226
15.196
f1 (ppm)

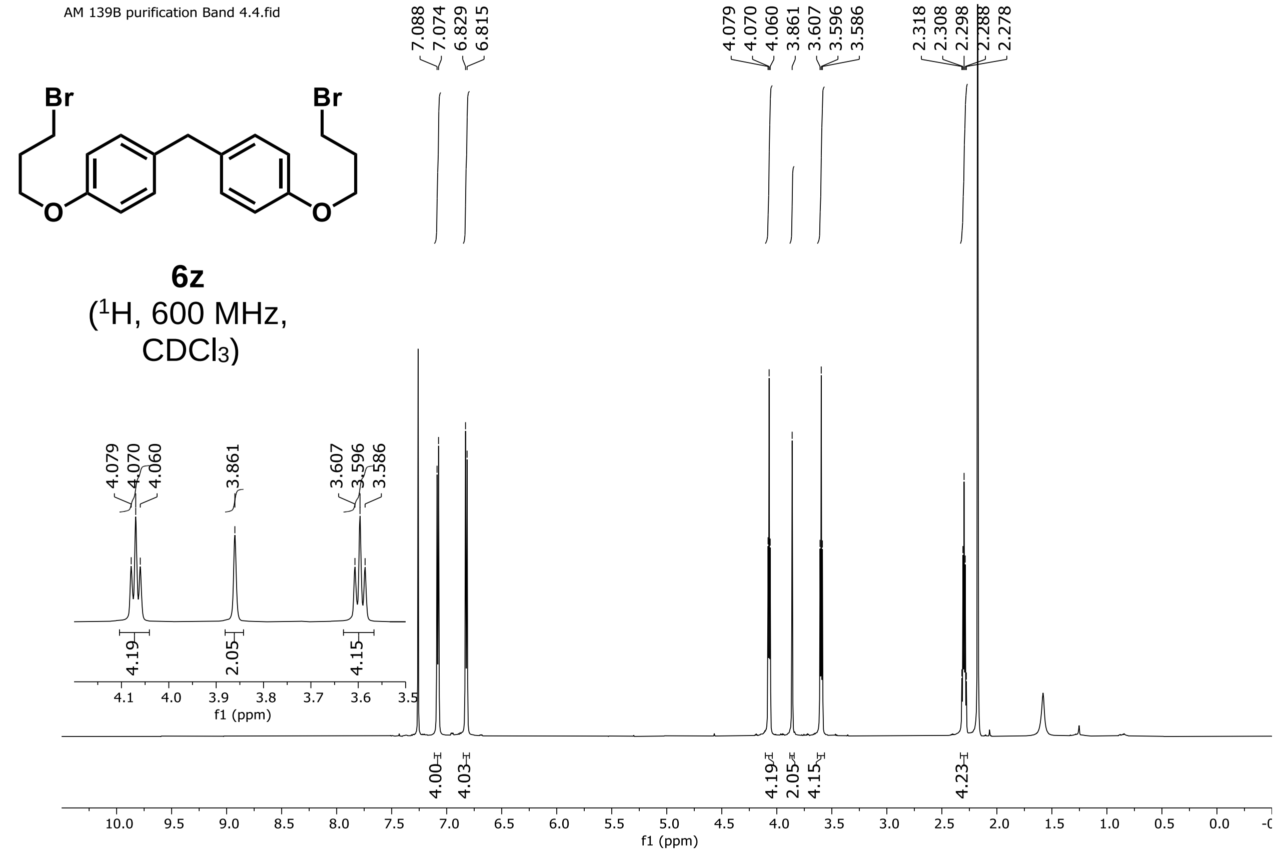

6z
(^{1}H, 600 MHz, $CDCl_3$)

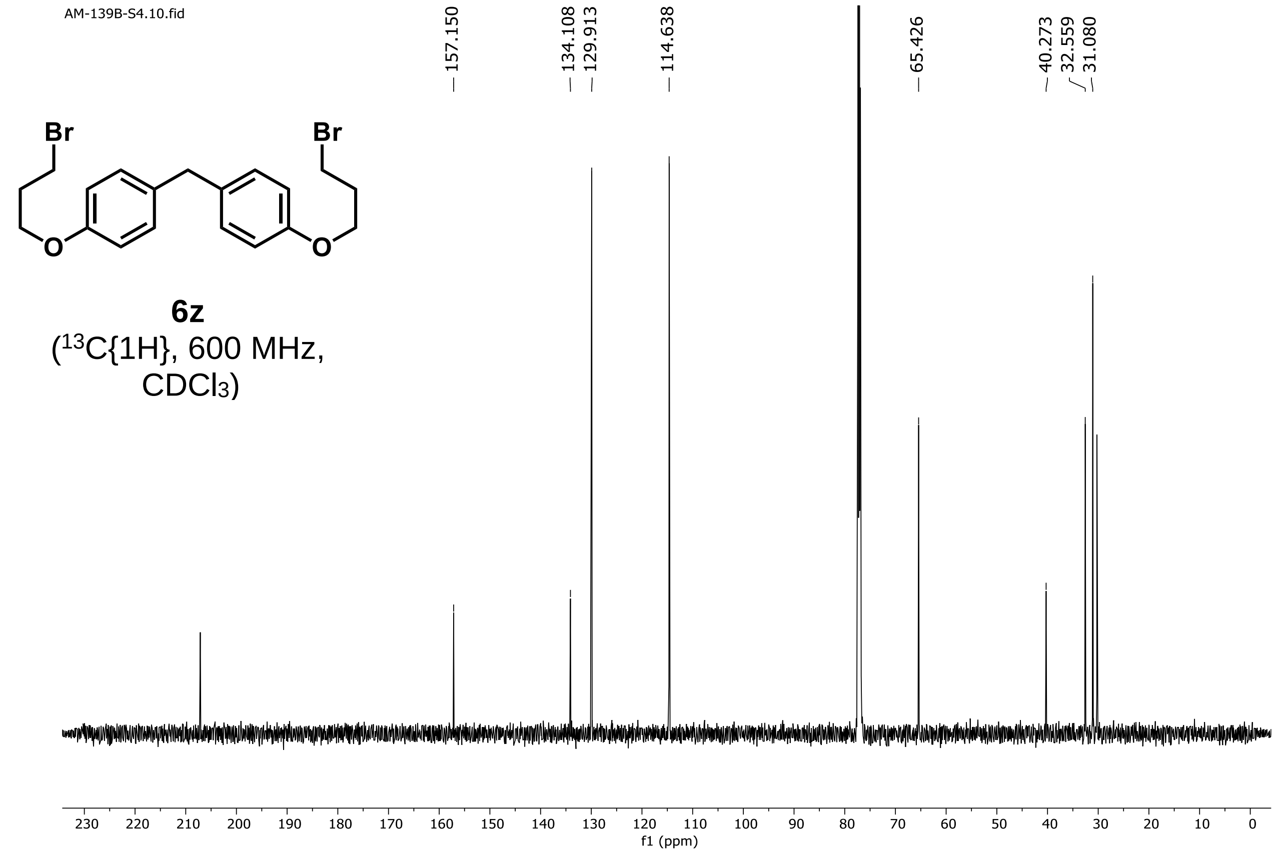

6z
(^{13}C{1H}, 600 MHz, $CDCl_3$)

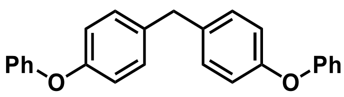

6za
(^{1}H, $CDCl_3$)

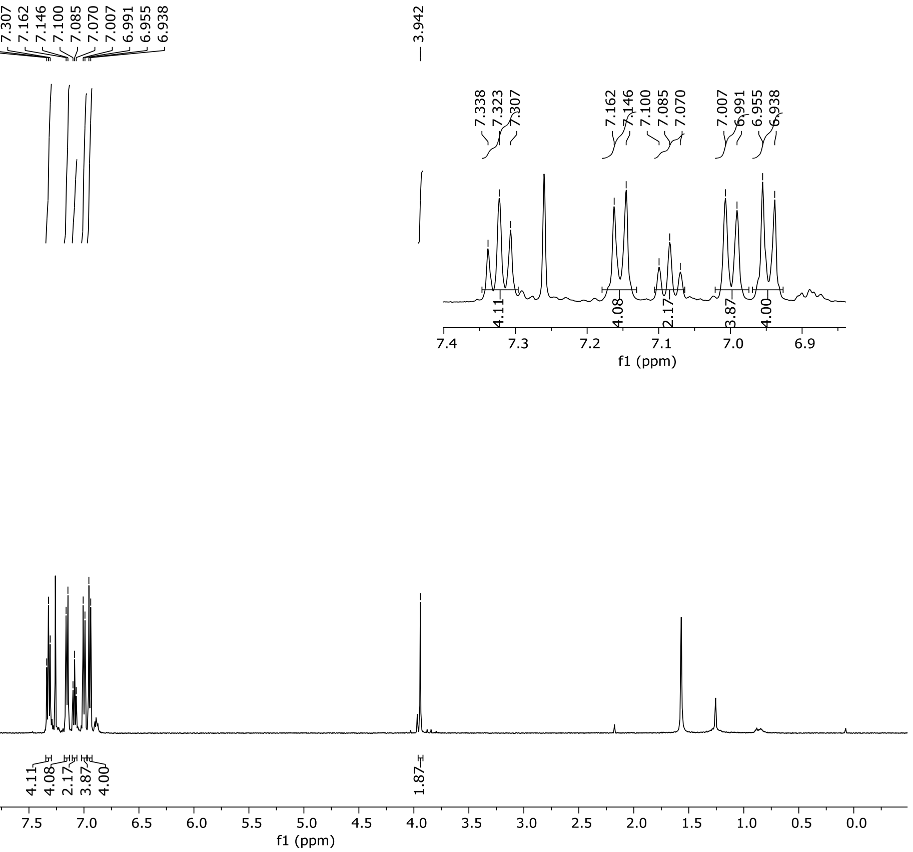

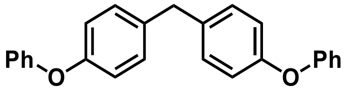

6za
(^{13}C{1H}, $CDCl_3$)

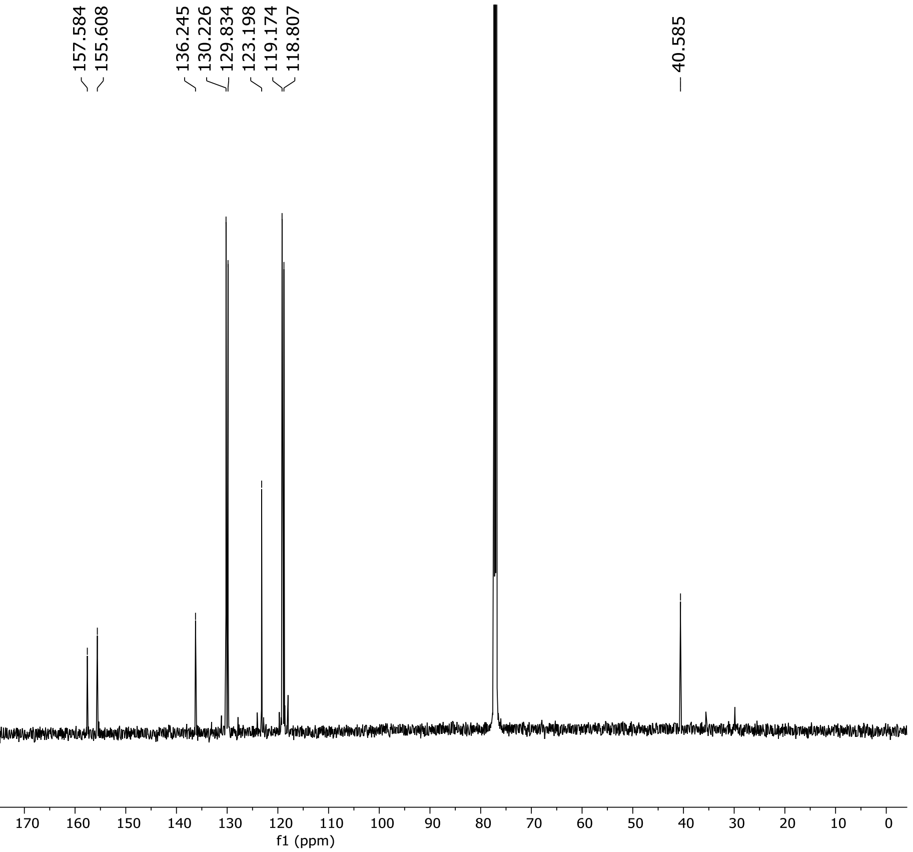

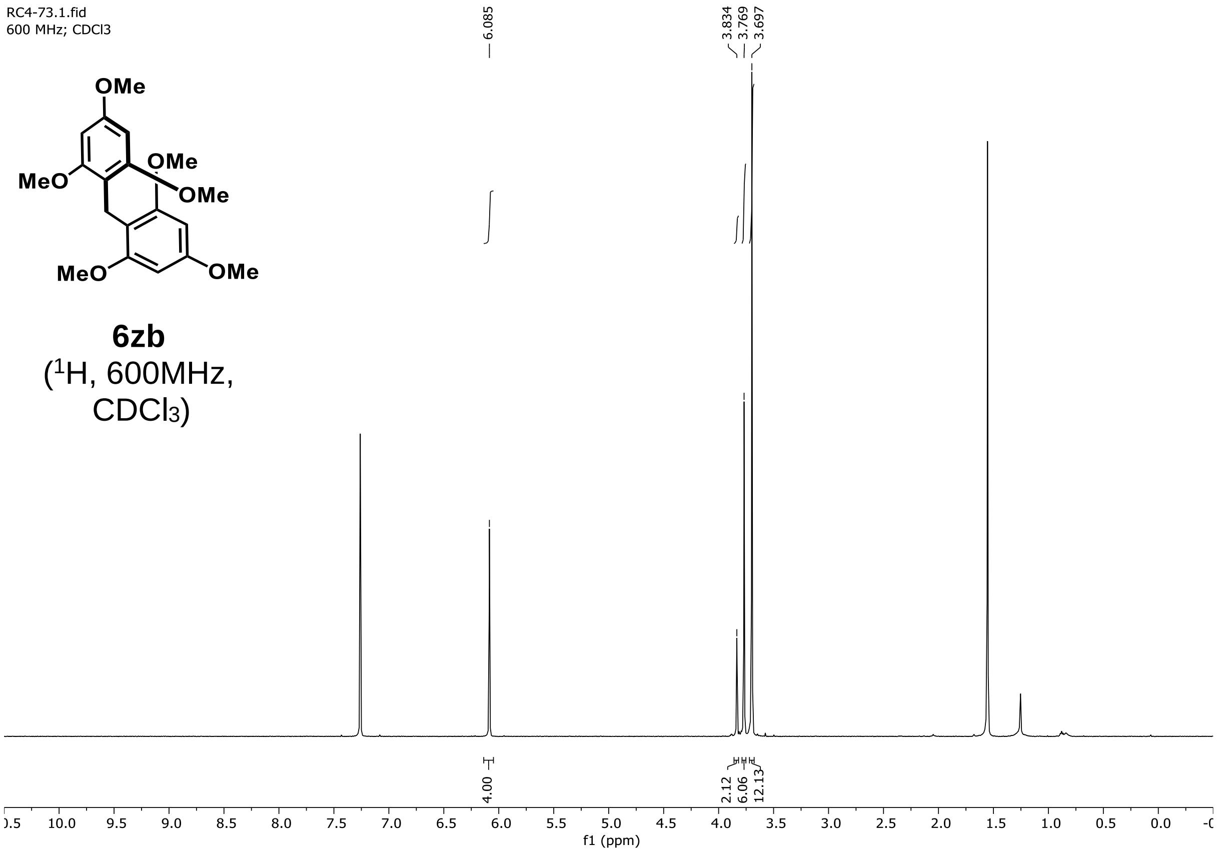
RC4-73.1.fid
600 MHz; CDCl3
OMe
MeO
OMe
OMe
MeO
OMe
6zb
(1H, 600MHz, CDCl3)
6.085
3.834
3.769
3.697
4.00
2.12
6.06
12.13
f1 (ppm)

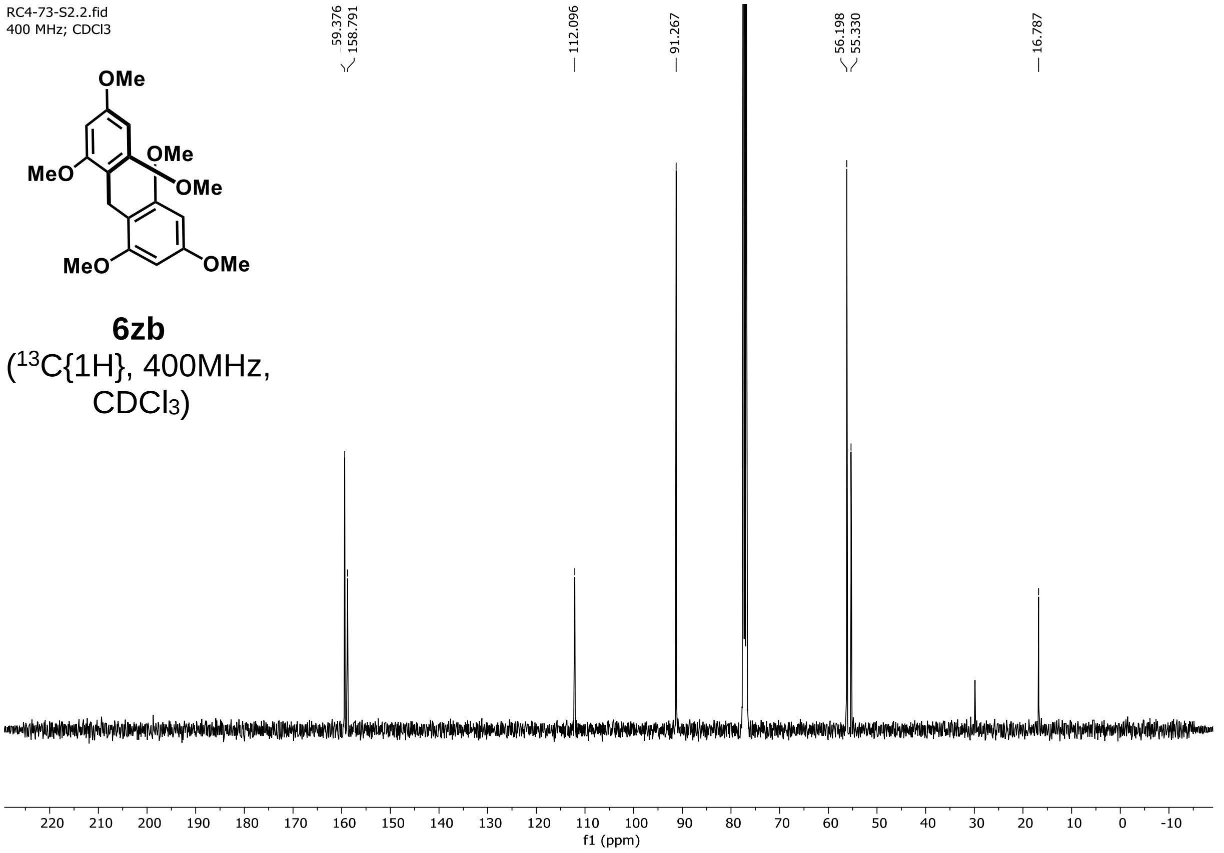
RC4-73-S2.2.fid
400 MHz; CDCl3
OMe
MeO
OMe
OMe
MeO
OMe
6zb
(13C{1H}, 400MHz, CDCl3)
159.376
158.791
112.096
91.267
56.198
55.330
16.787
f1 (ppm)

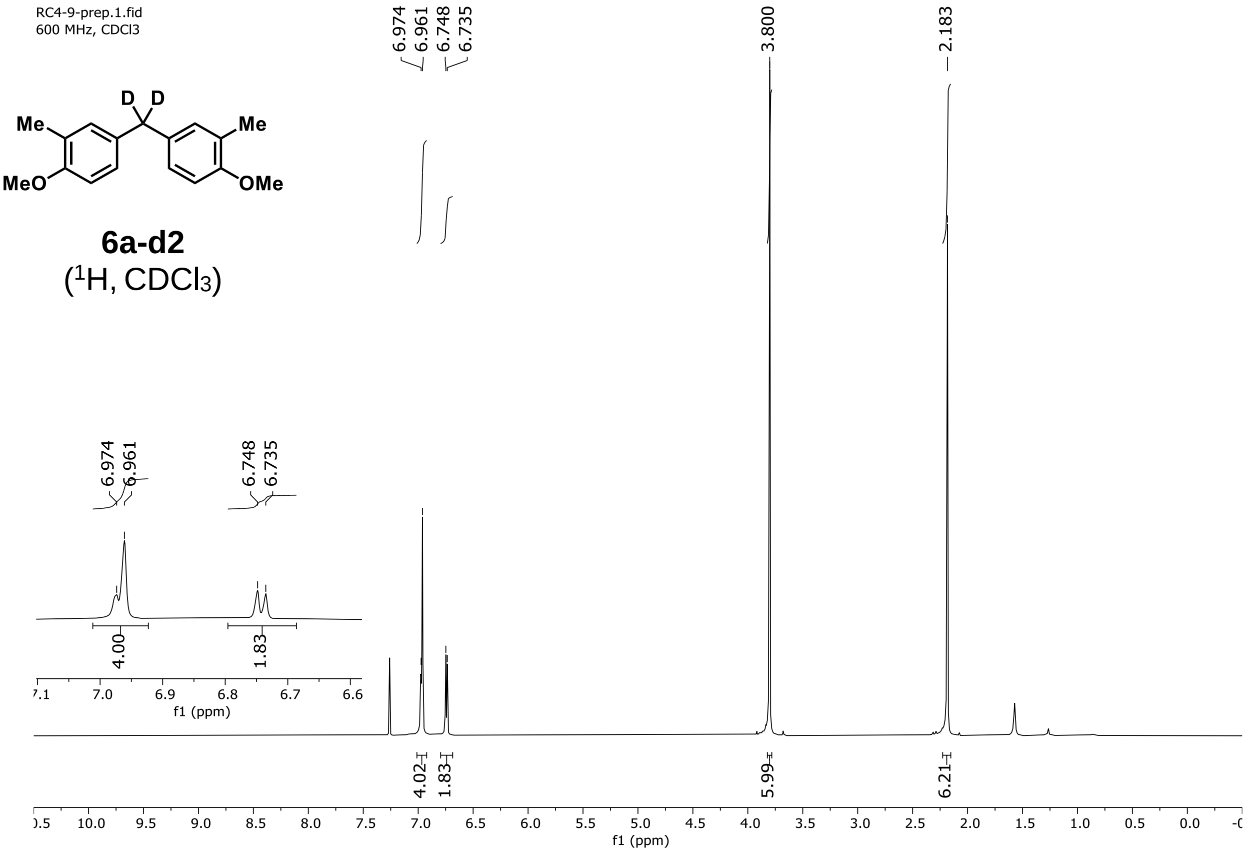
RC4-9-prep.1.fid
600 MHz, CDCl3
6a-d2
(1H, CDCl3)
6.974
6.961
6.748
6.735
3.800
2.183
4.00
1.83
4.02
1.83
5.99
6.21
f1 (ppm)

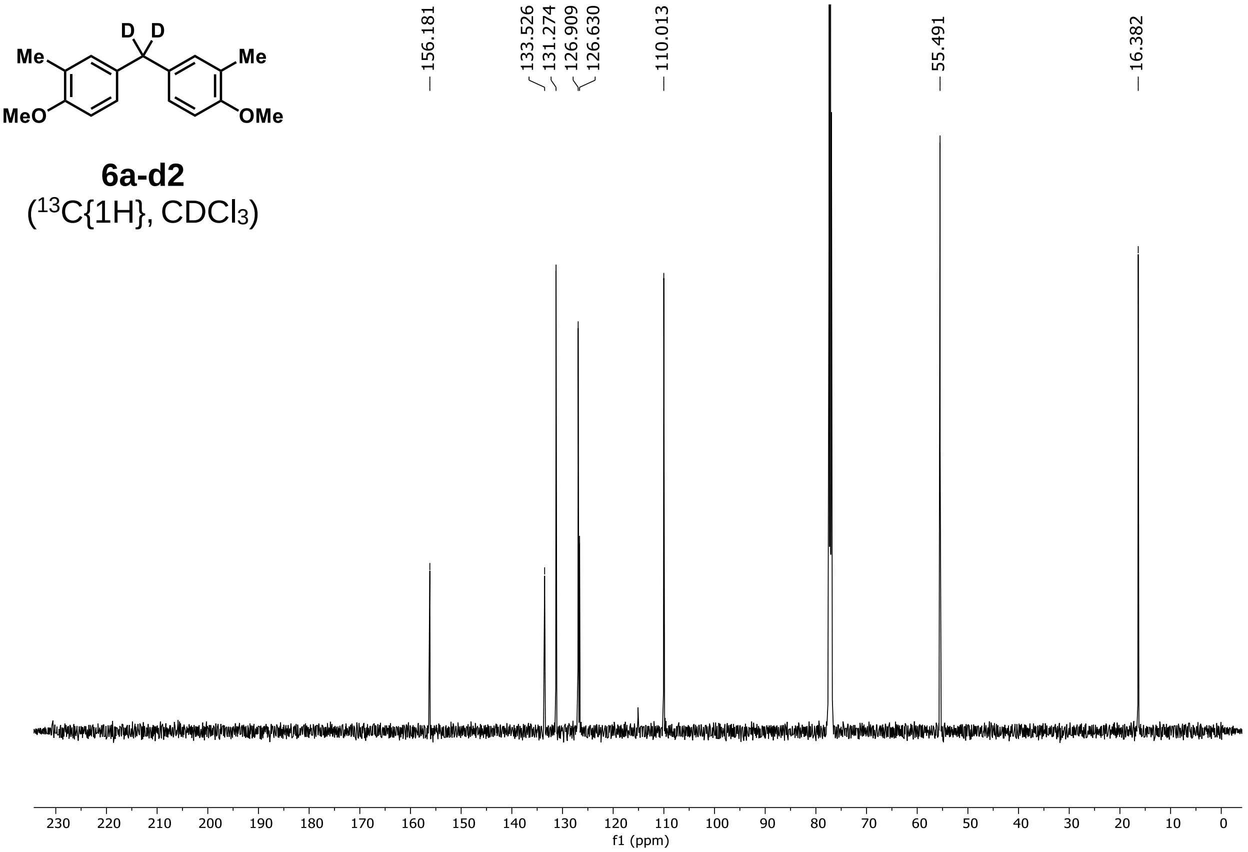
6a-d2
(13C{1H}, CDCl3)
156.181
133.526
131.274
126.909
126.630
110.013
55.491
16.382
f1 (ppm)

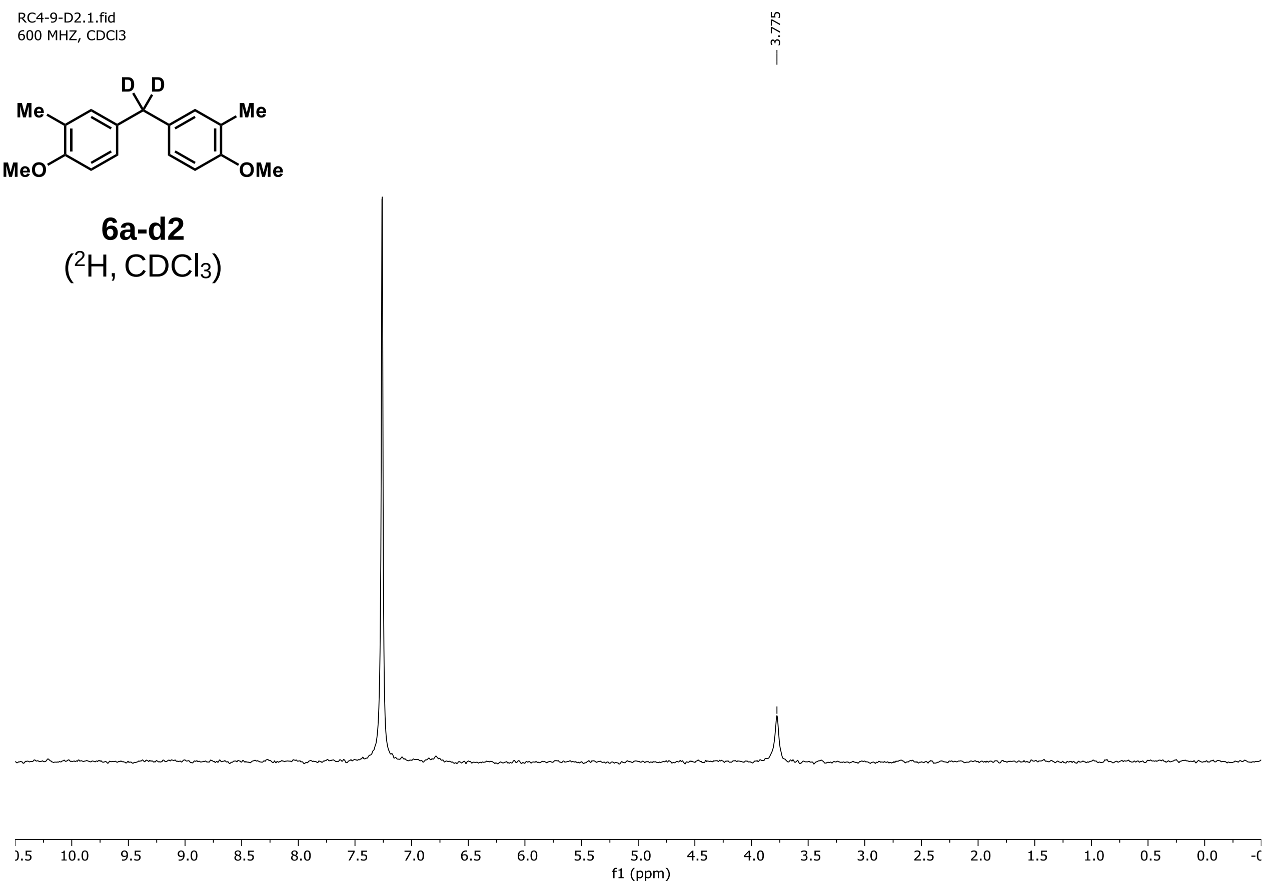
RC4-9-D2.1.fid
600 MHZ, CDCl3
3.775
D D
Me
Me
MeO
OMe
6a-d2
(2H, CDCl3)
10.0 9.5 9.0 8.5 8.0 7.5 7.0 6.5 6.0 5.5 5.0 4.5 4.0 3.5 3.0 2.5 2.0 1.5 1.0 0.5 0.0
f1 (ppm)

6zc
(^{1}H, $CDCl_3$)

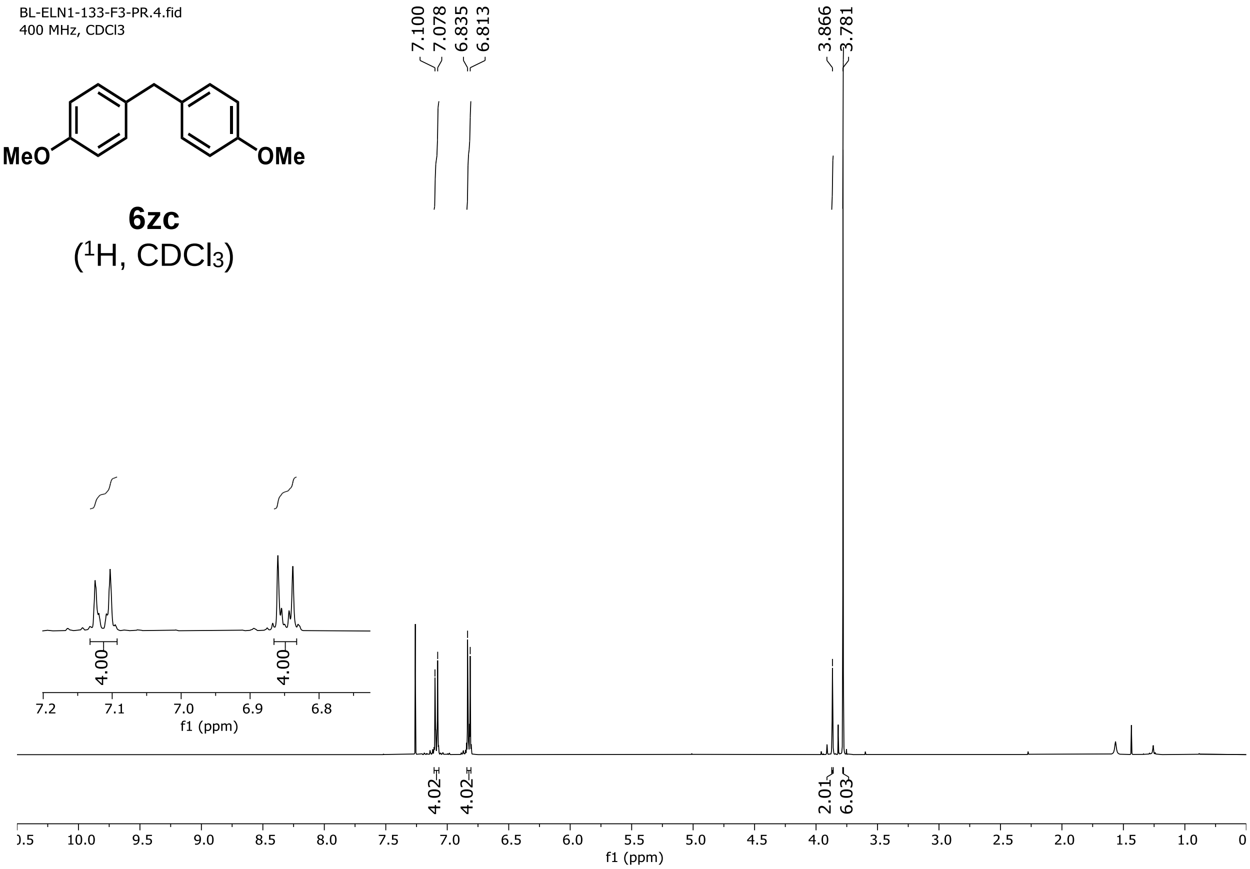

6zc
(^{13}C{1H}, $CDCl_3$)

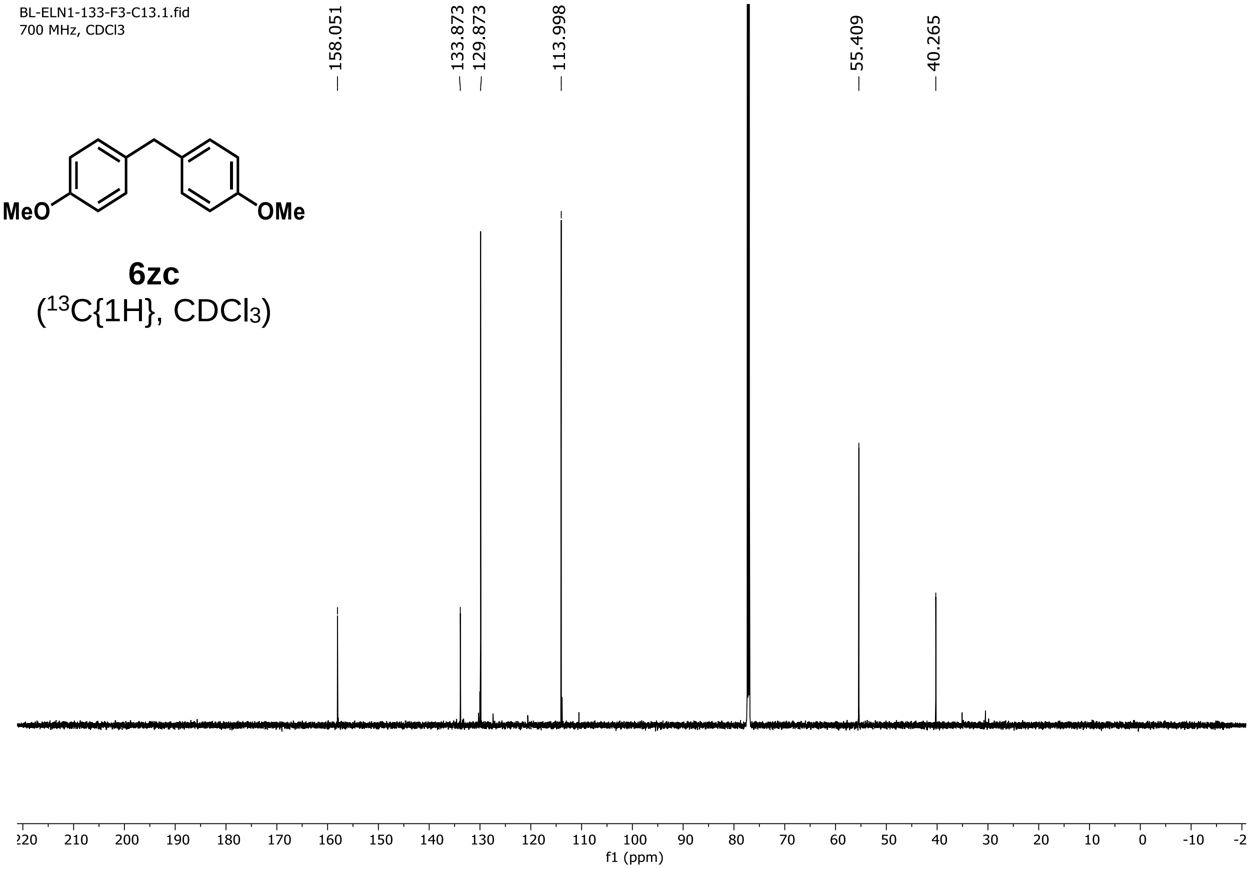

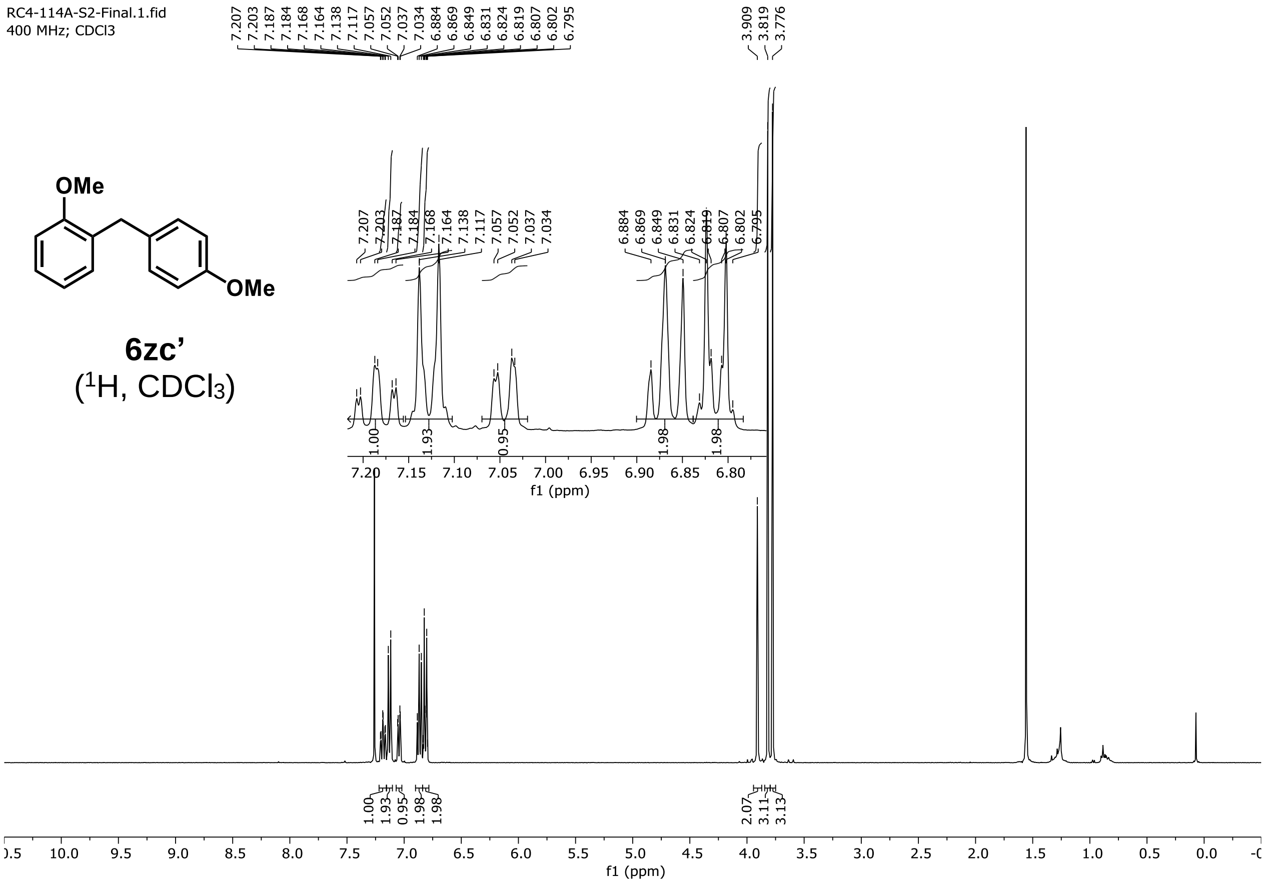

6zc'
(^{1}H, $CDCl_3$)

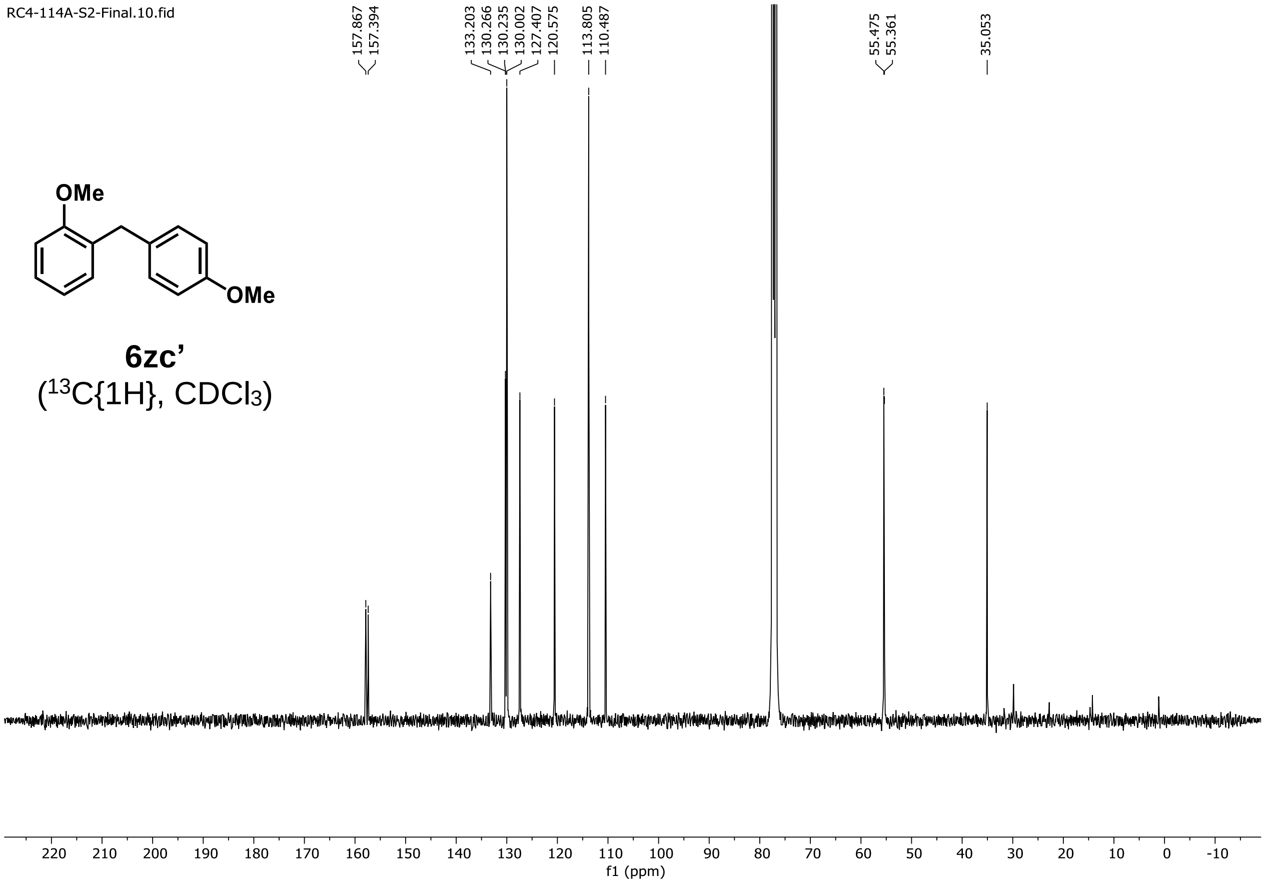

6zc'
(^{13}C{1H}, $CDCl_3$)

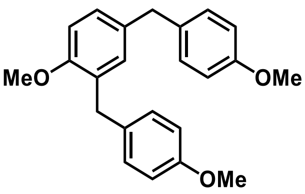

7zc
(^{1}H, $CDCl_3$)

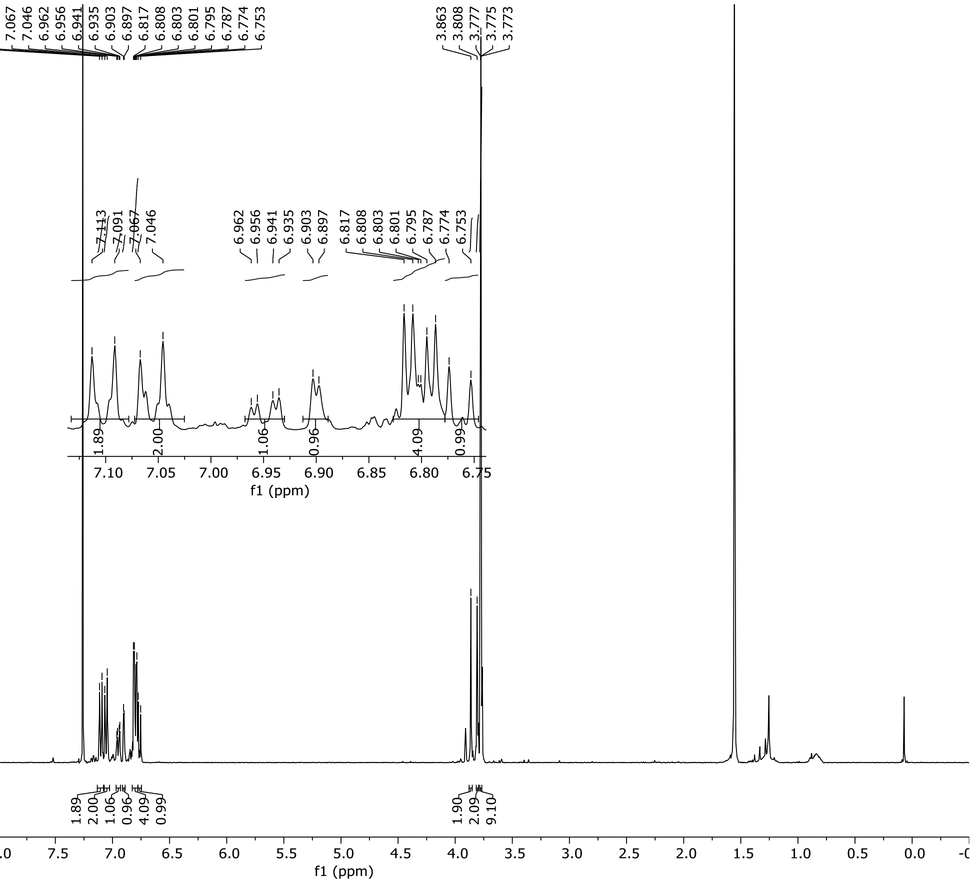

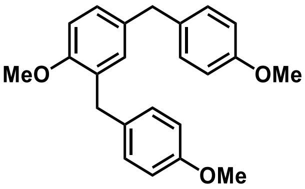

7zc
(^{13}C{1H}, $CDCl_3$)

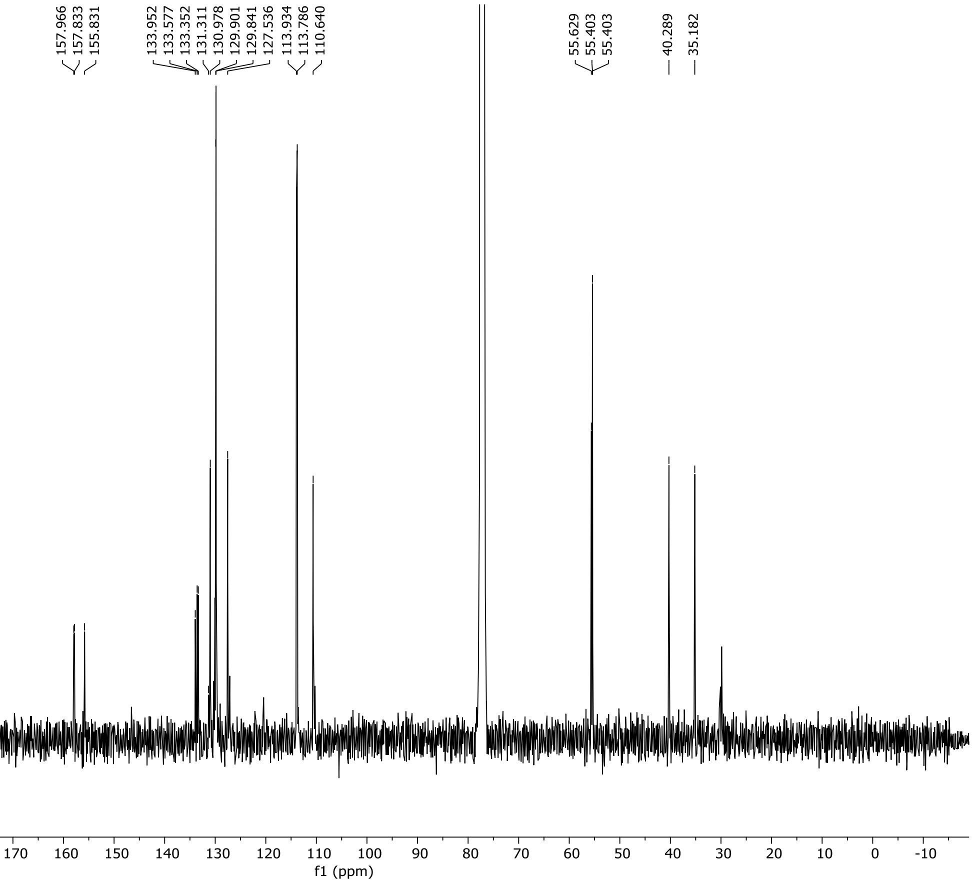

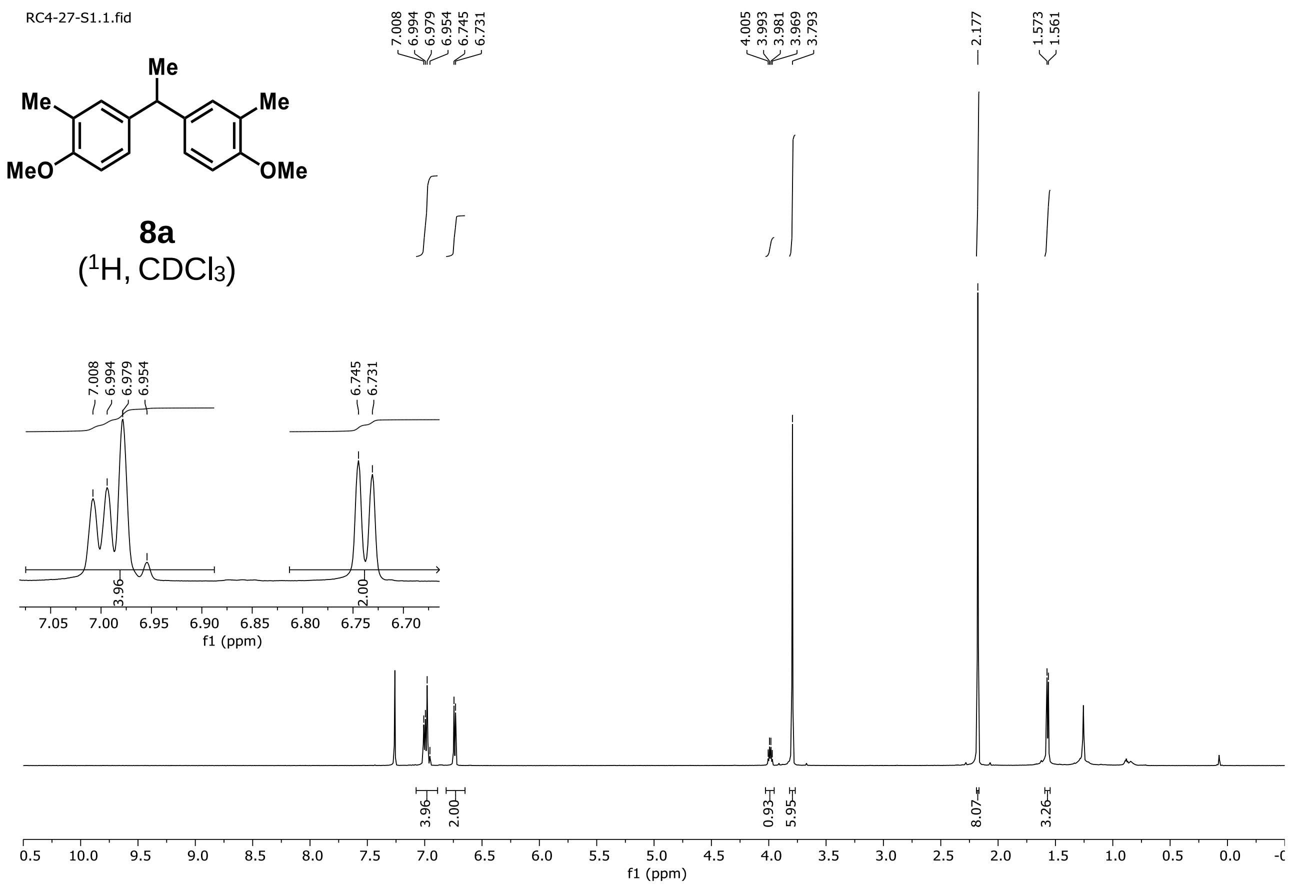
RC4-27-S1.1.fid
Me
Me
Me
MeO
OMe
8a
(1H, CDCl3)
7.008
6.994
6.979
6.954
6.745
6.731
4.005
3.993
3.981
3.969
3.793
2.177
1.573
1.561
3.96
2.00
0.93
5.95
8.07
3.26
f1 (ppm)

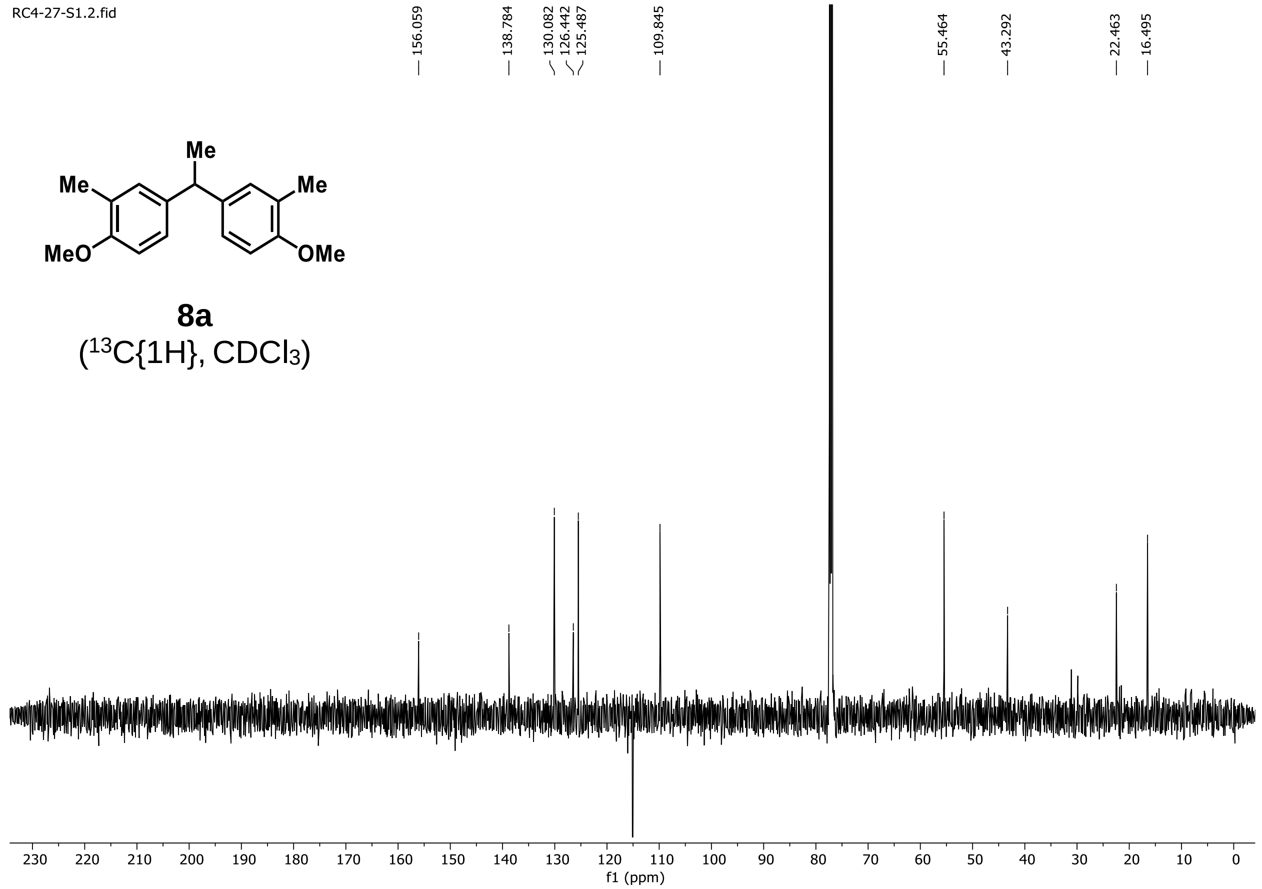
RC4-27-S1.2.fid
156.059
138.784
130.082
126.442
125.487
109.845
55.464
43.292
22.463
16.495
Me
Me
Me
MeO
OMe
8a
(13C{1H}, CDCl3)
f1 (ppm)
