# Supplementary material for: A Fenton Approach to Aromatic Radical Cations and Diarylmethane Synthesis
Source: J Org Chem. 2023 Oct 17;88(21):15060–6. doi: 10.1021/acs.joc.3c01505 (PMC10629232; doi:10.1021/acs.joc.3c01505)
Supplement: Supplementary file 2 — jo3c01505_si_002.zip [file jo3c01505_si_002.zip › FIDs/6zc-C13/1/pdata/1/email_BL-ELN1-133-F3-C13_1_1.pdf]

700 MHz, $CDCl_3$

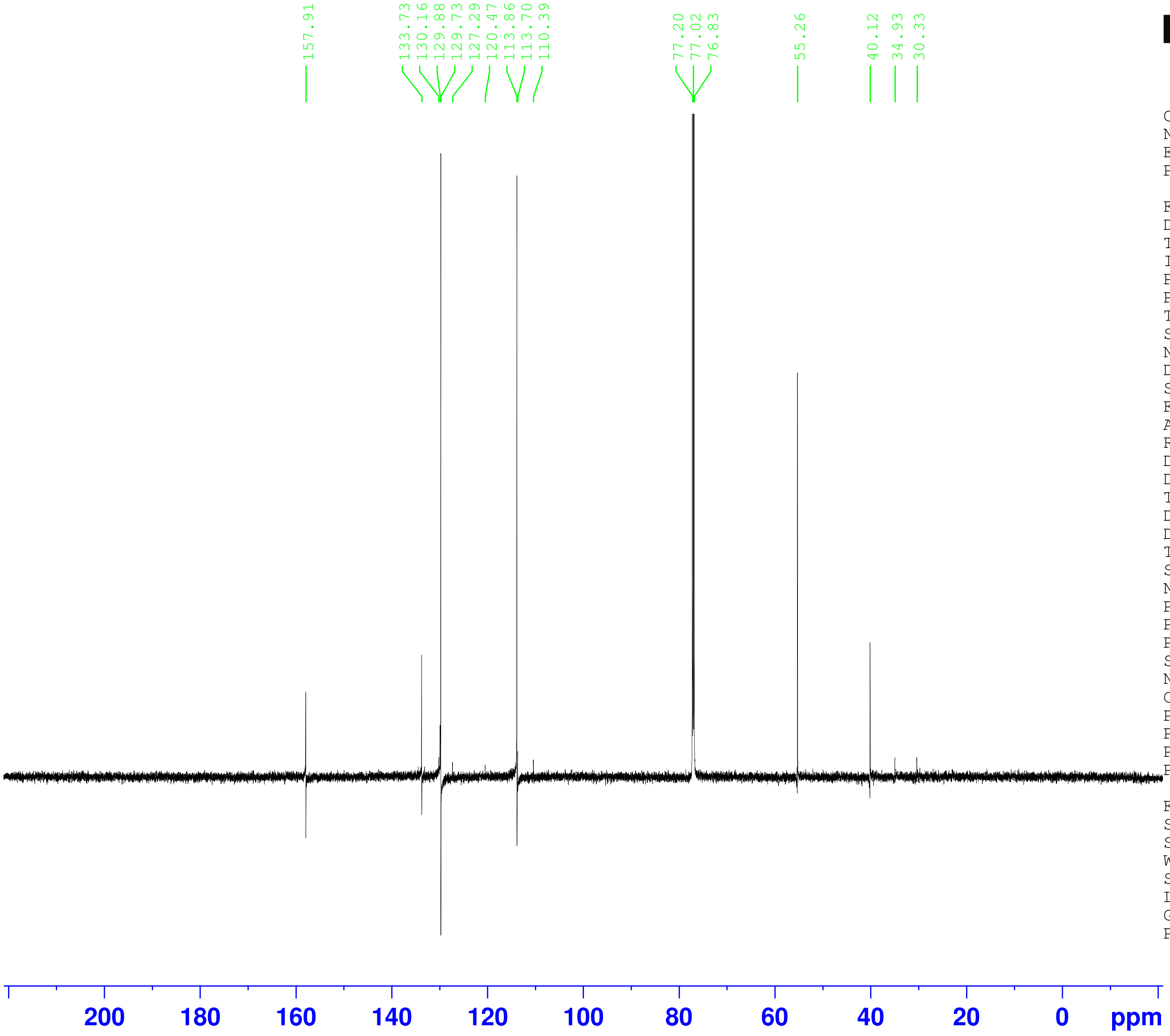

Current Data Parameters

NAME	BL-ELN1-133-F3-C13
EXPNO	1
PROCNO	1

F2 - Acquisition Parameters

Date_	20230509
Time	1.14 h
INSTRUM	spect
PROBHD	Z44908_0055 (C
PULPROG	zgpg30
TD	65536
SOLVENT	CDCl3
NS	5120
DS	4
SWH	42613.637 Hz
FIDRES	1.300465 Hz
AQ	0.7689557 sec
RG	2050
DW	11.733 usec
DE	18.00 usec
TE	298.2 K
D1	2.00000000 sec
D11	0.03000000 sec
TD0	1
SFO1	176.0905808 MHz
NUC1	13C
P0	5.00 usec
P1	15.00 usec
PLW1	75.00000000 W
SFO2	700.2328009 MHz
NUC2	1H
CPDPRG[2	waltz65
PCPD2	65.00 usec
PLW2	11.00000000 W
PLW12	0.23497000 W
PLW13	0.11832000 W

F2 - Processing parameters

SI	32768
SF	176.0729740 MHz
WDW	EM
SSB	0
LB	1.00 Hz
GB	0
PC	1.40
