# Supplementary material for: A Fenton Approach to Aromatic Radical Cations and Diarylmethane Synthesis
Source: J Org Chem. 2023 Oct 17;88(21):15060–6. doi: 10.1021/acs.joc.3c01505 (PMC10629232; doi:10.1021/acs.joc.3c01505)
Supplement: Supplementary file 2 — jo3c01505_si_002.zip [file jo3c01505_si_002.zip › FIDs/7l-C13/1/pdata/1/email_BL-ELN1-134-F3-C13_1_1.pdf]

700 MHz, CDCl3

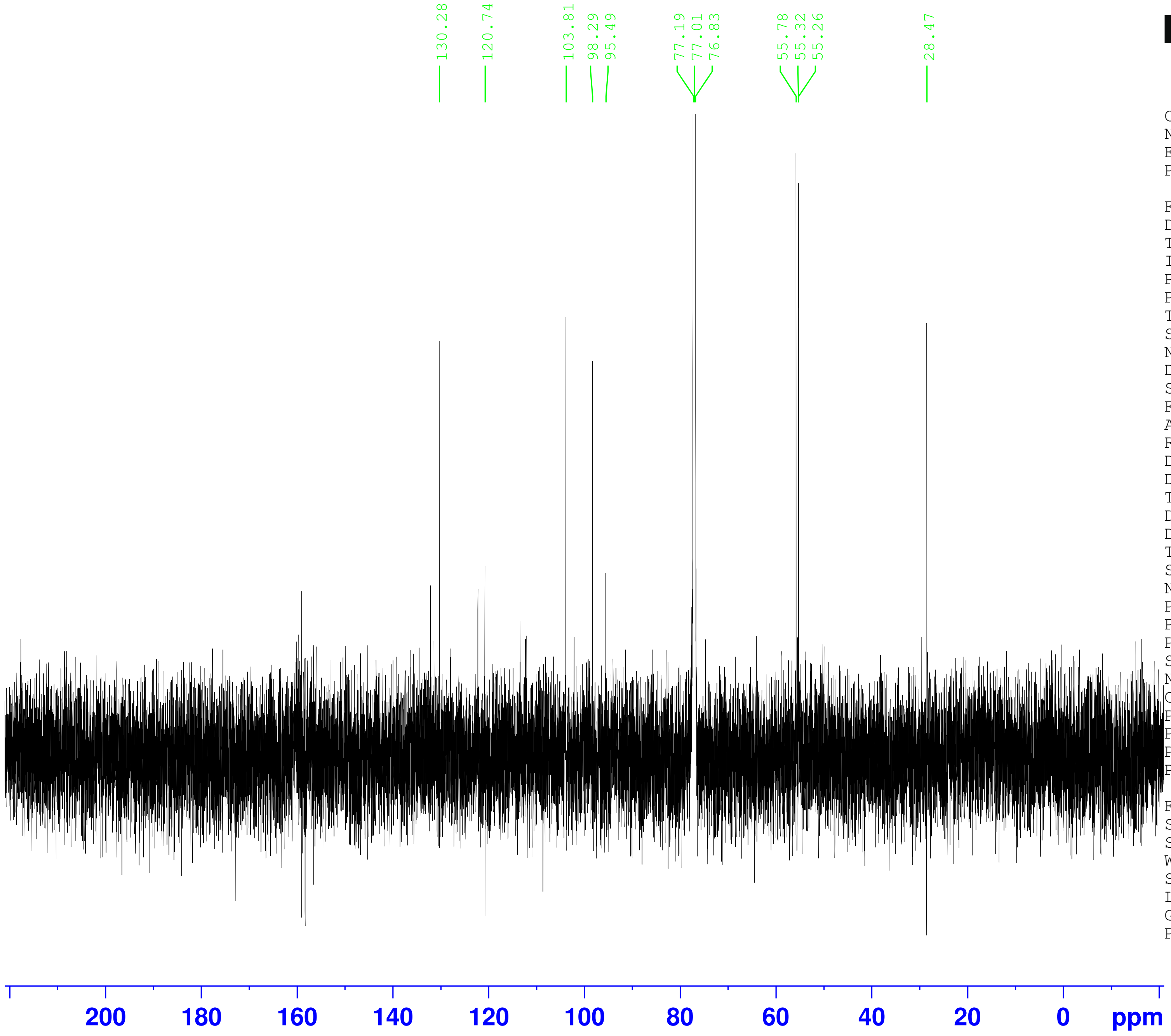

Current Data Parameters
NAME BL-ELN1-134-F3-C13
EXPNO 1
PROCNO 1

F2 - Acquisition Parameters
Date_ 20230509
Time 13.35 h
INSTRUM spect
PROBHD Z44908_0055 (C
PULPROG zgpg30
TD 65536
SOLVENT CDCl3
NS 5120
DS 4
SWH 42613.637 Hz
FIDRES 1.300465 Hz
AQ 0.7689557 sec
RG 2050
DW 11.733 usec
DE 18.00 usec
TE 298.2 K
D1 2.00000000 sec
D11 0.03000000 sec
TD0 1
SFO1 176.0905808 MHz
NUC1 13C
P0 5.00 usec
P1 15.00 usec
PLW1 75.00000000 W
SFO2 700.2328009 MHz
NUC2 1H
CPDPRG[2 waltz65
PCPD2 65.00 usec
PLW2 11.00000000 W
PLW12 0.23497000 W
PLW13 0.11832000 W

F2 - Processing parameters
SI 32768
SF 176.0729740 MHz
WDW EM
SSB 0
LB 1.00 Hz
GB 0
PC 1.40
